# Multiple interaction nodes define the postreplication repair response to UV‐induced DNA damage that is defective in melanomas and correlated with UV signature mutation load

**DOI:** 10.1002/1878-0261.12601

**Published:** 2019-12-19

**Authors:** Sandra Pavey, Alex Pinder, Winnie Fernando, Nicholas D’Arcy, Nicholas Matigian, Dubravka Skalamera, Kim‐Anh Lê Cao, Dorothy Loo‐Oey, Michelle M. Hill, Mitchell Stark, Michael Kimlin, Andrew Burgess, Nicole Cloonan, Richard A. Sturm, Brian Gabrielli

**Affiliations:** ^1^ Diamantina Institute TRI The University of Queensland Woolloongabba QLD Australia; ^2^ Mater Research TRI The University of Queensland Woolloongabba QLD Australia; ^3^ QFAB Bioinformatics The University of Queensland Brisbane QLD Australia; ^4^ QIMR Berghofer Medical Research Institute Herston QLD Australia; ^5^ University of the Sunshine Coast Sippy Downs QLD Australia; ^6^ ANZAC Research Institute Concord NSW Australia; ^7^ Present address: School of Mathematics and Statistics and Melbourne Integrative Genomics The University of Melbourne Melbourne Vic. Australia; ^8^ Present address: School of Biological Science The University of Auckland New Zealand

**Keywords:** DNA repair, G2 phase checkpoint, MASTL, postreplication repair, ultraviolet radiation

## Abstract

Ultraviolet radiation‐induced DNA mutations are a primary environmental driver of melanoma. The reason for this very high level of unrepaired DNA lesions leading to these mutations is still poorly understood. The primary DNA repair mechanism for UV‐induced lesions, that is, the nucleotide excision repair pathway, appears intact in most melanomas. We have previously reported a postreplication repair mechanism that is commonly defective in melanoma cell lines. Here we have used a genome‐wide approach to identify the components of this postreplication repair mechanism. We have used differential transcript polysome loading to identify transcripts that are associated with UV response, and then functionally assessed these to identify novel components of this repair and cell cycle checkpoint network. We have identified multiple interaction nodes, including global genomic nucleotide excision repair and homologous recombination repair, and previously unexpected MASTL pathway, as components of the response. Finally, we have used bioinformatics to assess the contribution of dysregulated expression of these pathways to the UV signature mutation load of a large melanoma cohort. We show that dysregulation of the pathway, especially the DNA damage repair components, are significant contributors to UV mutation load, and that dysregulation of the MASTL pathway appears to be a significant contributor to high UV signature mutation load.

## Graphical abstract

The UV‐G2 checkpoint is triggered by UV‐induced DNA lesions not repaired by NER during G1 phase. Global genomic NER (GG‐NER) pathway repairs these lesions during G2 phase, and homologous recombination repair (HRR) repairs the single‐stranded gaps. The MASTL pathway controls exit from the checkpoint arrest. These are all under AKT‐dependent translational control.

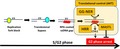

**Abbreviations**
ATM: ataxia telangiectasia mutated
ATR: ATM and RAD3‐related
CHK1: checkpoint kinase 1
GG‐NER: global genomic NER
HR: homologous recombination
NER: nucleotide excision repair
PDS: Pathway Deregulation Score
RNA‐seq: RNA sequencing
RPA: replication protein A
ssDNA: single‐stranded DNA
TCGA: The Cancer Genome Atlas
TLS: translesion synthesis
UV: ultraviolet radiation
UV‐G2 checkpoint: G2 phase checkpoint and postreplication repair response
UVR: UV radiation

## 1 Introduction

Ultraviolet radiation (UVR) is the major environmental mutagen driving the development of melanoma. The genotoxic effect of UVR characteristically results in single‐stranded DNA lesions, specifically 6–4 photoproducts and cyclobutane pyrimidine dimers (Pavey *et al.*, 2013). Melanomas commonly have very high numbers of mutations that are the direct consequence of unrepaired UV‐induced lesions (Hodis *et al.*, 2012). UVR‐damaged DNA bases are typically repaired by nucleotide excision repair (NER) (Mouret *et al.*, 2008; Pavey *et al.*, 2013). However, if the UV damage is not repaired before the cells undergo DNA replication, it can result in what has been collectively termed UV signature mutations (Helleday *et al.*, 2014). A specific mutational signature (Signature 7) secondary to unrepaired UVR damage has been found to be highly prevalent in melanomas and involves a high proportion of dinucleotide C>T mutations (Alexandrov *et al.*, 2013).

Although NER has a major role in repairing UVR damage, it is unclear as to how common NER defects are in melanomas (Belanger *et al.*, 2014; Budden *et al.*, 2016; Gaddameedhi *et al.*, 2010). A recent bioinformatic analysis of the TGCA melanoma dataset failed to find any correlation between dysregulated expression of NER pathway genes and the UV signature mutation load (D'Arcy *et al.*, 2019).

We have previously reported a G2 phase cell cycle checkpoint‐coupled repair mechanism that is triggered in response to suberythemal doses of UVR in melanocytes and keratinocytes in the epidermis (Pavey *et al.*, 2001). The G2 phase checkpoint is commonly defective in melanomas and results in cells accumulating increased UV signature mutations after irradiation (Pavey *et al.*, 2013; Wigan *et al.*, 2012). This mechanism repairs the small number of lesions remaining after NER has repaired the bulk of lesions in G1 phase. The checkpoint‐coupled repair mechanism utilizes the replication fork to interrogate the entire genome during replication to detect UV‐induced lesions and repair them in G2 phase (Wigan *et al.*, 2012). The G2 phase checkpoint is triggered by the presence of single‐stranded DNA (ssDNA) that recruits RPA and the cell cycle checkpoint kinase ATR that activates CHK1 to impose a G2 phase cell cycle arrest until the ssDNA gaps are repaired. A suite of DNA repair proteins colocalize with the RPA foci including BRCA1, RAD51, and RAD18, suggesting the involvement of homologous recombination (HR) and/or translesion synthesis (TLS) Y family polymerase mechanism in the gap filling (Chang and Cimprich, 2009; Waters *et al.*, 2009; Wigan *et al.*, 2012). How these diverse components function in this checkpoint and repair response are unclear.

Here, we have taken a genome‐wide approach to investigate this coupled G2 phase checkpoint and postreplication repair response (UV‐G2 checkpoint). We have analyzed the mRNAs differentially loaded onto polyribosomes (polysome profiling) after UVR exposure to identify differentially translated proteins. This approach was used to bridge the gap between gene expression (i.e., total mRNA RNA‐seq and microarray analysis) and proteomics analysis, as only a proportion of variation in protein abundance is explained by variations in mRNA levels (de Sousa Abreu *et al.*, 2009). We have functionally validated a large number of differentially polysome‐loaded mRNAs using a high‐throughput approach to understand the complexity of this response to UVR and identified several nodes that interact in this response. Finally, we have used pathway expression analysis to show that dysregulation of this pathway is associated with increased UV signature mutations in melanoma.

## 2 Methods

### 2.1 Cell culture

A2058 and MM576 human melanoma cell lines were cultured as described previously (Wigan *et al.*, 2012). All cell lines were confirmed to be mycoplasma free. Synchronized cell populations and UV irradiation and UV‐G2 checkpoint arrested populations were obtained as previously described (Wigan *et al.*, 2012). For cellular DNA content analysis, floating and adhered cells were collected and fixed in 70% ethanol at −20 °C and analyzed by flow cytometry using a FACSCalibur system (BD Biosciences, San Jose, CA, USA). Data analysis was performed using flowjo x software (Becton Dickson, Ashland, OR, USA).

### 2.2 Immunoblotting

Cells were lysed and analyzed by immunoblotting as described previously (Wigan *et al.*, 2012). Antibodies against DDB1, POLG (LS Bioscience, Seattle, WA, USA), NCOR2/SMRTE, SCRIB (Santa Cruz Biotechnology, Dallas, TX, USA), XPC (GeneTex, Irvine, CA, USA), SMARCA4, MTAP (Abcam, Cambridge, UK), STAT3 (Becton Dickson, North Ryde, NSW, Australia), GDF15 (R&D Systems, Minneapolis, MN, USA), MASTL (Millipore, Bayswater, VIC, Australia), pSTAT3 Y705, B55α, ENSA, pMEK Thr286, pCDK Tyr15 (Cell Signaling Technology, Danvers MA, USA), PCNA (Dako Agilent, Santa Clara, CA, USA), and α‐tubulin (Sigma‐Aldrich, St Louis, MO, USA) were purchased from the indicated suppliers. Rabbit antibody to the N‐terminal peptide (residues 1–15) of human ARPP‐19 (GenScript, Piscataway, NJ, USA) was produced and affinity‐purified against the antigen peptide. Proteins were visualized using the appropriate horseradish peroxidase‐conjugated secondary antibodies and enhanced chemiluminescent detection. Protein levels were quantified using image j/fiji software (Schindelin *et al.*, 2012).

### 2.3 Preparation of polysome fraction

A2058 cells were grown as asynchronous cultures, treated with UVB radiation, or synchronized in G2 phase of the cell cycle as described above. At the time of harvest, media were replaced with prewarmed media containing 100 µg·mL^−1^ cycloheximide for 3 min at 37 °C. Cells were then placed on ice and washed twice with ice‐cold PBS with 100 µg·mL^−1^ cycloheximide. Cells were harvested and 5% of cells were fixed in 70% EtOH for FACS analyses, and the remainder of the cells were lysed with freshly prepared ice‐cold lysis buffer (2 mm HEPES pH 7.6, 125 mm KCl, 5 mm MgCl_2_, 0.5% NP40, EDTA‐free Protease Cocktail Inhibitor (Roche, Sydney, NSW, Australia), 100 µg·mL^−1^ cycloheximide, 2 mm DTT, 0.5 mm PMSF, and 100 U·mL^−1^ RNase Inhibitor) and incubated for 10 min on ice. Samples were centrifuged at 12 000 ***g***/10 min/4 °C, and supernatant was retained. Simultaneously, a control was constructed containing 30 mm EDTA to dissociate the polysomes. Sucrose gradients (10 mL, 17.5–50% sucrose) were prepared using the ISCO gradient former with Beckman 13.2 mL ultraclear tubes (#344059), containing 15.5% or 50% sucrose, 20 mm HEPES, 125 mm KCL, 5 mm MgCl_2_, 100 µg·mL^−1^ cycloheximide, 2 mm DTT, and 0.5 mm PMSF. Protein was loaded onto each gradient and centrifuged for 2:15 h in an ultracentrifuge (Beckman ultracentrifuge Optima L‐90K) with SW41 Ti rotor at 4 °C with maximum acceleration and no brake. Following centrifugation, 24 fractions were collected from each gradient using the ISCO model density gradient fractionator, connected to a UA6 ultraviolet detector, which recorded an absorbance profile at 254 nm. Each fraction was spiked with a foreign (control) RNA to equilibrate between samples. Each fraction was spiked with a *B. subtilis* RNA mix consisting of TRP 80 pg·µL^−1^, Lys 160 pg·µL^−1^, Thr 240 pg·µL^−1^, and Phe 320 pg·µL^−1^ (ATCC, Manassas, VA, USA). To each fraction, 2 µL of GlycoBlue coprecipitant (50 µg·mL^−1^) was added and mixed, followed by 3 volumes of 100% ethanol, and RNA was precipitated at −80 °C overnight to remove the sucrose. RNA was resuspended in RNase‐free water and fractions comprising polysome‐bound mRNAs were pooled, and RNA was extracted using TRIZOL LS, as per the manufacturers’ instructions, followed by a final lithium chloride precipitation. Total RNA was also extracted from the cell lysate that was used to load onto the gradient. RNA concentration and integrity were examined on a NanoDrop 1000 spectrophotometer (Thermo Fisher Scientific, Waltham, MA, USA) and 2100 Bioanalyzer (Agilent Technologies, Santa Clara, CA, USA).

### 2.4 Microarray gene expression profiling and RNA‐Seq

Whole‐genome gene expression was examined using Illumina HumanHT‐12 v3 Expression BeadChips, as per manufacturer’s instructions. RNA‐Seq was performed using the Illumina TruSeq mRNA Library Preparation Kit, according to the manufacturer’s instructions. Sequencing of the libraries was performed on an Illumina HiSeq 2000 sequencing instrument at the University of Queensland Diamantina Institute.

### 2.5 siRNA and lentiviral overexpression functional screen

Cells were reverse‐transfected with pooled siRNAs (On‐Target Plus Smartpools, GE Healthcare Dharmacon, Lafayette, CO, USA) for knockdown of the 42 unique genes assessed in this study (Table S7), along with deconvoluted siRNAs for ARPP‐19 depletion (#5, #6, #7, and #8). Transfection was performed using Dharmafect 2 (GE Healthcare Dharmacon) as transfection reagent (Wigan *et al.*, 2012). Detailed transfection protocol and method followed for high‐throughout screening and high‐content analysis are presented in Supplementary Methods. For the study of the MASTL pathway, cells were transfected 24 h before irradiation, and then harvested at 24–28 h postirradiation for immunoblotting and flow cytometry. Gateway entry clones from the polysome gene list present in the human ORFeome library (Skalamera *et al.*, 2012; Table S7) were cloned into pLEX307 (Addgene plasmid # 41392) that introduces a V5 tag at the 3′ end of the ORF. Lentivirus was produced as previously described (Skalamera *et al.*, 2012) and used to transduce A2058 and MM576 cells. Four days after transduction, plates were irradiated in HBSS and then replaced with complete media. Plates were fixed for immunostaining as described for siRNA screening.

For siRNA depletion, cells were reverse‐transfected into 24‐well plates. For MM576 cells, culture was seeded at 12 500 cells per well with the addition of DharmaFect2 at 0.5%. For A2058 cell line, culture was seeded at 125 00 cells per well and Lipofectamine 2000 was added at 0.3%. A final volume of 500 µL for each transfection process was attained by the addition of 80 µL of siRNA/lipid/optimum and 420 µL of RPMI complete media. Plk1 and NT siRNAs were used as controls. All siRNAs were used at 10 nm final concentration. Each plate contained three replicates of each of the control siRNA Plk1, NT, and cells only, plus gene of interest targeting siRNAs. On Day 2, media was changed and replaced with RPMI complete media. Day 3: UV radiation in 180 µL per well HBSS with 250 Jm^−2^ UVB as in Wigan *et al. *(2012). Replaced with fresh complete media. On Day 4, the cells were harvested 24 h post‐UVR exposure and from non‐UVR control plates (72 h post‐transfection). On Day 5, cells were harvested after 40 h of UVR treatment (88 h post‐transfection). In each case, cells were fixed with 3.7% PFA for 15 min (300 µL per well). Cells were then permeabilized with 0.1% Triton‐X in PBS for 15 min (300 µL per well), and then blocked in 1.5% BSA in PBST (BB) for 2 h at RT (300 µL per well). Primary antibody (RPA34‐19; Merck Millipore) was used at 1/1000 dilution in BB and incubated overnight 4 °C (180 µL per well) in a humidified chamber, and then plates were washed and further incubated with secondary antibody (anti‐mouse Alexa 488; Invitrogen, Carlsbad, CA, USA) at 1/500 dilution overnight at 4 °C (180 µL per well) as above. Finally, the cells were washed and stained with 600 nm DAPI in BB for 3 h at room temperature, and stored in 750 µL per well PBS. Plates were imaged using InCell2200 at 40× magnification, with 49 fields per well (spaced and centered). High‐content image analysis for nuclear DNA content and RPA2 foci number was performed on cells fixed at either 24 or 40 h postirradiation or controls, and immunostained with RPA2 antibody (Santa Cruz) and DAPI for determining the DNA content. Cells were imaged using InCell Analyzer v2200 (GE Healthcare, Colorado Springs, CO, USA) at 40× magnification, with 49 fields per well (spaced and centered). Images were analyzed using incell analyzer v2200 software with multitarget analysis segmentation/quantification protocol. DAPI signal was used to generate nuclear mask and RPA2 signal to threshold foci. Data collected from images were further processed using r software (https://www.r-project.org/). In some experiments, cells were transfected and then irradiated as above, and then followed by time‐lapse microscopy as described previously**.**

For lentiviral transductions, Gateway entry clones from the polysome gene list present in the human ORFeome library (see Table S7 for the gene list) were LR cloned into pLEX307 (Addgene plasmid # 41392) donor lentiviral vector that introduces a V5 tag at the 3′ end, and EF1‐alpha promoter at the 5′ end of the ORF. Lentivirus was produced as previously described and used to transduce A2058 and MM576 cells. Four days after transduction, plates were irradiated in HBSS and then replaced with complete media. Plates were fixed at 24 and 40 h after irradiation for immunostaining, as described for siRNA screening On Day 1, cells were seeded into 24‐well plates (MM576: 9000 cells/well; A2058: 8000 cells per well) containing 720 µL per well of complete RPMI media. The following day, cells were transduced by the addition of 170 µL of viral supernatant plus 21 µL of polybrene (120 µg·mL^−1^)/well, one gene per well. Cells were incubated with this transduction mix for 1.5 h, and then each well was topped up with 720 µL of complete RPMI media/well. Each plate also contained three wells of cells only and two wells of empty vector controls (Poly). One plate was prepared for each condition: no UV exposure, UV exposure for 24 h, and UV exposure for 40 h. On Day 3, media was changed in each well and 700 µL of fresh complete RPMI media was added. On Day 6, 96 h after transduction, plates were UV irradiated. Media were removed and replaced with 150 µL of HBSS/well and irradiated as above for siRNA screening. No UV control plates were fixed at this time. On Day 7, UV 24‐h plates and on Day 8 UV 40‐h plates were fixed using 3.7% PFA for 15 min. Cells were then washed, permeabilized, and blocked as above. Anti‐rabbit V5 (Invitrogen) was used at 1/2000 dilution in 3% BSA prepared in Tris buffer saline (0.1%), incubated overnight at 4 °C, stained, imaged, and analyzed as above.

### 2.6 Pathifier pathway analysis of UV‐G2 checkpoint genes

Data from the TCGA melanoma dataset (Cancer Genome Atlas Network, 2015) was used to analyze a combined list of those genes identified as contributors to the UV‐G2 checkpoint pathway using the ‘Pathifier’ algorithm. These genes were put into a G2 checkpoint or repair pathway and analyzed with the ‘Pathifier’ algorithm. Prior to analyses, data were filtered to only include malignant melanoma NOS, nodular melanoma, and samples with complete RNA‐Seq and UV signature mutation data (D'Arcy *et al.*, 2019). This dataset was also assessed for any confounding variables including body site, age, gender, and subtype. Pathifier transforms gene expression (RNA‐Seq) into pathway‐level information to model a pathway deregulation score (PDS) for each sample (D'Arcy *et al.*, 2019; Drier *et al.*, 2013). The PDS for each sample was related to the number of UV signature mutations in that sample (Alexandrov *et al.*, 2013). The samples were divided into UV mutational load subgroups, and the PDS was compared between the groups as described previously (D'Arcy *et al.*, 2019). The samples were subgrouped into high, mid, and low USM load as described previously: high, >54.6 mutations per MB (*n* = 32); mid, 3.6–54.6 mutations per MB (*n* = 250); low, >3.6 mutations per MB (*n* = 58); and zero mutations (*n* = 12).

## 3 Results and Discussion

### 3.1 Polysome‐bound RNA analysis of G2 phase response to UVR

**Figure 1:**
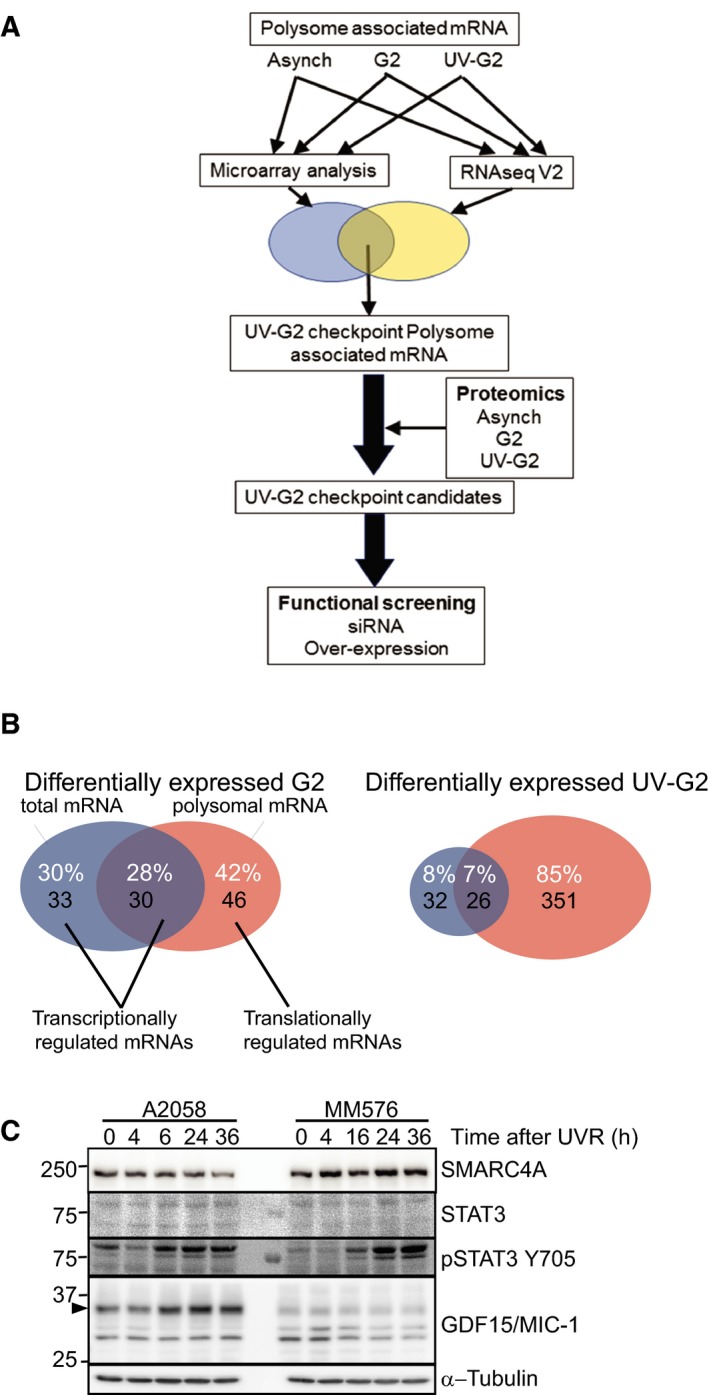
Identification of candidate gene involved in the UV‐G2 checkpoint response. (A) Schematic of the analysis pipeline developed to identify components of the UV‐G2 checkpoint response. (B) Venn diagram showing the overlap between differentially expressed total and polysome‐loaded mRNA in the UV‐G2 checkpoint. (C) Immunoblot analysis of proteins identified as differentially loaded onto polysomes and in proteomics analysis of UV‐G2 checkpoint arrest of A2058 and MM576 cells. The arrow head indicated the precursor form of GDF15.

To define components of the UV‐G2 checkpoint, we have identified mRNA species that are differentially loaded onto polysomes in UV‐G2 checkpoint arrested A2058 melanoma cells that have a functional UV‐G2 checkpoint (Wigan *et al.*, 2012). The mRNA from irradiated cells was compared to asynchronously growing cells, and to a G2 phase‐enriched population to ensure that the differential loading is associated with the checkpoint and not simply G2 phase (Fig. S1). From these samples, total mRNA and polysome‐bound mRNA were prepared in biological replicates for microarray and RNA‐seq analysis of polysome‐bound mRNA. The candidate selection pipeline to identify components of the UV‐G2 checkpoint response is shown in Fig. 1A.

Differentially expressed transcripts, both total and polysome‐bound, were identified for each of the G2 phases and UV‐G2 checkpoint samples by comparing with the asynchronous transcript levels. Using a fold change cut off of 2 and B statistic > 0 produced lists of 66 and 60 differentially expressed genes, and 76 and 379 differentially polysome‐bound transcripts in the G2 phase and UV‐G2 checkpoint samples, respectively (Tables S1–S4). The differentially expressed G2 phase transcripts that had a similar fold change in both G2 phase and UV‐G2 checkpoint lists were removed, reducing the differentially polysome‐bound UV‐G2 list to 346 transcripts. When the transcriptionally regulated (total mRNA) and polysome‐bound lists were compared, 42% of transcripts were only translationally regulated in G2 phase, whereas this increased to 85% in the UV‐G2 cells. This was due to an extraordinary increase in the number of translationally regulated transcripts; the number of transcriptionally regulated transcripts was similar in the two conditions (Fig. 1B).

The polysome‐bound RNA was also analyzed by RNA‐seq. The differentially expressed UV‐G2 checkpoints were determined as for the microarray analysis, removing those genes with similar fold changes in G2 samples (Table S5). This gene set was completely contained within microarray analysis. Together, these analyses yielded 306 high‐confidence UV‐G2 transcripts (Table S6).

About 65% of the differentially loaded polysome‐associated mRNAs in the UV‐G2 checkpoint samples were not altered at the total mRNA level, similar to the proportion of transcripts differentially loaded onto polysomes in response to EGF treatment or hypoxia (Lai *et al.*, 2016; Tebaldi *et al.*, 2012). Interestingly, 70% of differentially expressed mRNAs in the UV‐G2 checkpoint samples did not show a corresponding change in polysome loading, suggesting that other mechanisms were regulating the translation of these transcripts, possibly miRNA (Selbach *et al.*, 2008). This suggests that acute events such as UVR, hypoxias or EGF signaling utilize a greater degree of post‐transcriptional regulation than was previously realized.

We assessed the changes in protein levels of nine of the transcripts in two melanoma cells lines (A2058 and MM576) with a functional UV‐G2 checkpoint. Immunoblotting at different time intervals following UVR showed that the full‐length precursor form of GDF‐15 (35 kDa band; Fig. 1C) accumulated with time in A2058 cells, and a more modest increase in the precursor form of GDF‐15 was observed in MM576 cells. Changes in lower molecular weight forms are likely to represent proteolytically cleaved forms (Li *et al.*, 2018). A similar modest increase in STAT3 protein level and a strong increase in STAT3 Y507 phosphorylation was observed, suggestive of activated JAK/STAT3 signaling that was only apparent at 16 h after irradiation when cells were accumulating the G2 phase delay (Fig. 1C). When compared to synchronized cell cycle fractions, POLG, NCOR2, DDB1, and SCRIB levels in the UV‐G2 samples were less than the corresponding G1‐ and G2 phase samples in both cell lines (Fig. S2), corresponding to the decreased polysome loading in UV‐G2 samples (Table S5), although levels of XPC and SMARCA4 levels were unchanged. These data indicated that there was a good concordance between the changes following mRNA loading onto polysomes and changes in protein level.

### 3.2 Functional analysis of the differentially loaded mRNA

From these datasets, a list of candidate genes with potential functions in DNA damage responses and cell cycle control was developed for functional testing in two UV‐G2 checkpoint functional cell models, A2058 and MM576 (Table S7). A set of 42 transcripts increased on polysomes were screened by siRNA depletion in both cell lines to determine their involvement in the UV‐G2 phase checkpoint response.

Optimal transfection conditions were established for both cell lines (Fig. S3). After transfection, cells were irradiated with 150 Jm^−2^ UVB radiation and then assessed at two time points after irradiation to examine the effect on cell survival (cell number), G2 phase delay by DNA content, DNA repair by RPA2 foci number, and total RPA intensity were assessed by high‐content image analysis (Fig. S4). The two time points represent the UV‐G2 checkpoint arrest (increased 4n DNA content at 24 h), and recovery from the arrest (decreased 4n and increase 2n DNA peaks at 40 h after UV exposure; Fig. S5).

**Figure 2:**
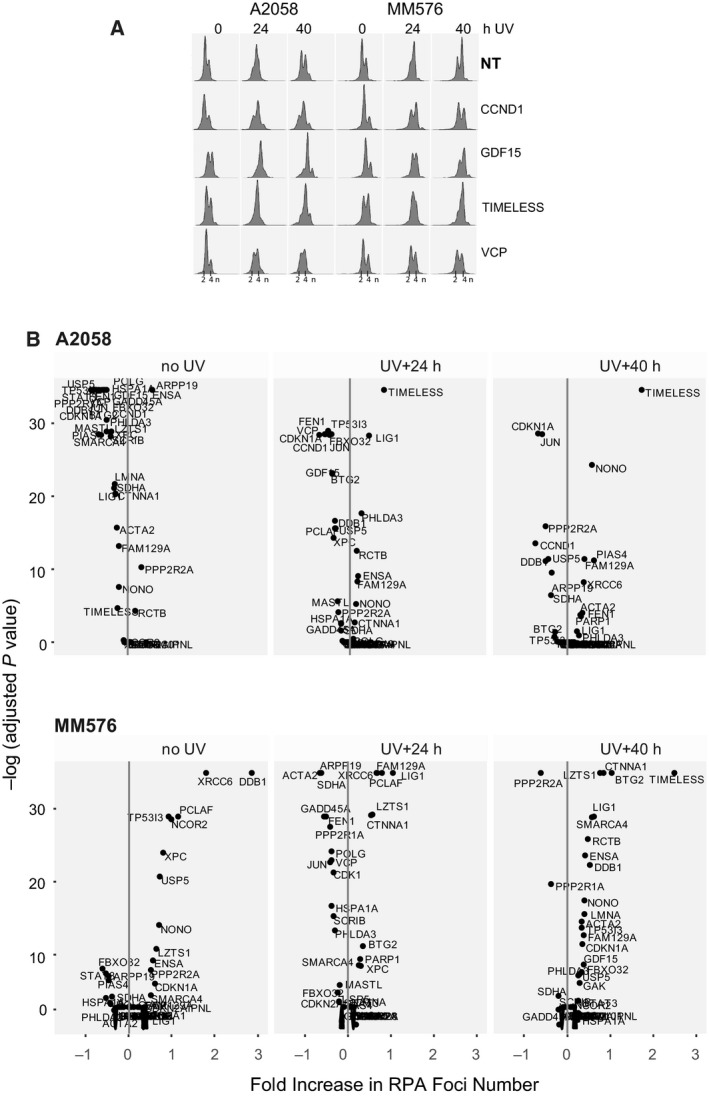
Functional analysis of UV‐G2 checkpoint candidates. (A) Examples of DNA content (X‐axis fluorescence intensity scale, log transformed, arbitrary units) density plot for A2058 and MM576 cells transfected with siRNA SmartPools directed against the indicated genes (NT nontargeting). The full dataset is presented in Fig. S7. (B) Plots of the fold change in RPA foci numbers per cell for siRNA depletion of each UV‐G2 checkpoint candidate gene relative to the nontargeting control, against the adjusted p value (Tukey HSD test) for each time point. Only A2058 data are shown. Both cell lines are shown in Fig. S8.

The effect of siRNA depletion was stronger in the A2058 than in the MM576 cell line, most likely due to the higher transfection rates of A2058 cells (Fig. S6). The majority of siRNA agents had little effect on either cell number, cell cycle, or RPA foci number either without or following UVR. SiRNA directed against UBC was toxic as it strongly reduced cell numbers in all samples and was not considered further (Fig. S6). VCP siRNA also reduced cell numbers, especially after UV treatment. Analysis of the cell cycle distribution using DNA content measured by high‐content imaging showed there was a reduced S/G2 (4n) compartment after irradiation compared to the nontargeting control (Fig. 2A), and the reduction in cell number at this time suggests that loss of VCP may sensitize cells to killing during progression through S/G2 phase after irradiation. Depletion of CCND1 and GDF15 reduced cell numbers substantially below the nontargeting control siRNA in both cell lines (Fig. S6), and resulted in delayed progression into the G2 phase arrest at 24 h and exit from the arrest at 40 h (Fig. 2A; Fig. S7 for full data set). This is likely to be due to reduced proliferation and the proportion of cells actively progressing through S phase. This was confirmed in a subsequent siRNA experiment where both CCND1 and GDF15 siRNAs reduced the S/G2 compartment of unirradiated cells, and delayed exit from the arrest at 40 h. The remaining siRNAs that affected cell cycle were BTG2, CDK1, FEN1, LZTS1, MASTL, NONO, PIAS4, PPP2R2A, SDHA, SMARCA4, and TIMELESS, all of them delayed cells in S/G2 at 40 h after UVR in both cell lines (Fig. 2A; Fig. S7).

Analysis of the changes in RPA foci numbers after UVR revealed striking increases at both time points after UVR with depletion of TIMELESS and LIG1 (Fig. 2B; Fig. S8). Statistical analysis identified several genes that were significantly altered, although most had changes of less than 2‐fold (Fig. 2B; Fig. S8). NONO, RCTB, PHLDA3, FAN129A, CTNNA1 and ACTA2 also showed consistent modest increases in the number of RPA foci after UVR, suggesting delayed repair of the single‐stranded DNA gaps produced as a consequence of bypass of unrepair UV‐induced DNA lesions (Wigan *et al.*, 2012). Interestingly, deletion of the MASTL‐ARPP‐19‐PPP2R2A (B55α) pathway components reduced the number of RPA foci in both cell lines after UVR, as did FEN1, JUN, VCP, FBXO32, and CDKN1A, suggesting reduced ability to detect single‐stranded DNA and form RPA foci or premature exit from the G2 phase checkpoint arrest resulting in the loss of RPA foci (Zuazua‐Villar *et al.*, 2014).

These parameters were scored for each gene using the scheme outlined in Table S8 to give a combined score for the effect of siRNA depletion of each gene in each cell line. This analysis identified a set of high‐confidence genes (score ≥ 10 for both cell lines) and a lower confidence group (score ≥ 10 in one cell line; Table 1).

**Table 1:**
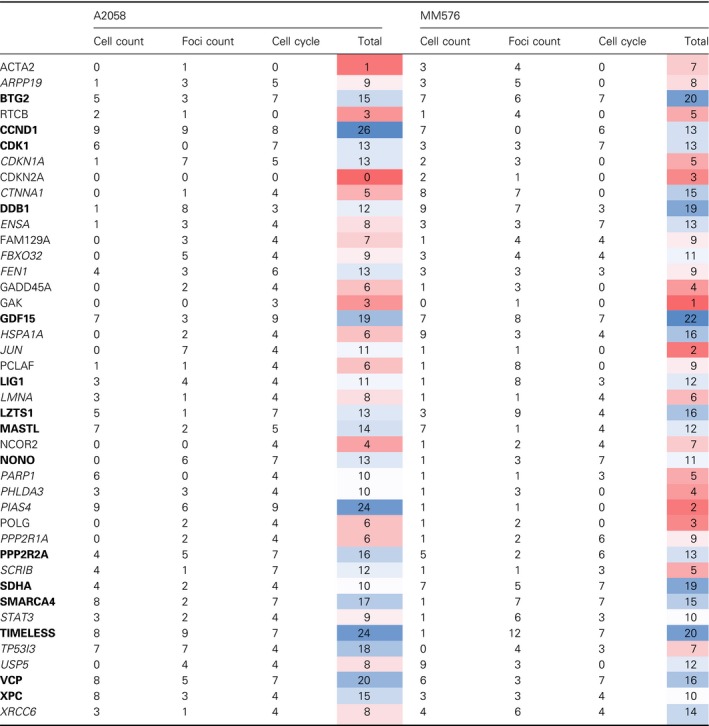
Results of siRNA screen.

Bolded genes, high confidence—total score ≥ 10 in both cell lines (top half of scores). Italic genes, lower confidence—total score ≥ 10 in only one cell line. Scoring scheme is outlined in Table S9. The heat maps indicates lowest (red) and highest (blue values).

To investigate the genes downregulated on polysomes after UVR, a subset of 51 genes from the polysome list were subcloned into a Gateway lentiviral expression vector which introduces a V5 epitope tag (Table S7). Several genes were represented by two independently assessed clones. V5 tag in the transduced A2058 and MM576 cells was detected by immunolabelling and used to select cells expressing the target gene. Tag localization and nuclear DNA content were assayed similar to the siRNA experiment following UV exposure. Where there were < 500 transduced cells at all time points, no cell cycle analysis was reported. Irradiation of cells overexpressing DDB1, FAM129A, FBXO32, GADD45A, LIG1, LZTS1, SCRIB, TP53I3, VCP, NONO, and PIAS4 reduced the percentage of transduced cells by > 25% of the unirradiated controls in both cell lines by 40 h post‐UVR, suggesting that overexpression of these genes reduces the viability of the transduced population following UVR (Fig. S9). CDKN1A, JUN, PCLAF and PHLDA3 affected cell cycle progression from the G2 phase checkpoint arrest in both cell lines (Fig. S10). Using the scoring outlined in Table S8, a set of genes with high confidence (score > 2 in both lines) and low confidence (score > 3 in one cell line) was defined (Table 2). The cellular localization of the overexpressed proteins in most cases was predominantly nuclear, although FAM129A, GDF15, LARGE, MGP, and UCN2 all displayed cytoplasmic staining, LARGE1 appearing to localize to specific structures in the cytoplasm, likely to be the Golgi (Brockington *et al.*, 2005; Fig. S11A; Table S9). LZTS1 stained cytoplasmic fibers, and BMI1 accumulated in large nuclear foci which did not change with UVR and did not colocalize with RPA foci (Fig. S11B). UVR treatment did influence the localization of LIG1 which formed foci that colocalized with RPA foci. HSPA1A had reduced nuclear localization and STAT3 increased nuclear localization after UVR. The predominantly cytoplasmic PPP2R1A showed increasing nuclear localization after UVR (Fig. S11C,D).

**Table 2:**
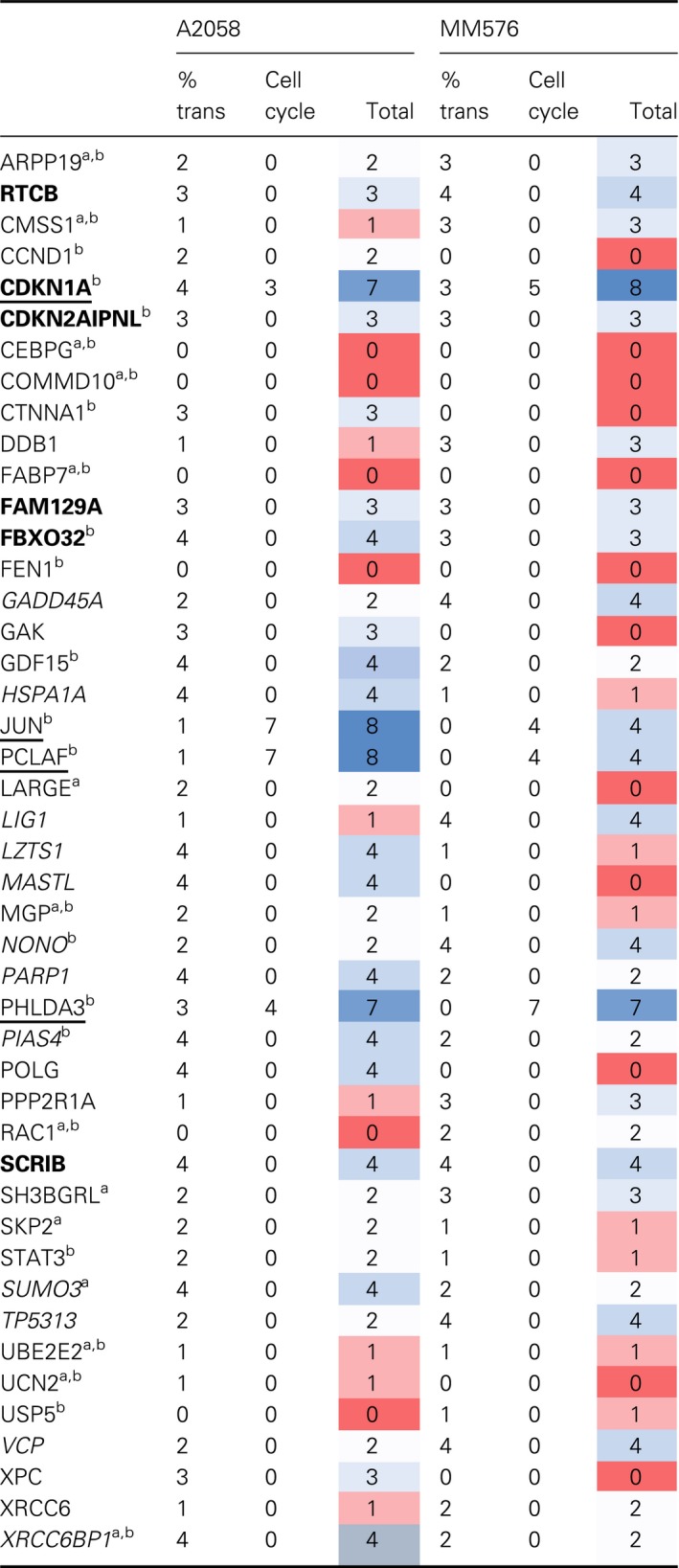
Results of overexpression screen.

Bolded genes—strongly reduced, % transduced after UVR in both cell lines. *Italicised genes*—strongly reduced, %transduced in 1 and less in the other cell line. Underlined genes—showed changes in cell cycle. The heat maps indicates lowest (red) and highest (blue values).

Downregulated in UV polysomes.

> 25% cells transduced.

### 3.3 MASTL‐ARPP‐19/ENSA‐PP2A‐B55 pathway regulates exit from the UV‐G2 checkpoint arrest

**Figure 3:**
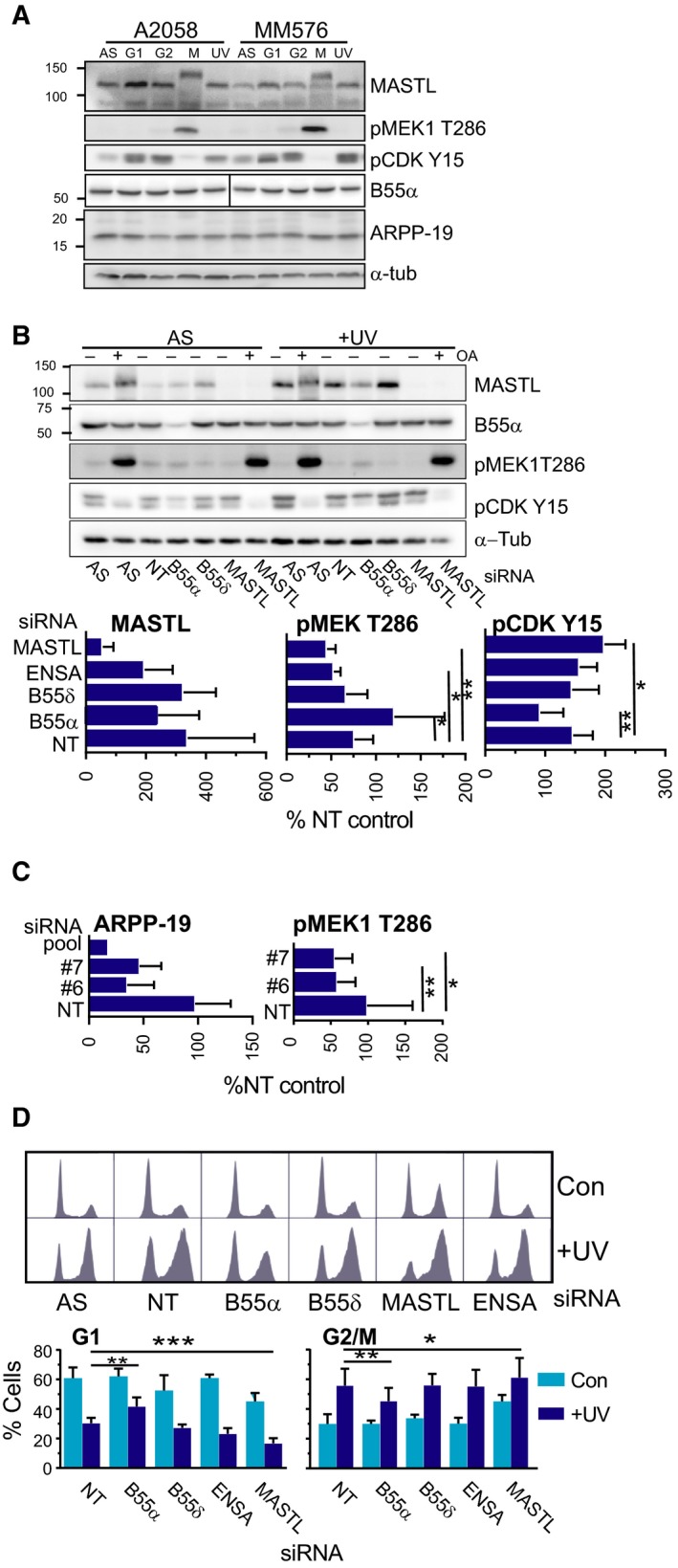
MASTL pathway is involved in recovery of the UV‐G2 checkpoint arrest. (A) Cell cycle fractions, asynchronously growing (AS) cells, and UV‐G2 checkpoint arrested cells (UV) were immunoblotted for MASTL pathway components. pMEK1 T286 is a marker of mitosis, pCDK Y15 of G2 phase, and α‐tubulin is a loading control. (B) Asynchronously growing (AS) or UV‐G2 checkpoint arrested cells (+UV) transfected with the indicated siRNA. Cells were treated without or with okadaic acid (OA) for 2 h prior to harves. The bars graphs below show quantitation of the levels of the indicated proteins or markers as a percentage of the nontargeting control in the asynchronously growing cells. (C) Quantitation of ARP19 and the mitotic marker pMEK1 T286 levels in A2058 cells transfected with two individual ARPP‐19 siRNAs (#6, #7), nontargeting (NT), and ARPP‐19 SmartPool (pool). The data are expressed as a percentage of the NT asynchronously growing cells. (D) DNA content determined by flow cytometry of A2058 cells transduced with the indicated siRNAs, either asynchronously growing (Con) or UV‐G2 checkpoint arrested (+UV). The bar graphs are the mean and SD from at least three independent experiments.

The MASTL pathway components MASTL and PPP2R2A (B55α) were identified as high‐confidence gene set from the functional analysis, and ARPP‐19 and ENSA as lower confidence gene set (Table 1). The pathway has a role in the regulation of mitotic entry by regulating the activity of PP2A‐B55 through the PP2A inhibitors ARPP‐19 and ENSA that are themselves the targets of MASTL‐dependent phosphorylation (Lorca and Castro, 2013). Both B55α and B55δ regulatory subunits of PP2A phosphatase complex have been implicated in MASTL pathway function, and although B55δ/PPP2R2D was not identified as being significantly altered in polysome mRNA loading, it was investigated here. The level of MASTL were higher in synchronized G2 phase and UV‐G2 checkpoint arrested cells than in asynchronously growing controls (Fig. 3A), and migrated faster than the hyperphosphorylated form of MASTL found in the mitotic cells (Burgess *et al.*, 2010; Fig. 3A). The B55 α‐subunit of PP2A was unchanged throughout the cell cycle and in the checkpoint response. We produced a polyclonal antibody to a peptide fragment of human ARPP‐19 (Fig. S12A,B), which showed the level of ARPP‐19 did not vary across the cell cycle or with UV irradiation (Fig. 3A). We were unable to validate the specificity of a commercially sourced ENSA antibody (Fig. S12C,D), and were not able to determine the levels of this protein.

SiRNA depletion of MASTL, the PP2A subunits B55α or B55δ, ENSA, and ARPP‐19 effectively reduced the mRNA and protein levels (Fig. 3B; Fig. S13A). The treatment with PP1/PP2A inhibitor okadaic acid demonstrated that phosphatase inhibition was sufficient to overcome the UV‐G2 checkpoint arrest and promote entry into mitosis. MASTL levels in the nontargeting (NT) siRNA‐transfected UV‐G2 phase samples were increased by 2.5‐fold over the asynchronously growing controls (Fig. 3B). In the UV‐G2 phase samples, depletion of MASTL significantly decreased the level of pMEK1 T286, a marker of mitosis (De Boer *et al.*, 2008; Fig. 3B), and significantly increased the level of the inactive Tyr15 phosphorylated CDK1 (pCDK Y15), a marker of G2 phase arrest, indicating an increase in G2 phase arrest (Fig. 3B). The depletion of B55α, but not B55δ, had the opposite effect, increasing pMEK1 T286 and decreasing pCDK Y15 levels indicating increased exit from the UV‐G2 phase checkpoint arrest into mitosis. Direct inhibition of PP1/PP2A phosphatase activity with okadaic acid promoted mitotic entry in the MASTL‐depleted cells, indicated that phosphatase inhibition was sufficient to bypass MASTL delayed mitotic entry (Fig. 3B). The ARPP‐19 and ENSA siRNA were shown to be selective for their respective genes (Fig. S13B), and ENSA depletion also produced a modest reduction in the level of pMEK1 T286 (Fig. 3B). Surprisingly, the ARPP‐19 siRNA pool produced a high level of apoptosis in the UV‐G2 phase checkpoint samples, indicated by the elevated levels of cleaved caspase 3 (Fig. S14A). Deconvolution of the pool to its four individual siRNAs revealed that siRNAs #5 and #8 were the primary contributors to the apoptosis observed, and #8 had little effect on ARPP‐19 levels, indicating that the apoptosis observed was an off‐target effect of these two siRNAs (Fig. S14A). The two selective siRNAs, #6 and #7, reduced ARPP‐19 levels by > 60%, and significantly reduced the levels of the mitotic marker pMEK1 T286, modestly mimicking the effect of MASTL depletion (Fig. 3C). The delayed exit from the UV‐G2 arrest was also detected by flow cytometry analysis of the cell cycle. UV irradiation produced a similar strong accumulation of cells with 4n DNA content in both untransfected and nontargeting siRNA‐transfected cells (Fig. 3D). MASTL and B55α depletion produced significant and antagonistic changes in the G1 and G2/M content of the UV‐G2 phase arrested population. This supports the role for B55α in regulating PP2A activity in controlling entry into mitosis from the UV‐G2 checkpoint arrest, and of MASTL by regulating ARPP‐19 and possibly ENSA to inhibit PP2A to allow exit from the UV‐G2 checkpoint arrest.

**Figure 4:**
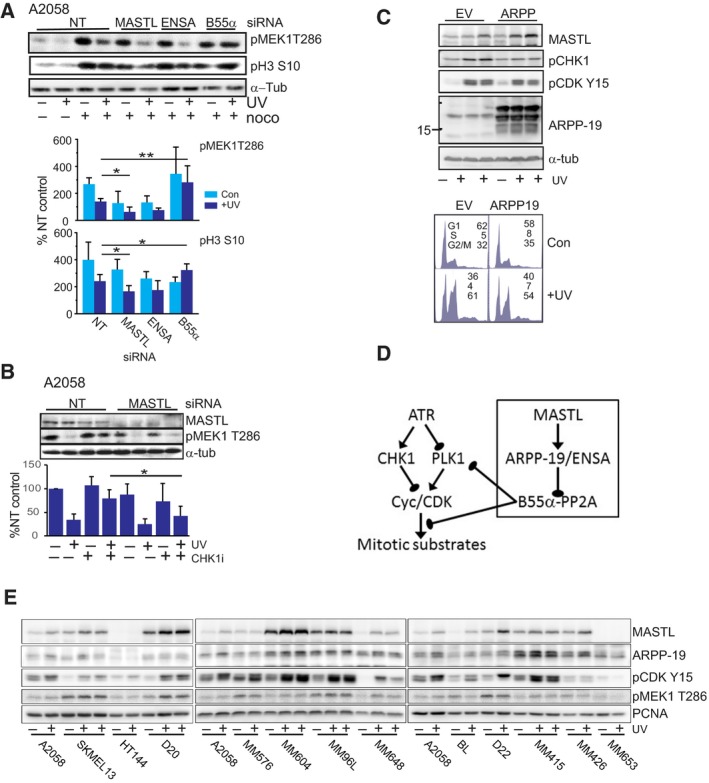
(A) Cells were transfected with the indicated siRNA and then treated as indicated. Cells were either untreated or treated with UVR. At 20 h postirradiation, nocodazole was added and incubated for further 6 h. Cells were then harvested and immunoblotted for pMEK1 T286 and phospho‐histone H3 Ser 10 (pH3 S10). α‐Tubulin was used as a loading control. The levels of these mitotic markers were quantitated and expressed relative to the nontargeted (NT) unirradiated control from 3 to 4 separate experiments. *P < 0.5, **P < 0.01. (B) Cells transfected with the indicated siRNA with or without UVB. CHK1 inhibitor was added for 2 h before harvesting. Quantitative analysis of the level of pMEK1 T286 from 3 to 4 separate experiments was performed. (C) Cells were stably transfected with human ARPP‐19 or empty vector (EV), and then treated with or without UVB and harvested after 24 h and immunoblotted for the indicated proteins or analyzed by FACS. This is representative of two experiments. The percentage of each cell cycle phase is shown. (D) Model of MASTL role in exit from the UV‐G2 checkpoint. (E) The indicated cell lines were treated with or without 150 Jm^−2^ UVB, harvested at 24 h after irradiation, and then immunoblotted for the indicated proteins. PCNA was used as a loading control. Some samples were also UV irradiated with a similar dose of UVB using a different light source.

To more clearly demonstrate the role of the MASTL pathway in controlling progression out of the UV‐G2 phase checkpoint arrest, cells exiting the arrest were trapped in mitosis with nocodazole. MASTL and ENSA depletion resulted in significant decreases in the level of the mitotic markers pMEK1 T286 and pHistone H3 Ser10, whereas B55α depletion increased the level of these markers (Fig. 4A). This effect was also observed in HeLa cells that also have a functional UV‐G2 checkpoint using pHistone H3 Ser10 as a marker of mitosis (Fig. S14B). Together, these data indicate that the MASTL‐ARPP‐19/ENSA‐B55α pathway controls exit from the UV‐G2 phase checkpoint arrest.

Entry into the UV‐G2 checkpoint arrest is controlled by CHK1 activation (Wigan *et al.*, 2012). To determine if MASTL operates upstream or downstream of CHK1, cells were depleted of MASTL, irradiated, and then at 24 h when cells were G2 phase arrested, treated with a selective CHK1 inhibitor GNE‐323 (Brooks *et al.*, 2013) and collected into nocodazole. The CHK1 inhibitor was equally efficient in promoting entry into mitosis in the nontargeting siRNA‐transfected cells, but significantly less efficient in MASTL‐depleted cells (Fig. 4B), indicating MASTL operates downstream of CHK1 to block mitotic entry. To further demonstrate this role in checkpoint exit, overexpression of the human ARPP‐19 had a modest effect on the UV‐G2 checkpoint, with decreased G2 phase arrest marker pCDK Y15 and reduced levels of activated phospho‐CHK1 Ser317 (Fig. 4C).

These data demonstrate that the MASTL pathway has a role in controlling exit from the UV‐G2 checkpoint arrest. PPP2R1A (PR65α), a low‐confidence hit from the screen (Table 1), is the invariable regulatory A subunit of the PPP2CA–B55α–PR65α trimeric complex (Glatter *et al.*, 2009). The selectivity of depletion of the B55α regulatory subunit in the effect may provide a clue as to the target of the pathway, as B55α is a specific PP2A subunit associated with regulation of PLK1 activity in exit from ATR‐dependent checkpoint arrest (Wang *et al.*, 2015). Thus, MASTL has a role in inhibiting the PPP2CA–B55α–PR65α complex at exit from the UV‐G2 checkpoint, thereby allowing it to inhibit CHK1 activity and permit activation of PLK1 to drive checkpoint recovery and entry into mitosis (Fig. 4D). MASTL also has a role in the G2 phase checkpoint arrest in response to double‐stranded DNA breaks induced by ionizing radiation (Wong *et al.*, 2016). Depletion of multiple pathway components also significantly reduced RPA focus numbers after UVR in both cell lines (Fig. 2A), suggesting that the pathway has some role in either recognizing or repairing damage. The levels of MASTL, ARPP‐19, and G2 arrest marker pCDK Y15 and mitotic marker pMEK1 T286 were assessed in a panel of melanoma cells lines 20 h following UVR when A2058 and MM576 cells were arrested at the UV‐G2 checkpoint. The basal level of MASTL and ARPP‐19 varied considerably across the cell lines with no MASTL detected in HT144 and MM653 lines, and MASTL levels increased after UVR in most cell lines, but there was little effect on ARPP‐19 levels (Fig. 4E). Lack of MASTL expression unexpectedly correlated with diminished UV‐G2 arrest indicated by the lack of accumulation of pCDK Y15 in HT144 and MM653 cells (Fig. 4E), indicating that constitutive loss of MASTL has the opposite effect of short‐term depletion. Constitutive loss of MASTL is lethal at all stages of growth (Diril *et al.*, 2016), thus loss of the UV‐G2 checkpoint may be a part of a compensation mechanism that melanoma cells without MASTL expression use to retain long‐term viability.

### 3.4 Functional annotation defines five interaction nodes in the UV‐G2 checkpoint

**Figure 5:**
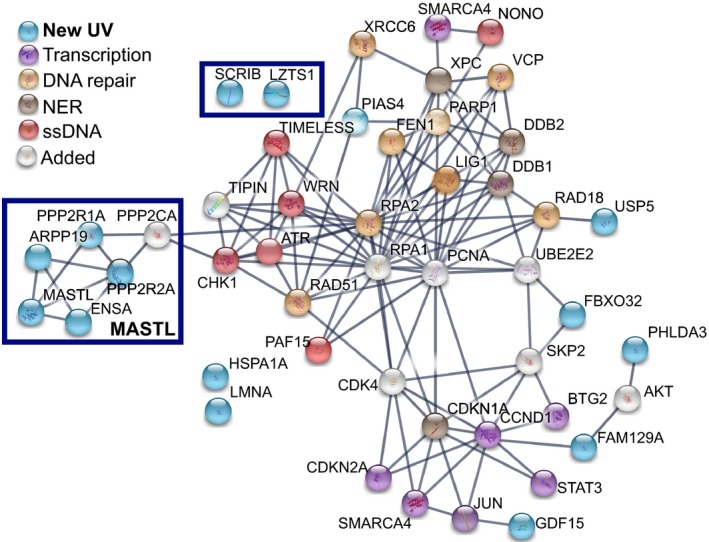
Protein interaction and functional clustering of identified hits and known UV‐G2 checkpoint components. Interaction map of the UV‐G2 checkpoint components color‐coded by functional clustering and relation to previous evidence is presented. String analysis of the functional gene list is shown. kmeans clustering with 7 clusters (more did not affect the clustering) was used. The thickness of the edges indicates the confidence, using only textmining and experimental database evidence. Average clustering coefficient was 0.59, and PPI enrichment was *P* < 10^−16^. Novel genes that influence the response are in light blue, and those in green represent the MASTL pathway. Genes in white were not identified in the screen but are known components from other studies. The blue boxes define components with no previous connection to UVR responses.

The functional analysis identified 32 genes that directly contributed to the UV‐G2 checkpoint (Table S10). When combined with known contributors to the UV‐G2 checkpoint pathway defined previously (Wigan *et al.*, 2012), the panel of 43 genes (Table S11) was used to construct an interaction map with STRING v10 (Szklarczyk *et al.*, 2015) using only highest confidence experimental and database evidence (minimum interaction score = 0.9). This demonstrated the existence of five interaction nodes (Fig. 5A): an ssDNA response node centered on ATR, CHK1, and TIMELESS; a node covering GG‐NER consisting of XPC, DDB1, and DDB2; DNA repair genes BRCA1, XRCC6, RAD51, RAD18, PARP1, FEN1, and CDKN1A/p21; a transcriptional node containing BTG2, CCND1, CDKN2A, STAT3, JUN, and SMARCA4; and the MASTL, ENSA, ARPP‐19, PPP2R2A, and PPP2R1A pathway.

#### 3.4.1 ssDNA response and NER nodes

A number of global genomic‐NER (GG‐NER) components such as XPC and DDB1 were identified in the screen, indicating that this pathway is one mechanism for repairing the UV‐induced lesions during the UV‐G2 checkpoint arrest. DDB1 is complexed with CUL4‐RBX1 (CLR4) E3 ubiquitin ligase and utilizes DDB2 as its substrate recognition subunit. The CLR4^DDB2^ complex can detect UV‐induced DNA lesions which releases inhibition of the CLR4^DDB2^ E3 ligase activity to ubiquitinate its substrates, DDB2, nucleosomal histones, and XPC (Zhu and Wani, 2017). VCP is a ubiquitin‐specific segregase that binds and removes ubiquitinated DDB2 and XPC from UV‐induced lesions, which is essential for their repair (Sugasawa, 2016; Zhu and Wani, 2017). Depletion of each of DDB1, XPC, and VCP genes reduced viability after UVR treatment, strongly delayed progression through S/G2 phase, and significantly reduced RPA focus and cell numbers in both cell lines (Fig. 2; Fig. S7). XPC depletion results in a G2 phase arrest after UVR exposure (van Oosten *et al.*, 2000; Quinet *et al.*, 2014). This suggests an inability of replication forks to progress past the unrepaired lesions, possibly due to the presence of the DDB2 and XPC containing pre‐excision NER complex. XRCC6/KU70 can negatively regulate the DDB2 E3 ligase complex in response to UVR (Takedachi *et al.*, 2010), and its depletion modestly increased the number of RPA foci (Fig. 2). PARP1 has a role in regulating DDB22 and XPC in GG‐NER (Robu *et al.*, 2017). UBE2B/RAD6 is the E2 ubiquitin ligase component with the E3 ligase RAD18 that monoubiquitinates PCNA to promote POLH‐dependent TLS (Watanabe *et al.*, 2004). It was downregulated at both total mRNA level and polysome loading in the UV‐G2 response, although we have previously found there is an increase in monoubiquitinated PCNA in UV‐G2 checkpoint arrested cells (Wigan *et al.*, 2012). Together, the data suggest a key role for GG‐NER in repairing the unrepaired UV‐induced lesions during S phase and inhibiting WRN‐dependent lesion bypass by the replication fork that produces the single‐stranded DNA gap opposite the unrepaired lesion that is recognized by RPA and triggers the UV‐G2 checkpoint.

One of the strongest effects observed with the functional screening was with the depletion of TIMELESS. TIMELESS interacts with TIPIN and RPA2 to regulate CHK1 activation and the S phase checkpoint response to UVR, which is an immediate response to UV‐induced DNA damage in S phase (Unsal‐Kacmaz *et al.*, 2007). Depletion of TIMELESS overcame the replication arrest induced by UVR. The very strong accumulation of S and G2 phase cells and the strong accumulation of RPA foci (Fig. 2; Fig. S7) indicate that TIMELESS also has a role in the signaling of unrepaired UV‐induced lesions detected as cells progress into S phase. Interestingly, loss of TIMELESS would be expected to block CHK1 activation and allow continued cell cycle progression, but there is clearly a delay in progression through S and G2/M phases. The strongly increased number of RPA foci suggests that depletion of TIMELESS could be involved in the repair of NER ssDNA gaps through the regulation of replicative polymerase activity (Cho *et al.*, 2013). CDKN1A is reported to be degraded in response to low‐dose UVR, and this is required for repair by GG‐NER and TLS as an early response to UV‐induced DNA damage (Cazzalini *et al.*, 2010; Mansilla *et al.*, 2013). Previous work has shown that CDKN1A protein levels are decreased in UV‐G2 arrested A2058 cells (Gabrielli *et al.*, 1997), but it was strongly upregulated in both total and polysome‐loaded mRNA (Table S6). This may indicate that a dynamic turnover of the protein is required for repair. Depletion reduced the numbers of RPA foci and reduced the cell cycle delay (Fig. 2; Fig. S7), whereas overexpression increased the delay after UVR exposure (Fig. S10), suggesting it has a role in initiating the repair of UV‐induced lesions. Together, these data point to the role of GG‐NER in the UV‐G2 repair response.

#### 3.4.2 DNA repair node

NONO is an RNA and DNA binding protein that acts a molecular scaffold for a range of molecular processes including RNA splicing, transcription, and DSB repair and in response to UVR (Alfano *et al.*, 2016; Knott *et al.*, 2016). It can also bind poly(ADP‐ribose) that is deposited by PARP1 at the sites of DNA damage (Krietsch *et al.*, 2012). In response to UVR, it regulates RAD9 loading onto damaged chromatin (Alfano *et al.*, 2016). Here, depletion of NONO delayed progression through the UV‐G2 phase checkpoint and strongly increased RPA foci numbers (Fig. 2; Fig. S7), which is evidence for the fact that NONO depletion inhibits the possible repair of ssDNA gaps opposite the UV‐induced lesions.

BTG2 is a growth‐suppressive gene that can regulate *CCND1* expression (Tirone, 2001). Its expression is increased in response to DNA damage (Winkler, 2010), and it has a role in regulating the G2 phase checkpoint arrest in response to DSBs (Rouault *et al.*, 1996), in part by regulating the MRE11 activity (Choi *et al.*, 2012). No role in response to UVR has been reported. Here we find that depletion of BTG2 delayed progression through the UV‐G2 checkpoint, but its effects on lesion detection or repair are unclear, with significant although modest changes in RPA foci numbers in both directions (Fig. 2; Fig. S7).

FEN1 and LIG1 are normally involved in maturation of Okazaki fragments in lagging strand DNA replication (Dovrat *et al.*, 2014), and may play a similar role in repair of lesions on the lagging strand in the UV‐G2 checkpoint. Interestingly, depletion of FEN1 reduced, and depletion of LIG1 increased the RPA foci numbers significantly in both cell lines (Fig. 2B). Increased RPA foci are likely to indicate replication forks encountering unrepaired lesions and stalling, although LIG1 depletion had no effect on either cell number or cell cycle progression (Figs S6 and S7), suggesting that its depletion might uncouple repair and cell cycle checkpoint signaling. FEN1 depletion reduced cell numbers and increased the S/G2 fraction of cells in unirradiated controls, suggesting that these cells are progressing more slowly through the cell cycle which may account for the reduced RPA foci.

USP5 is a ubiquitin peptidase that has a role in homologous recombination repair, where its loading onto sites of double‐stranded DNA breaks is RAD18‐dependent (Nakajima *et al.*, 2014). PIAS4 is a SUMO E3 ligase that has a role in DSB repair in removing RPA from resected ssDNA to allow repair (Galanty *et al.*, 2012). Its depletion appears to slow down normal S phase and progression after UVR exposure (Fig. S7). Depletion of both modestly increased RPA foci numbers (Fig. 2B) supporting their role in homologous recombination repair of the ssDNA gaps.

#### 3.4.3 Transcriptional node

SMARCA4/BRG1 has a role in regulating transcriptional responses to UVR, including *CDKN1A, GADD45A*, *IL8*, *FAM44A*, *IGFBP3*, *S100A2,* and *CCNG1* (Zhang *et al.*, 2014). SMARCA4 associated with STAT3 is also reported to regulate the *CDKN1A* expression (Giraud *et al.*, 2004). SMARCA4 containing SWI/SNF complex can also remodel chromatin‐bound UV‐induced lesions detected by CRL4^DDB2^ which in turn ubiquitinates the chromatin histones (Palomera‐Sanchez and Zurita, 2011; Zhao *et al.*, 2009).

C‐JUN has a well‐established role in response to high doses of UVR (Waster *et al.*, 2014; Wisdom *et al.*, 1999). Its depletion had a modest but significant effect of reducing RPA foci numbers and delayed progression through S phase after UVR (Fig. 2; Fig. S7), and conversely its overexpression appeared to increase the rate of exit from the arrest (Fig. S10). These effects suggest an unappreciated role for C‐JUN in regulating gene expression in the UV‐G2 response to suberythemal UVR. GDF15 is an MITF target gene and has been reported to promote proliferation and increase tumor growth through JUN‐dependent increased CCND1 expression (Boyle *et al.*, 2009; Jin *et al.*, 2012). The effect of depletion of GDF‐15 was similar to CCND1 depletion, supporting the fact that GDF‐15 is connected to the transcriptional node.

#### 3.4.4 Other genes and pathways

PHLDA3 and FAM129A/NIBAN may be involved in an AKT‐dependent protein translational control in response to UVR. There is an increase in AKT and mTOR activation and downstream translational regulators in response to UVR (Bermudez *et al.*, 2015). PHLDA3 is an inhibitor of AKT activation by blocking its plasma membrane binding (Kawase *et al.*, 2009). FAM129A/NIBAN is a confirmed AKT substrate in response to UVR (Ji *et al.*, 2012), and has been reported to regulate protein translation by regulating mTOR targets 4E‐BP1 and S6K (Sun *et al.*, 2007). Whether this is a general effect or selective for a subset of mRNA is unknown, although the very high proportion of transcripts that are regulated at only the translational level, indicated by the differential loading onto polysomes (Fig. 1B), does suggest that translational control is the major component of the response to UVR. Interestingly, depletion of both these genes significantly increased RPA foci numbers and delayed progression through the UV‐G2 checkpoint (Fig. 2; Fig. S7) possibly by decreasing translation of the many translationally regulated genes involved in the UV‐G2 response.

LZTS1 and SCRIB were two previously unsuspected contributors to the UV‐G2 checkpoint response. There is evidence that LZTS1 has a role in G2/M progression, potentially stabilizing the CDK1/Cyclin B complex as well as influencing microtubule dynamics in mitosis (Vecchione *et al.*, 2007). LZTS1 mRNA loading on polysomes was increased by > 2 fold, and siRNA depletion had a modest effect on RPA foci formation and cell number. It slowed progression into or through S phase at 24 h and accumulation in G2/M compartment at 40 h after UVR (Fig. 2; Fig. S7). Overexpression also appeared to affect G2/M progression at 40 h post‐UVR (Fig. S10). The G2/M phase accumulation may be related to its known G2/M roles, but the S phase effect is novel and will require further investigation. SCRIB is a regulator of cell polarity influencing ERK activity (Nagasaka *et al.*, 2010). SiRNA depletion of SCRIB had modest effects in reducing cell numbers after irradiation, and reduced progression into and through S/G2/M phases after UVR (Figs S6 and S10). Overexpression strongly reduced cell numbers after irradiation in both cell lines (Fig. S9). SCRIB overexpression can inhibit ERK activation/activity (Dow *et al.*, 2008) which would be sufficient to slow cell cycle progression after UVR (Giles *et al.*, 2012).

Booth HSPA1A and LMNA have multiple interaction partners in this gene set, as does PARP1 (Table S10), although how these interactions influence the UV‐G2 checkpoint is unknown. SDHA depletion has modest effects on RPA foci number, in both cases delaying the reduction in foci at 40 h after UVR suggesting delayed repair (Fig. 2; Fig. S7).

### 3.5 Dysregulated expression of UV‐G2 checkpoint pathway genes correlates with increased UV signature mutation load in melanomas

The very high levels of UV signature mutations (USM; Signature 7 mutations) that are observed in melanomas are the direct outcome of unrepaired UV‐induced lesions (Alexandrov *et al.*, 2013), suggesting that there must be defects in the repair of these lesions. Initial analysis of The Cancer Genome Analysis (TCGA) melanoma dataset showed that upregulated expression of the panel of 43 UV‐G2 checkpoint pathway genes identified (Table S11) was a common feature in melanomas (*z* score > 3; Fig. S15). Interestingly, *SCRIB*, *ENSA,* and *XRCC6* were overexpressed in > 10% of melanomas.

To assess whether dysregulated expression of components of the UV‐G2 checkpoint pathway potentially contributes to the accumulation of the USMs in melanomas, we have analyzed the relationship between the dysregulated expression of the 43 genes identified and USMs in the TCGA melanoma dataset using the Pathifier algorithm (Drier *et al.*, 2013). After samples were filtered, there were complete datasets for 352 melanomas. Using this approach, there was a weak but significant correlation between the pathway dysregulation score (PDS) and USM (Spearman cor = 0.25, *n* = 352, *P* = 3.4 × 10^−6^; Fig. S16).

**Figure 6:**
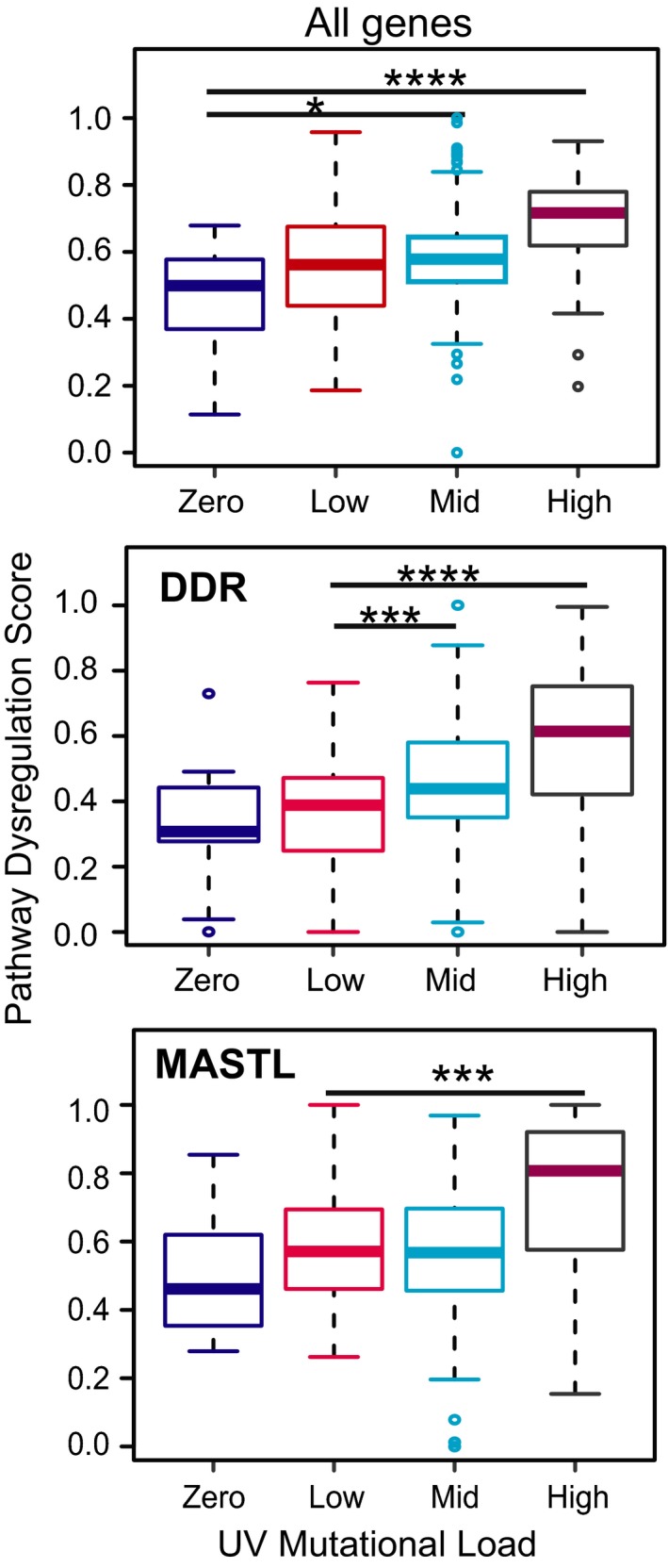
Dysregulated expression of the UV‐G2 checkpoint pathway genes correlates with increased UV signature mutation load. Box and whisker plot of pathway dysregulation scores for tumors from the TGCA melanoma dataset categorized into zero, low, medium, or high UV signature mutation load. Top plot shows all genes identified in Table S11, middle plot the DNA damage response (DDR) genes of this set, and bottom plot shows the MASTL genes identified as significant in Table S14. *P < 0.05, ***<0.001, ****<0.0005.

The samples were subgrouped into high‐, mid‐, and low‐USM load as described previously (D'Arcy *et al.*, 2019). The median PDS for the high‐ and mid‐mutation samples was significantly higher than the zero‐mutation controls (Fig. 6; Table S12). Dividing the gene list into cell cycle checkpoint‐related and DNA damage repair‐related genes (Table S11), most of the effect was from the DNA repair genes, where both mid‐ and high‐USM groups were significantly higher compared to zero‐ and low‐USM groups (Fig. 6; Table S13). Another study using Pathifier to assess homologous recombination repair (HR) pathway dysregulation demonstrated a PDS (HR score) < 0.4 to be HR proficient and > 0.5 HR deficient (Liu *et al.*, 2016). The median PDS in the mid‐ (0.58) and high (0.72)‐UV‐mutational load groups suggests that dysregulation of the UV‐G2 checkpoint pathway is a contributor to the increased USM load found in the mid‐ and high‐load melanomas. Analysis of the major individual contributing genes revealed that three of the MASTL pathway genes (PPP2R1A, MASTL, and ARPP‐19) as most significant, particularly when comparing low to high USM load tumors, suggesting that dysregulation of this pathway is a major contributor to high USM loads (Tables S13 and S14, Fig. 6).

## 4 Conclusion

This study has identified multiple pathways that contribute to the UV‐G2 checkpoint response. (a) A high proportion of the components of the response are regulated translationally and this appears to be through an AKT‐dependent mechanism involving PHLDA3 and FAM129A. (b) XPC‐mediated GG‐NER is the likely mechanism to repair the UV‐induced lesions that are detected by this postreplication repair mechanism. (c) The contribution of BTG2, PAIS4, and USP5, and previously demonstrated contributions of BRCA1 and RAD18 provide strong evidence that the ssDNA gaps recognized by RPA are repaired by homologous recombination repair. (d) The MASTL‐ARPP‐19‐PP2A pathway has a role in progression out of the UV‐G2 checkpoint arrest that is independent of CHK1. Importantly, dysregulated expression of the repair components of the UV‐G2 checkpoint pathway appears to be significantly associated with the UV signature mutation load in melanomas, as is the MASTL pathway, indicating that loss of normal function of the UV‐G2 checkpoint response is likely to be a major contributor to the increase in UV mutations that are a major driver of melanoma.

## Conflict of interest

The authors declare no conflict of interest.

## Author contributions

SP and BG contributed to conception and design of research; SP, AP, WF, and DL‐O performed experiments and acquired the data; MS, MK, AB, NC, and RAS provided technical advice and support; SP, WF, DL‐O, DS, MMH, NC, and BG analyzed the data; ND, NM, and K‐A LC performed the bioinformatic analysis; BG organized and drafted the manuscript; ND, NM, K‐A LC, MS, MK, AB, NC, and RAS provided critical input for the manuscript preparation and important intellectual input.

## Supporting information


**Fig. S1.** FACS of DNA content of synchronized populations.
**Fig. S2.** Immunoblots of synchronized populations.
**Fig. S3.** Cell counts from siRNA controls.
**Fig. S4.** Immunofluorescence staining for RPA foci.
**Fig. S5.** Scheme for siRNA transfection and lentivirus transduction gene overexpression functional experiments.
**Fig. S6.** Cell counts from siRNA screen.
**Fig. S7.** DNA content from siRNA screen.
**Fig. S8.** Plots of fold change of RPA foci numbers against p value.
**Fig. S9.** Transduction rate for lentiviral transductions.
**Fig. S10.** DNA content of transduced cells.
**Fig. S11.** V5 tag immunostaining of transduced cells.
**Fig. S12.** Validation of the human ARPP19 antibody.
**Fig. S13.** Quantitative real‐time RT‐PCR of MASTL pathway components.
**Fig. S14.** Deconvolution of ARPP‐19 siRNAs.
**Fig. S15.** Expression level of UV‐G2 checkpoint components from TCGA melanoma data.
**Fig. S16.** Scatter plot showing the correlation between the samples pathway dysregulation score (PDS) and the number of UV signature mutations (Signature 7).

**Table S1. **Total RNA asynchronous vs UV.
**Table S2. **Total RNA asynchronous vs G2.
**Table S3. **Polysome RNA asynchornus vs UV.
**Table S4. **Polysome RNA asynchornus vs G2.
**Table S5. **RNA‐seq of polysome RNA.
**Table S6. **Final gene list of UV‐G2 regulated polysome transcripts.
**Table S7. **Final gene list of UV‐G2 regulated polysome transcripts, siRNA and overexpression list.
**Table S8. **SiRNA and overexpression scoring scheme.
**Table S9. **Results of overexpression screen.
**Table S10. **Functional gene interactions.
**Table S11. **Validated damage repair and checkpoint gene list.
**Table S12. **Pathway dysregulation score for all genes vs mutational load.
**Table S13. **Pathway dysregulation score for pathway subsets vs mutational load.
**Table S14. **Individual gene dysregulation score vs mutational load.
