# Supplementary material for: Multiple interaction nodes define the postreplication repair response to UV‐induced DNA damage that is defective in melanomas and correlated with UV signature mutation load
Source: Mol Oncol. 2019 Dec 19;14(1):22–41. doi: 10.1002/1878-0261.12601 (PMC6944116; doi:10.1002/1878-0261.12601)

Supplementary Figures:

Figure S1: DNA content analysis by flow cytometry of Cell cycle fractions from asynchronously growing (Async), thymidine synchronised G1 and G2 phase cells, nocodazole arrested mitotic cells (M), and UV-G2 checkpoint arrested cells. The cells were from the melanoma cell lines A2058 and MM576.

Figure S2: Immunoblots of cell cycle fractions for the indicated proteins from the same samples as analysed in Supplementary Figure S1. PCNA is a loading control.

Figure S3: **A.** Cell counts for untransfected (cells only) and cells transfected with non-targeting (NT) or positive control PLK1 siRNAs for both A2058 and MM576 cells. Three time points were assessed, mock irradiated and irradiated with UVR then allowed to recover for 24 and 40 h. The data are mean and SD from six replicates. **B.** Viability of the same experiment A was measured by resazurin assay. The data are expressed a percentage of the untransfected, mock irradiated cells. C. Timeline of the siRNA experiment. The arrowheads indicate where the cells were fixed for analysis.

Figure S4: A2058 cells, either control or 24 h after UVR were immunostained for RPA2 and DNA. RPA foci are detected in the UVR treated cells.

Figure S5: **A**. Scheme for the siRNA transfection and lentivirus transduction gene over-expression functional experiments. After transfection/transduction, cells were allowed to recover for the indicated time then a sample collected as the unirradiated control, and at 24

and 40 h after irradiation. B. The DNA content of non-targeting DNA transfected cells at the indicated harvest times. The 24 h time point captures the UV-G2 checkpoint arrest and 40 h captures recovery from the arrest.

Figure S6: Cell counts for the A2058 and MM576 cells, untransfected, non-targeting (NT) and PLK1 siRNA transfection controls, and transfected with siRNAs targeting UV-G2 checkpoint candidate genes. Three timepoints were assessed, mock irradiated controls, and cells fixed 24 and 40 h after UVR. The blue line is the number cell in the MM576 control (M) and the black line is the control for the A2058 (A).

Figure S7: DNA content profiles determined by high content imaging of the cells transfected in Supplementary Figure S6. SiRNA that influence the cell cycle responses are bolded.

Figure S8: Plots of the fold change in RPA foci numbers per cell for siRNA depletion of each UV-G2 checkpoint candidate gene relative to the non-targeting control, against the adjusted p value (Tukey HSD test) for each timepoint. Each cell lines is shown independently.

Figure S9: The transduction rate calculated from the V5 positive cell staining for each UV-G2 checkpoint candidate gene over-expressed in A2058 and MM576 cells using lentiviral transduction.

Figure S10: DNA content (X-axis fluorescence intensity scale, log transformed, arbitrary units) density plot for A2058 and MM576 cells transduced with lentivirus expressing the indicated genes. Only genes where >500 V5 tag positive cells were identified at each time point are shown (Poly, mock transduced cells).

Figure S11: Immunostaining of A2058 cells transduced with lentivrus expressing the indicated V5 epitope tagged proteins. RPA2 and DNA staining for each is also shown. **A, B** show proteins that do not change with UVR. **C** shows over-expressed genes whose cellular localisation changed following UVR. **D.** Cellular localisation of MASTL pathway components from the over-expression screen.

Figure S12: A. Affinity purified rabbit polyclonal antibody was produced against the N-terminal peptide of human ARPP19. This was used to immunoblot a lysate of A2058 cells, without or with pore-incubation with the immunogen peptide. The same lysate was also immunoprecipitated with either the ARPP19 antibody or a non-immune rabbit IgG, then immunoblotted with ARPP19 antibody. The level of depletion of ARPP19 mRNA was >80% (Supplementary Figure S2).

B. Lysates of the indicated cell lines were transfected with non-targeting (NT) or indicated siRNA for 24 h then immunoblotted with the ARPP19 antibody.

C. A2058 cells transfected with either non-targeting (NT) or ENSA siRNA for 24 h were immunoblotted with the ENSA antibody. The level of depletion of ENSA mRNA was >80% (Supplementary Figure S2).

D. A2058 cells either transfected with the indicated siRNAs, or immunoprecipitates (IP) from panel A and the input lysate (IN), were immunoblotted with the ENSA antibody.

Figure S13: Quantitative real-time reverse transcription was performed to assess the mRNA levels of (A) MASTL, ARPP19, ENSA, B55α and B55δ and probe specificity was evaluated for (B) ARPP19 and (C) ENSA. Transcript levels were normal against the indicated house-keeping transcripts.

Figure S14: (A) A2058 cells were transfected with the individual siRNAs and pooled (P) siRNA targeting ARPP-19 or non-targeting siRNA for 24 h then immunoblotted for the indicated proteins. (B) HeLa cells were treated as the A2058 cells in Figure 6A, and the mitotic marker pH3 was used to assess progression into mitosis. This is representative of two separate experiments.

Figure S15: Genetic defect and mRNA expression level analysis of the indicated genes (PPP2R2A synonym for B55α) from the TCGA Skin Cutaneous Melanoma data set. For expression level a z-score of 3 was used as the cut-off.

Figure S16: Scatter plot showing the correlation between the samples pathway dysregulation score (PDS) and the number of UV signature mutations (Signature 7). Majority of samples are >0.4 PDS, indicating widespread dysfunction in DNA damage (DDR) and Cell cycle control (CCC) genes. The red lines show the cut-offs for low, mid and high mutation loads.

Pavey et al Supplementary Figure S1

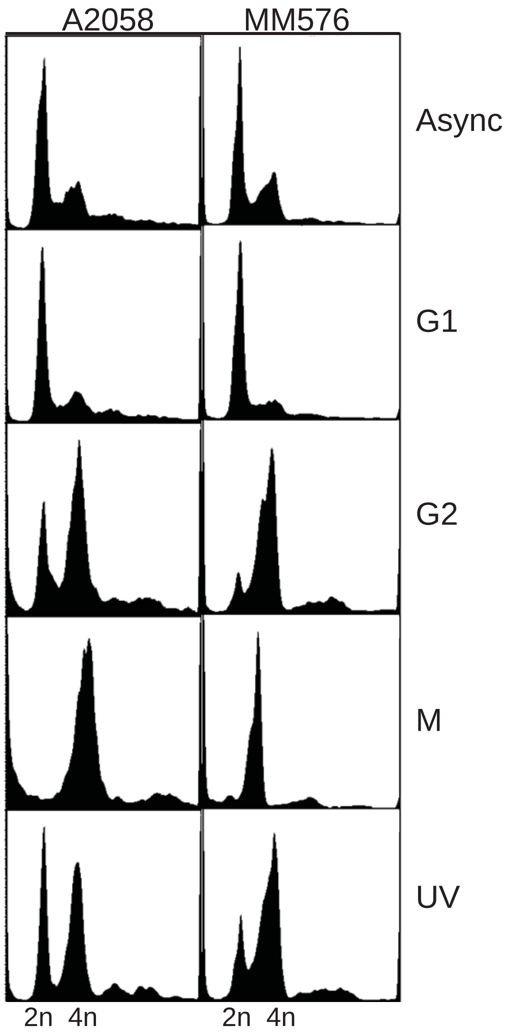

Pavey et al Supplementary Figure S2

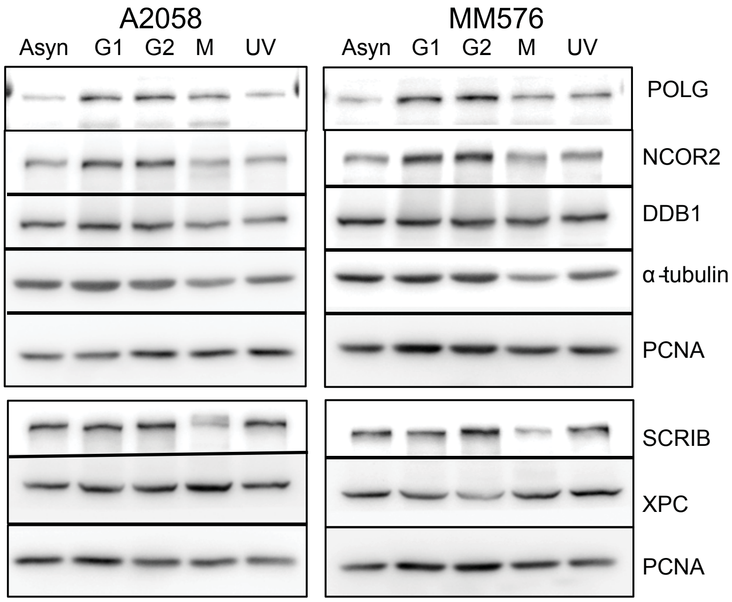

Pavey et al Supplemetary Figure S3

A

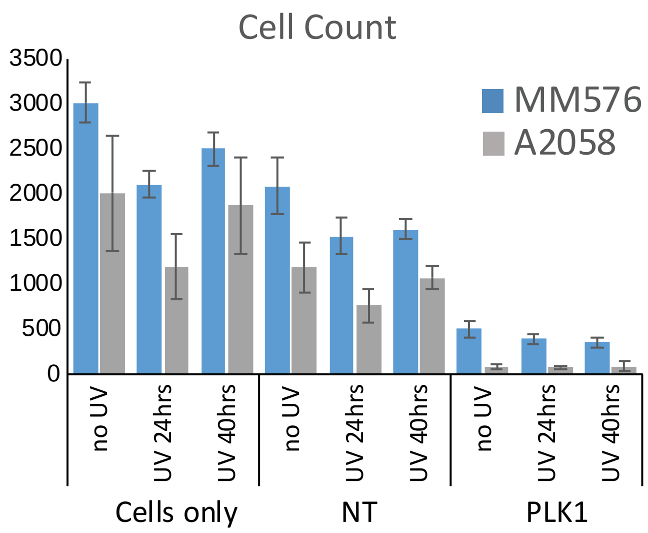

B

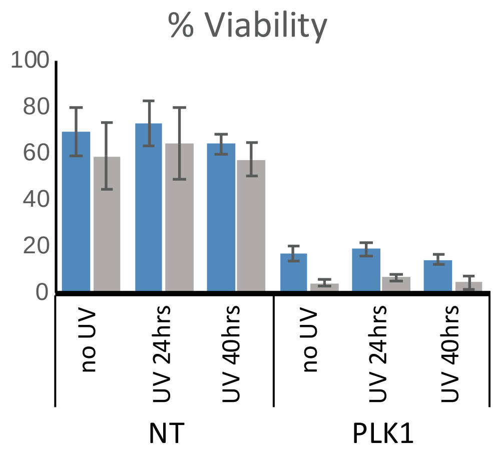

Pavey et al Supplemetary Figure S4

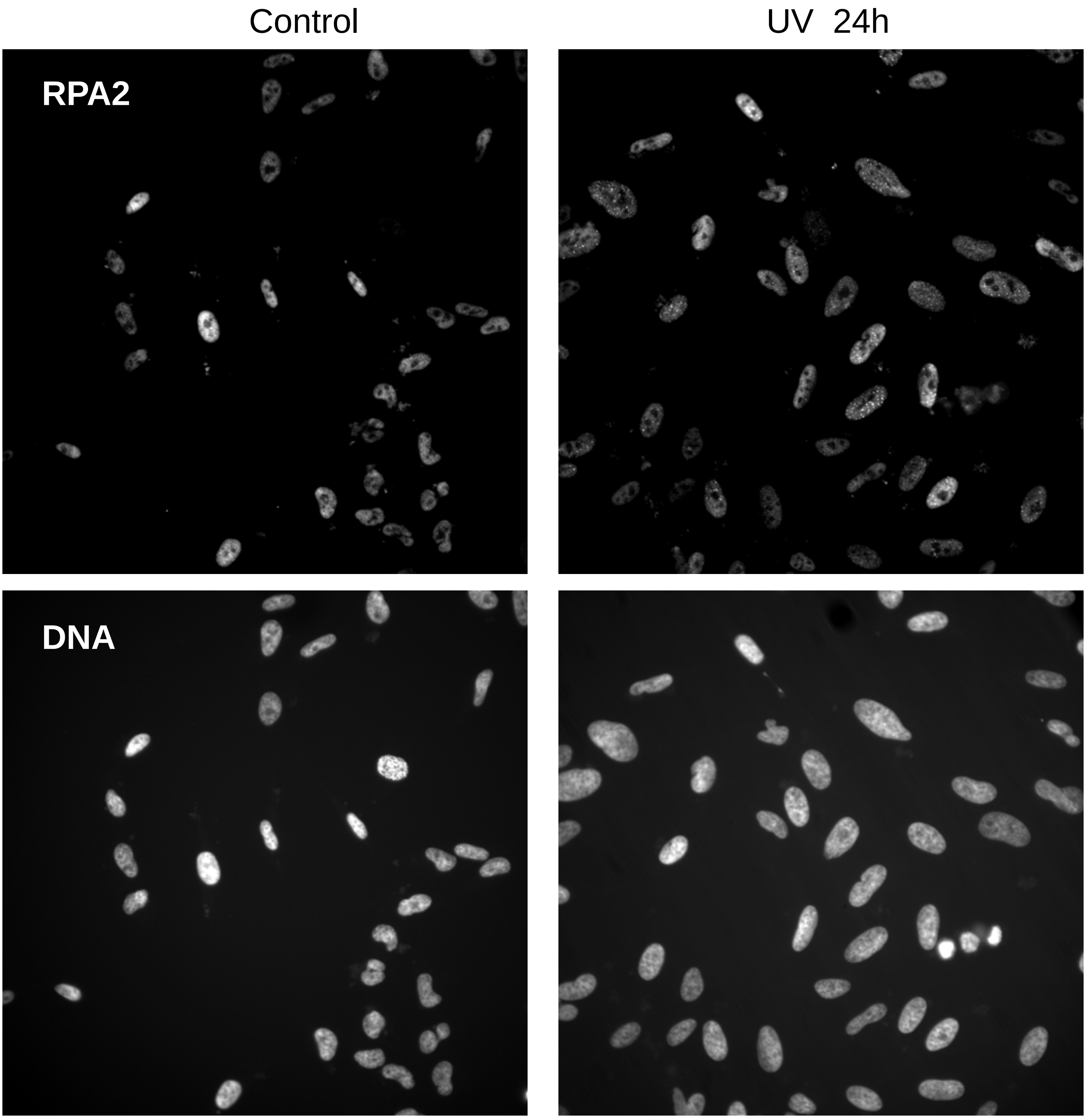

Pavey et al Supplemetary Figure S5

A

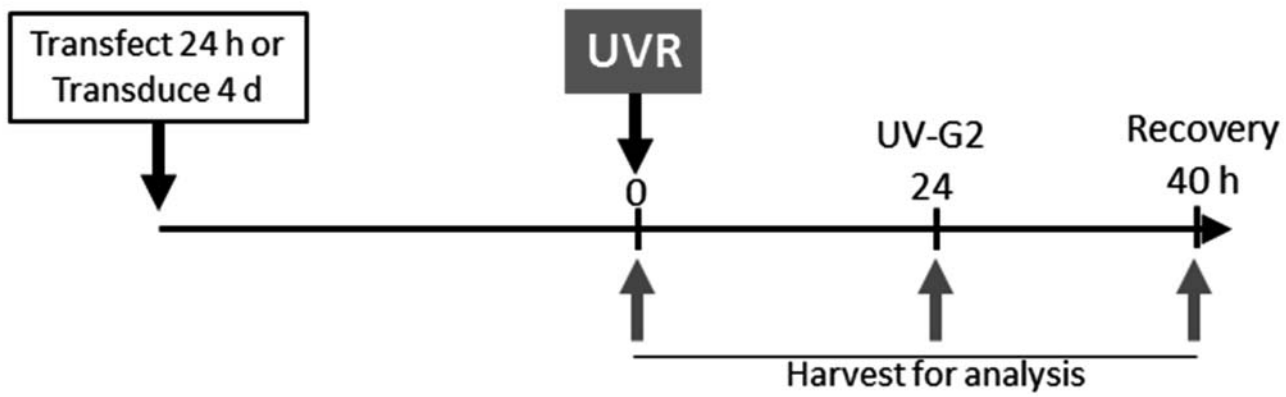

B

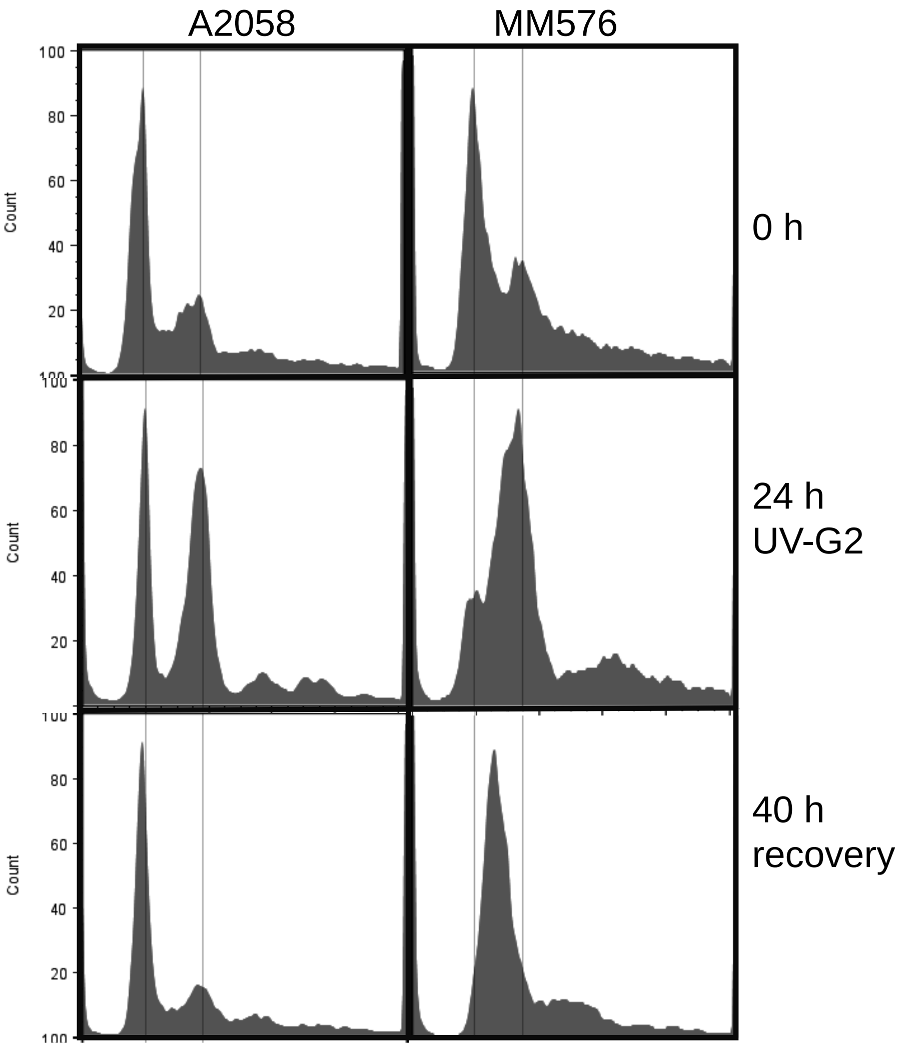

Pavey et al Supplemetary Figure S6

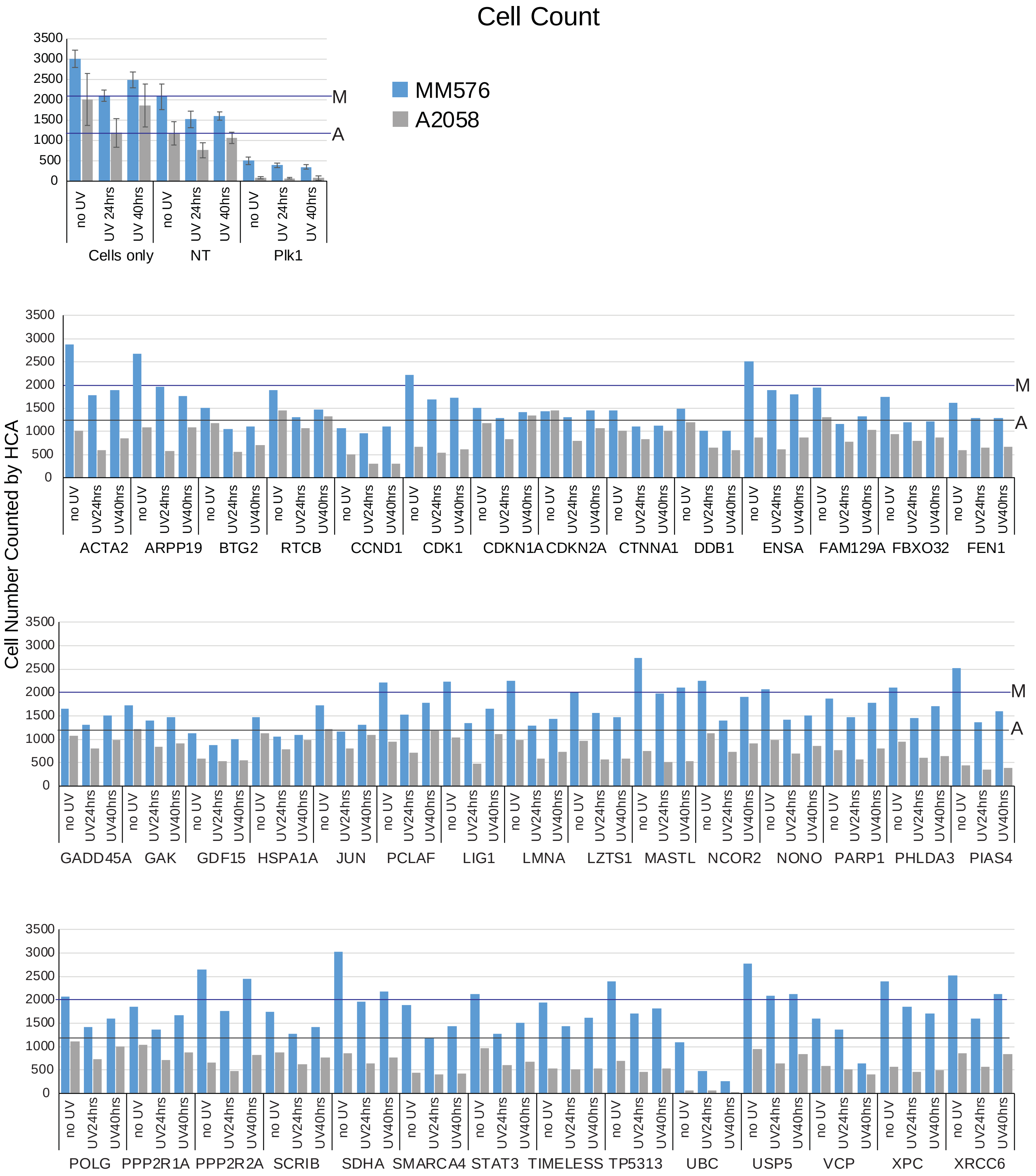

Pavey et al Supplemetary Figure S7

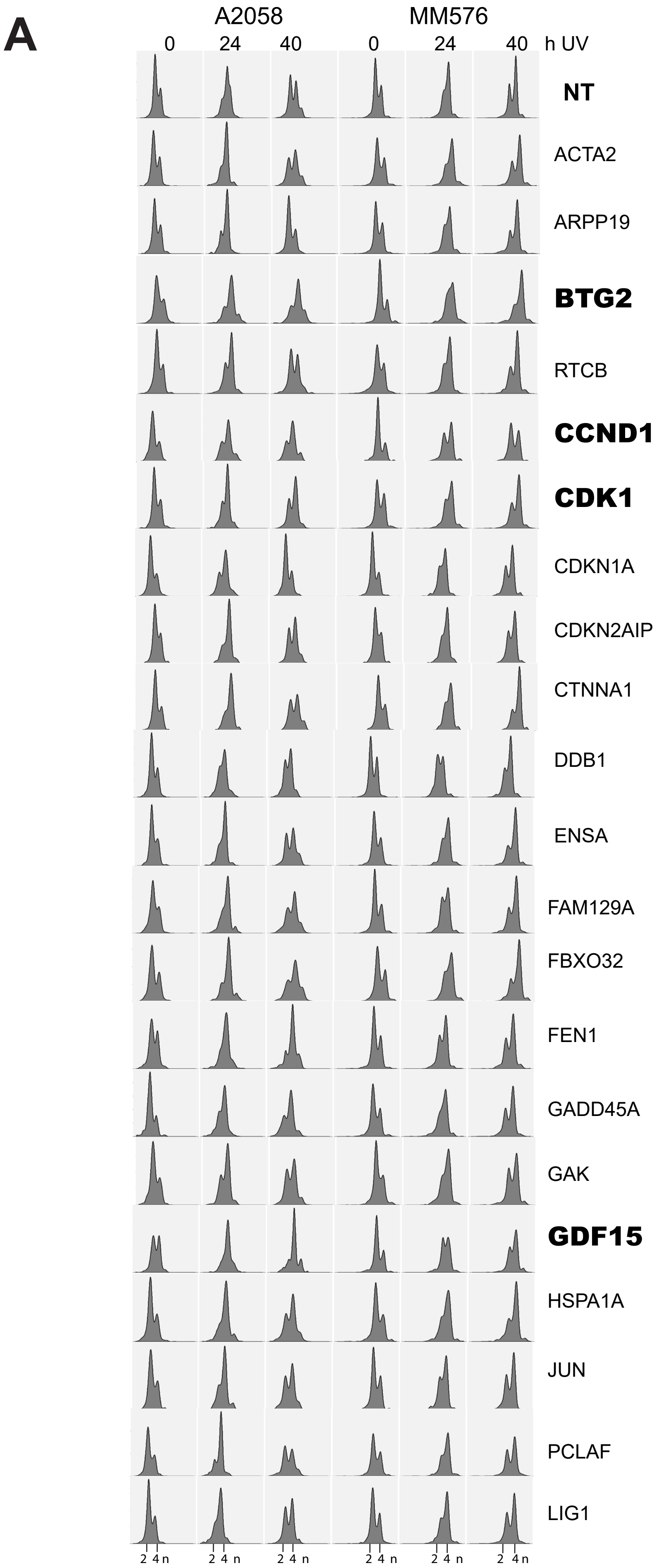

Pavey et al Supplemetary Figure S7

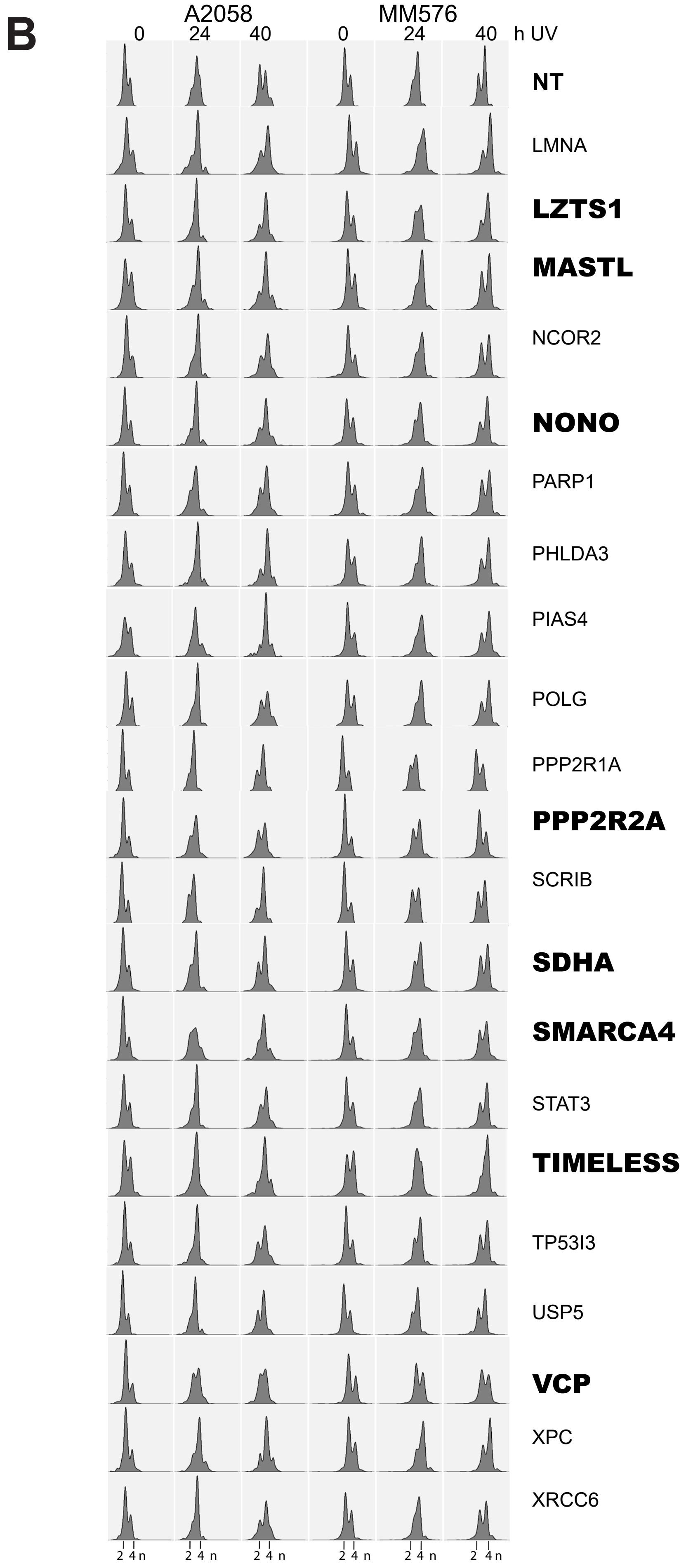

Pavey et al Supplemetary Figure S8

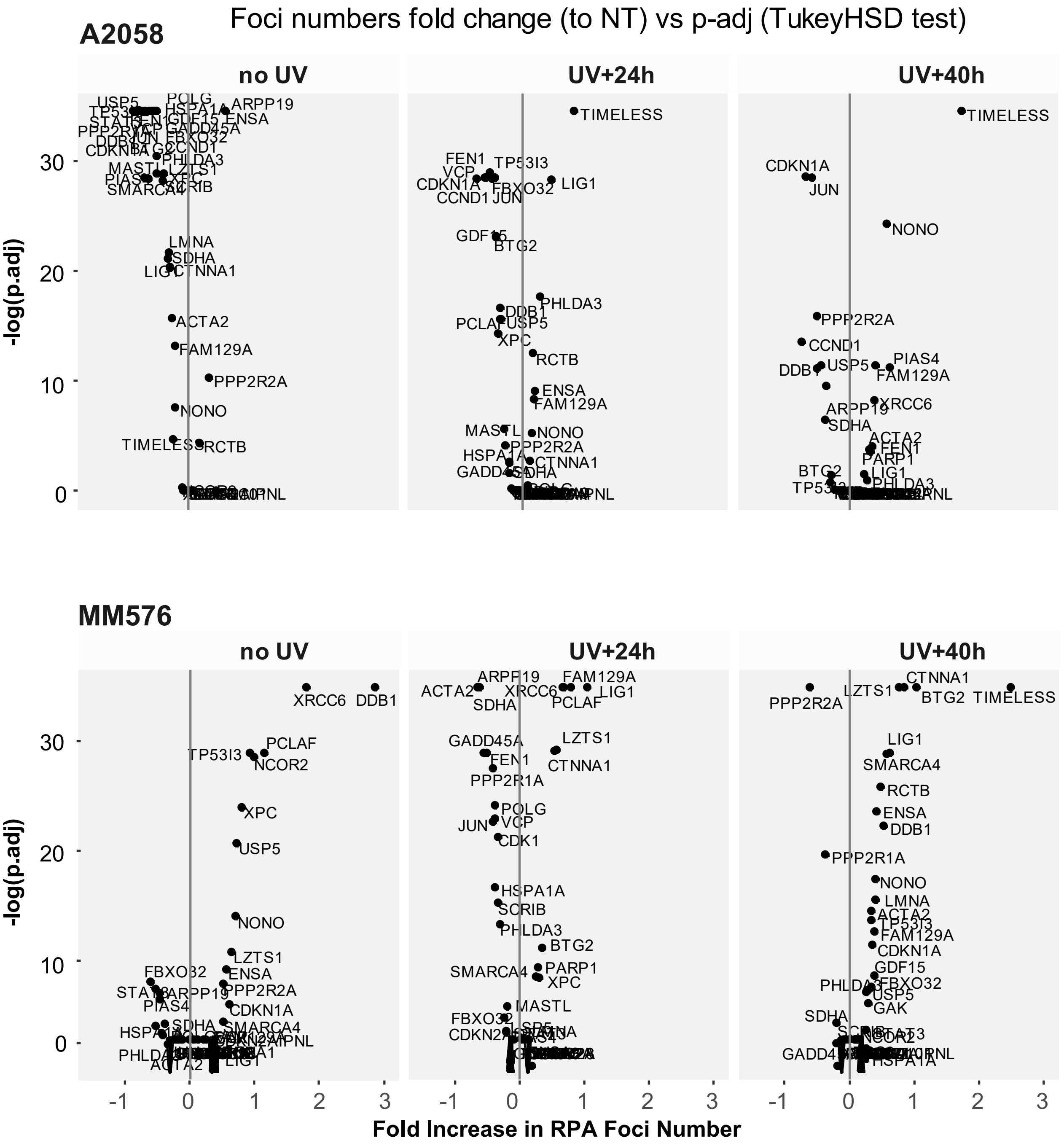

Pavey et al Supplemetary Figure S9

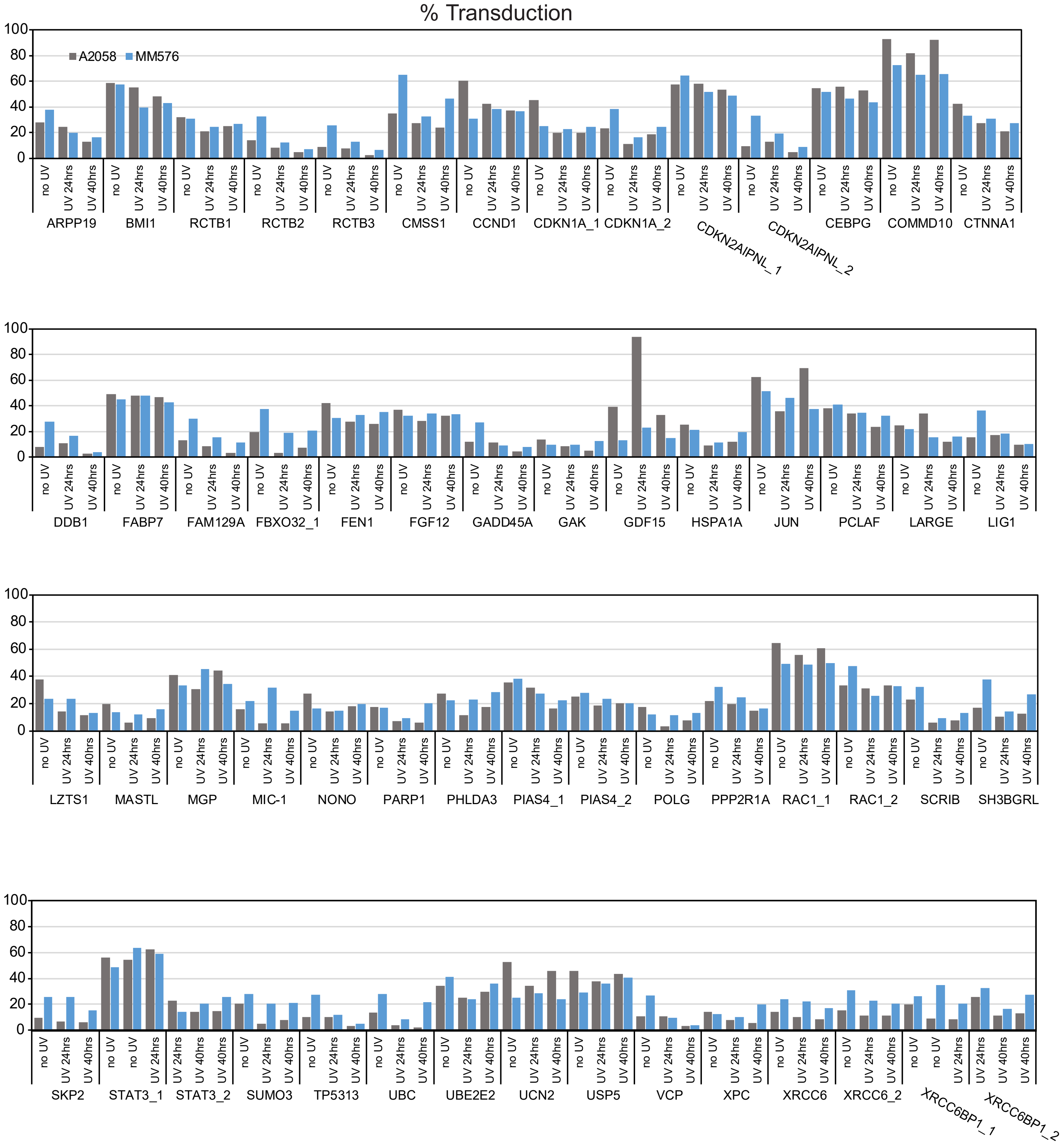

Pavey et al Supplementary Figure S10

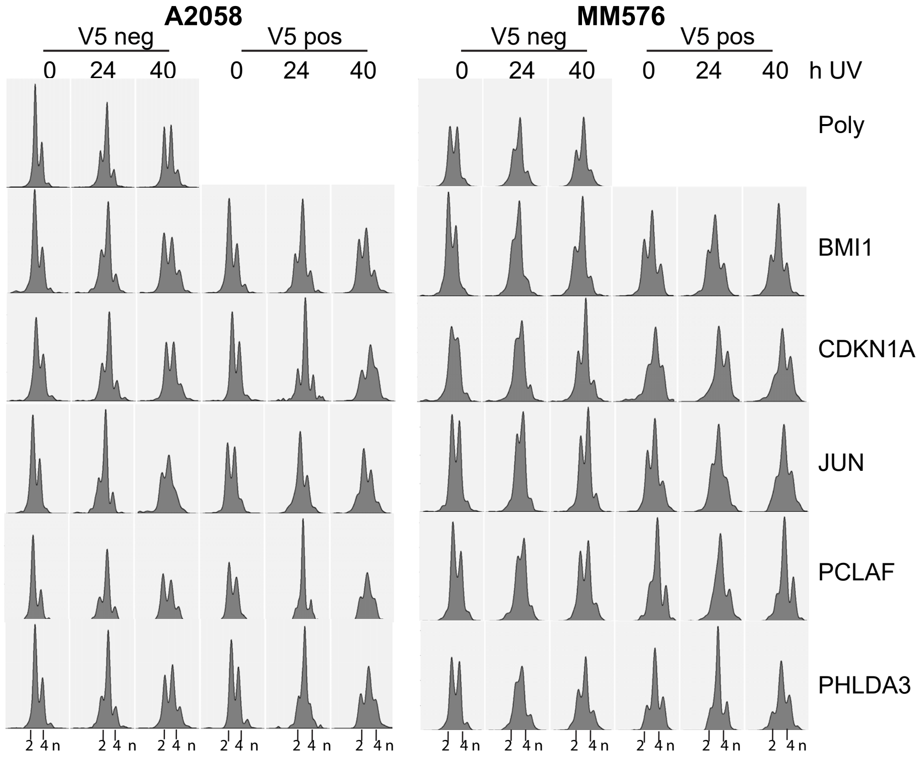

Pavey et al Supplementary Figure S11

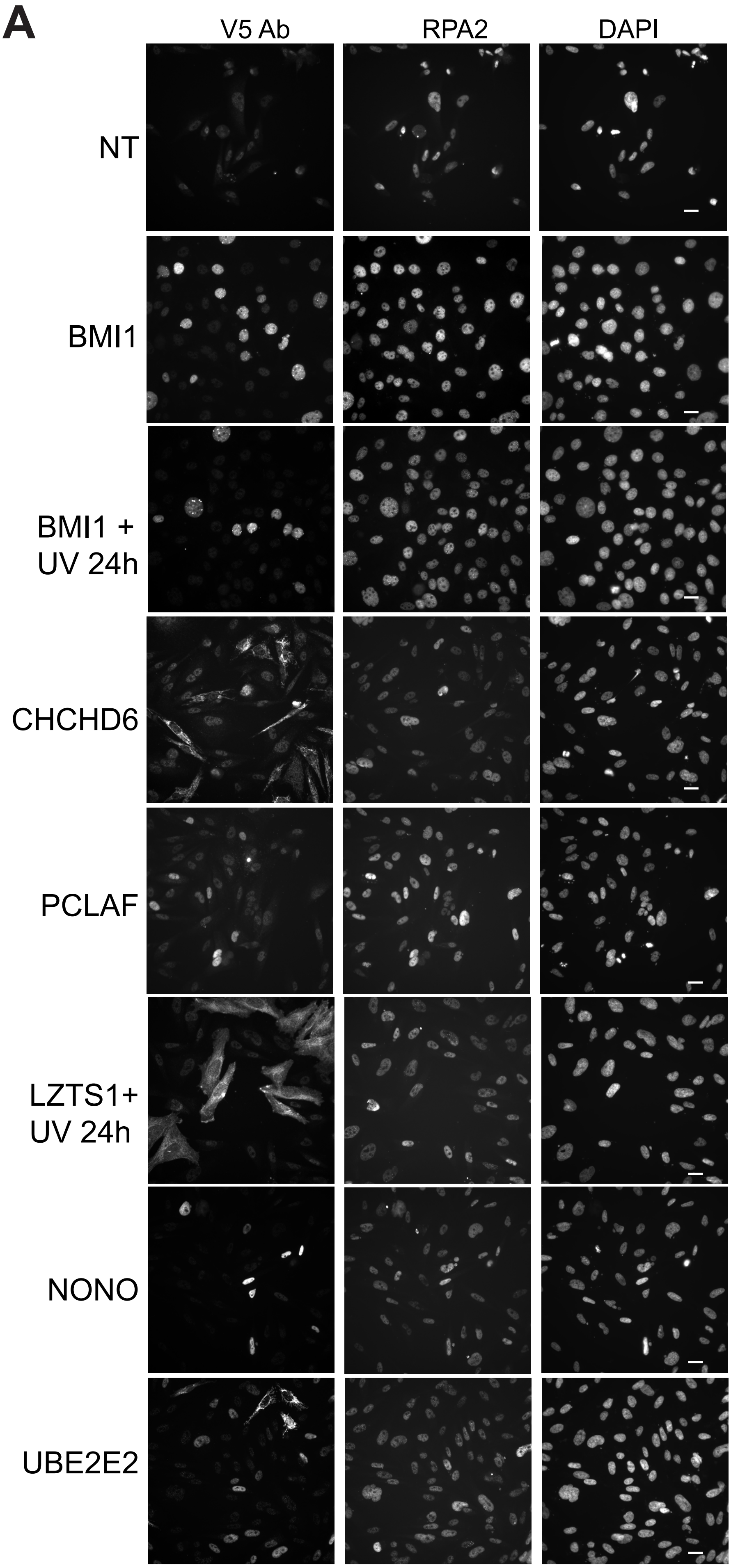

Pavey et al Supplementary Figure S11

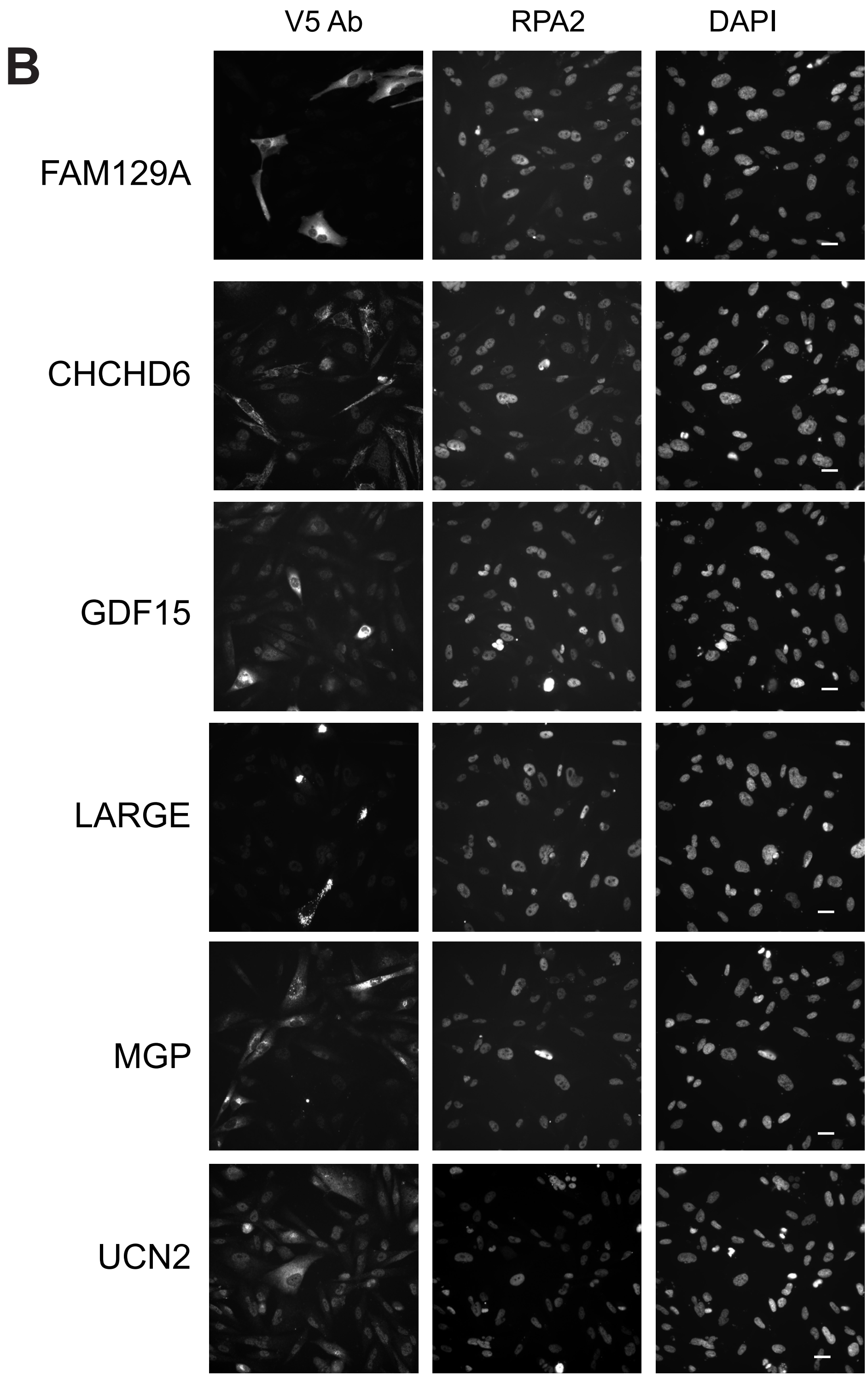

Pavey et al Supplementary Figure S11

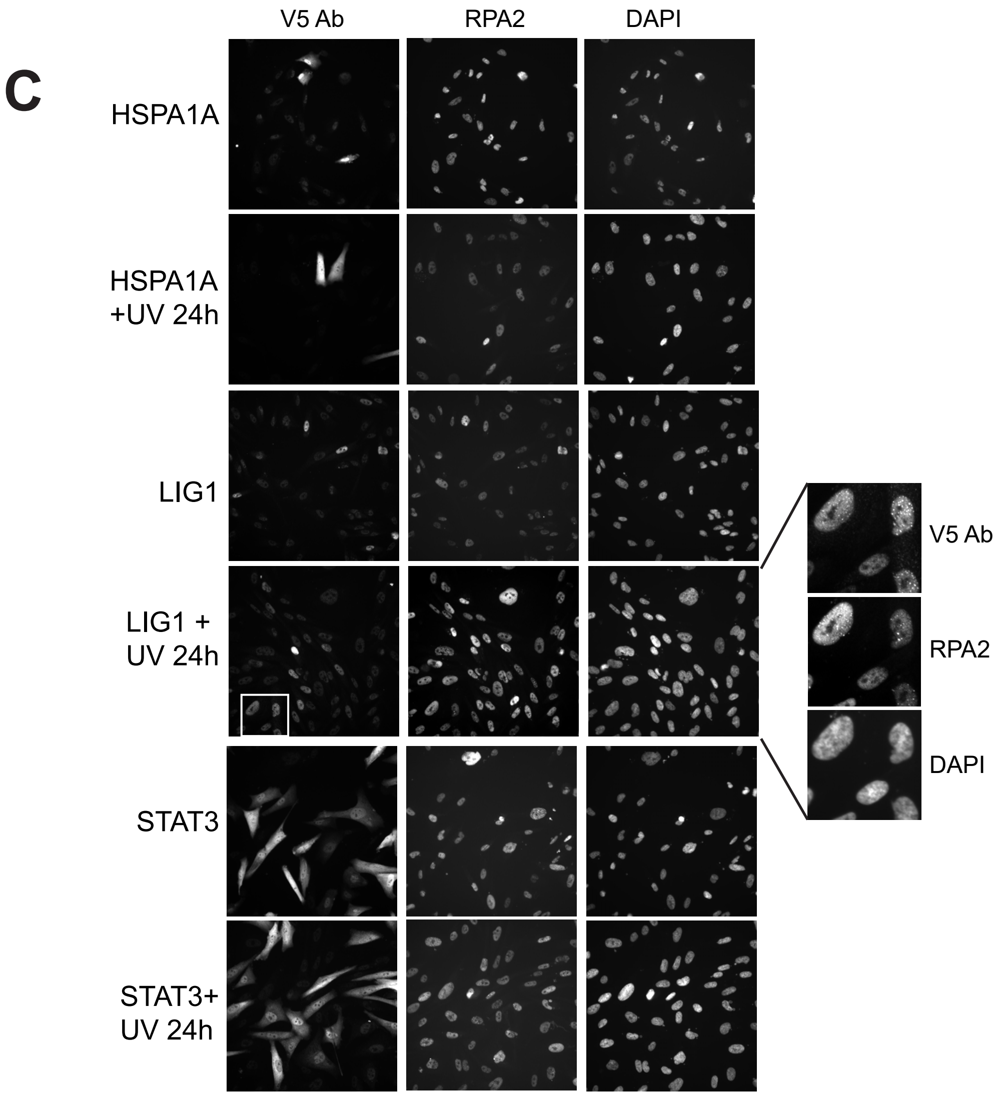

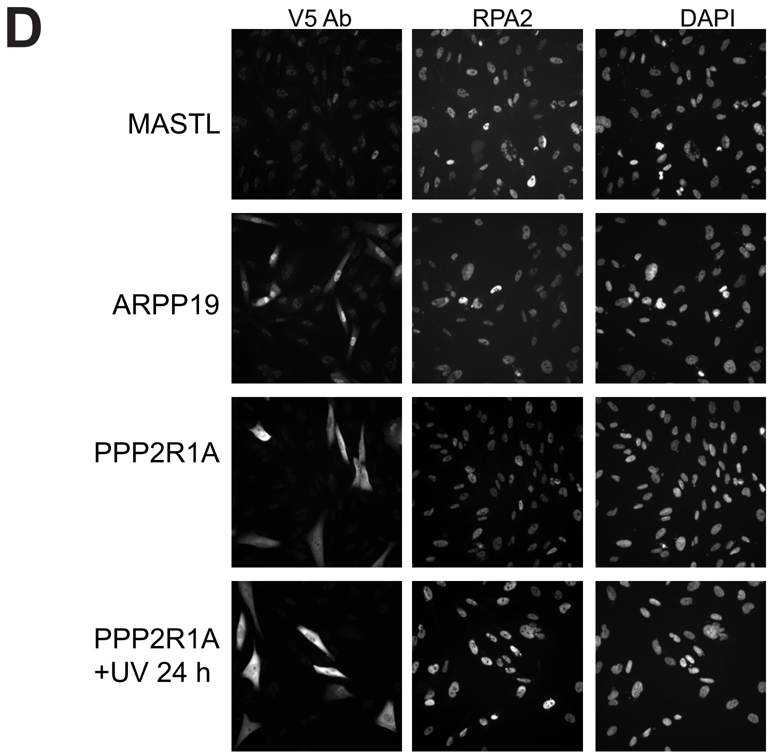

Pavey et al Supplementary Figure S12

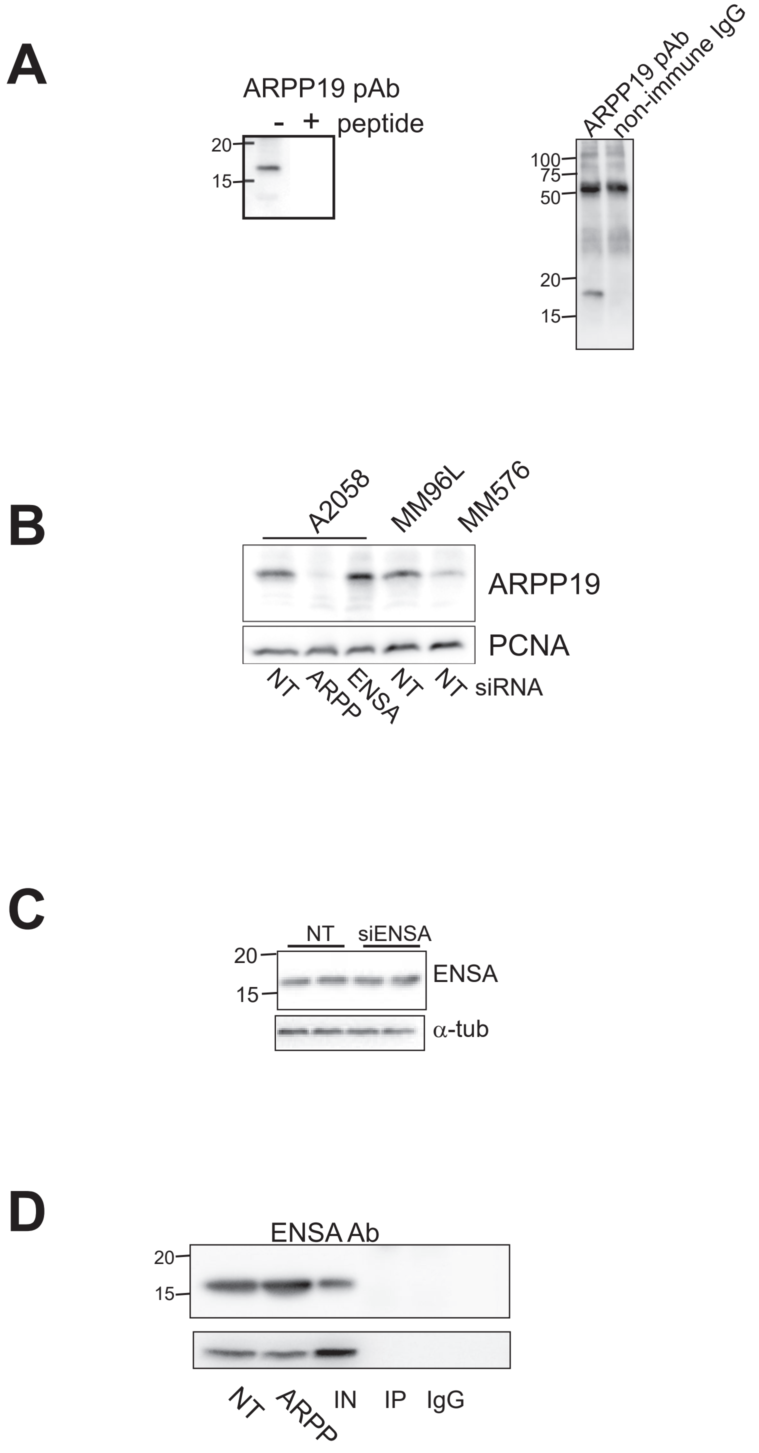

Pavey et al Supplementary Figure S13

A

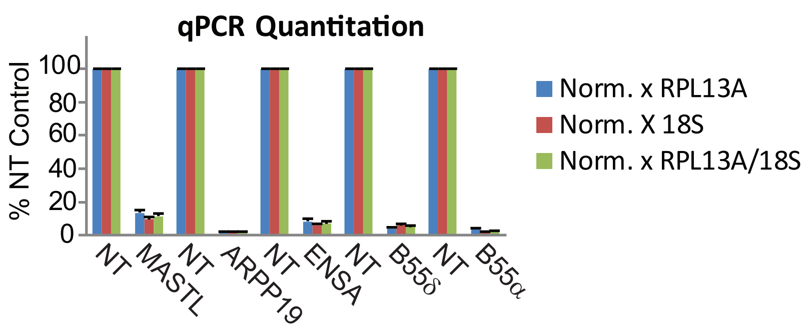

B

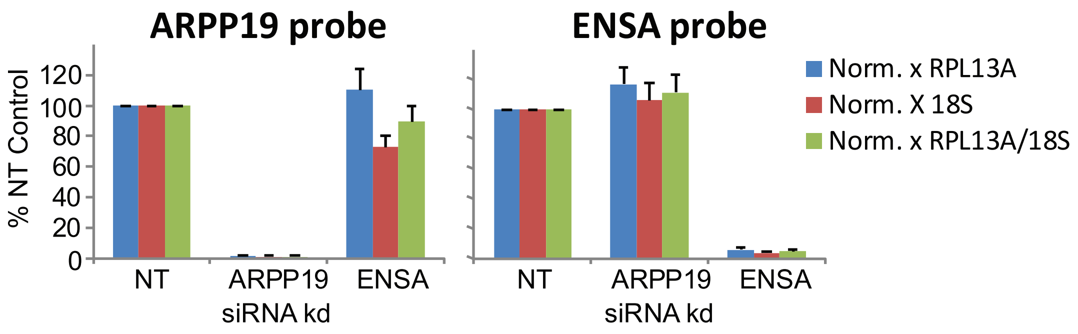

Pavey et al Supplementary Figure S14

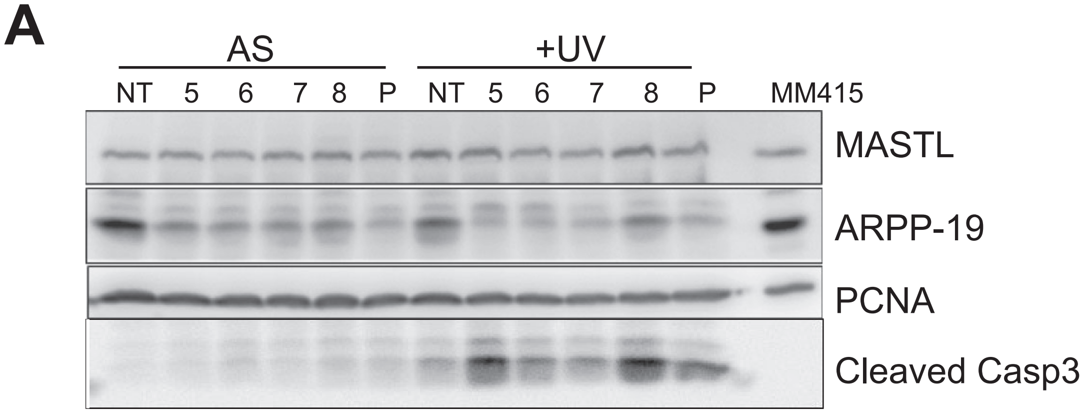

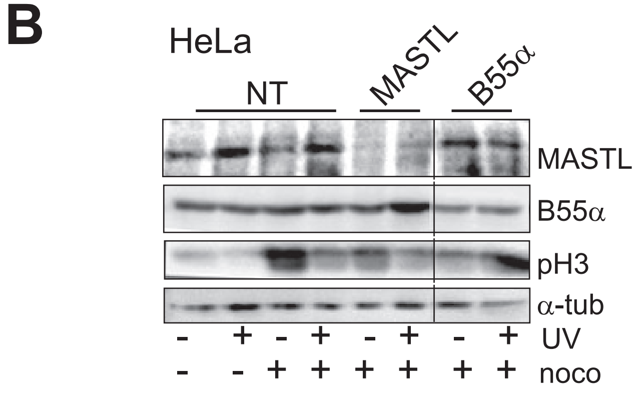

Pavey et al Supplementary Figure S15

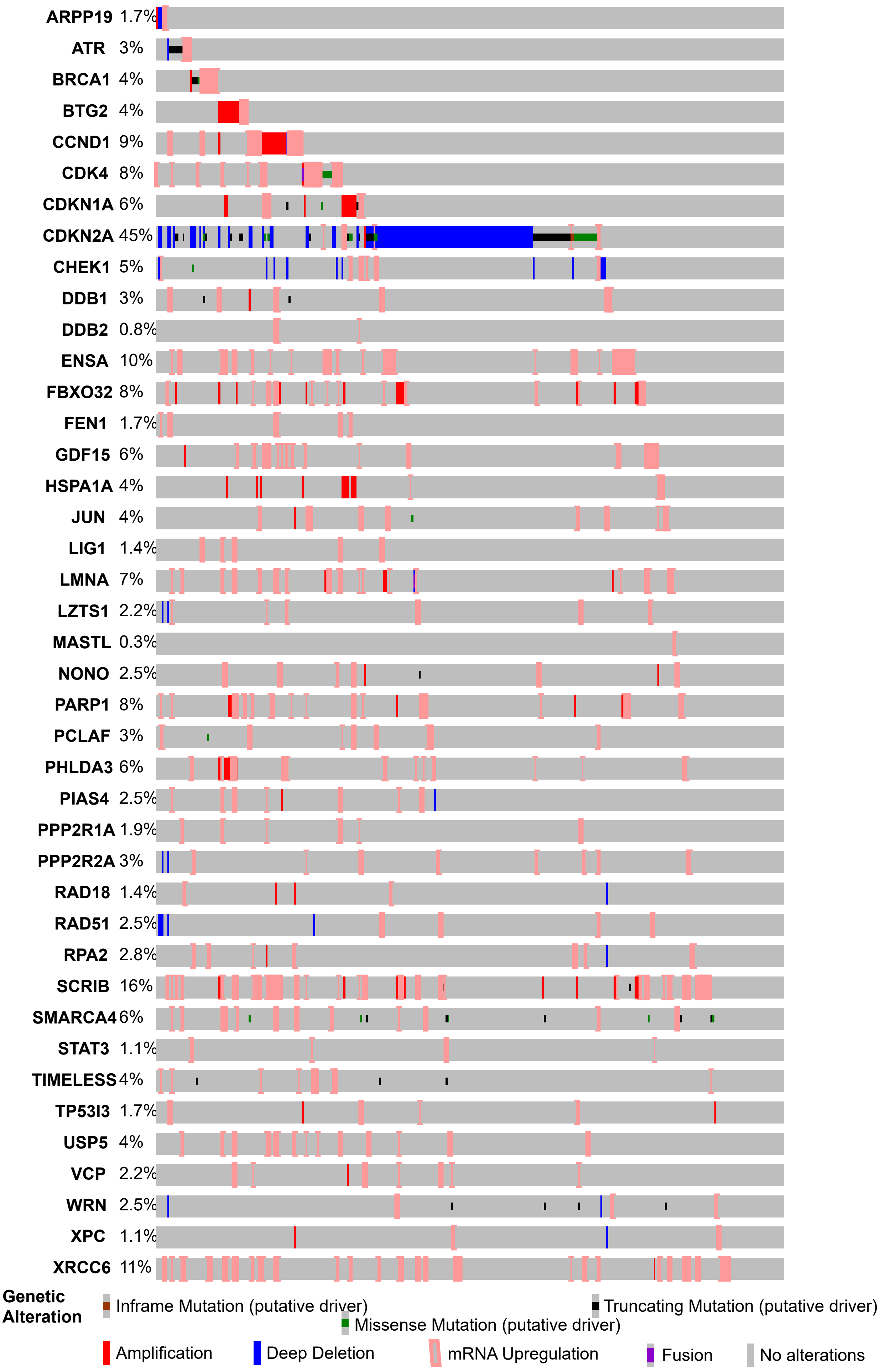

Altered in 332 (91%) of 363 sequenced cases/patients (363 total) TCGA, Pan Cancer ATLAS; z score 3

Pavey et al Supplementary Figure S16

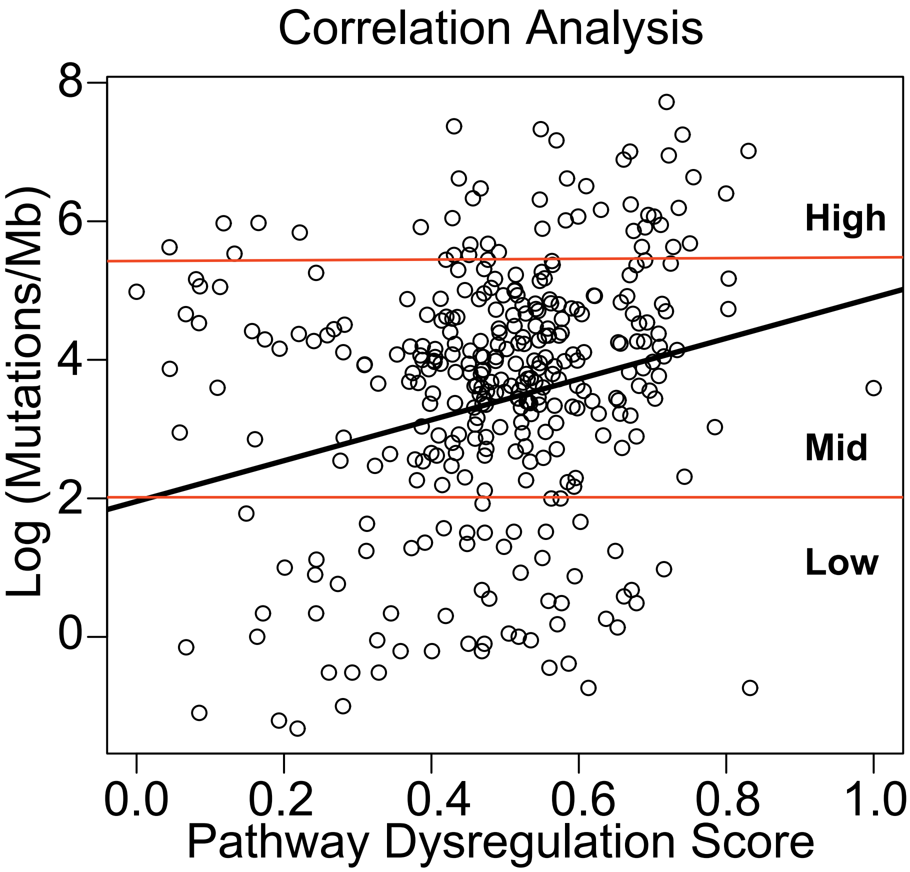

Supplement: Supplementary file 1 — Fig. S1. FACS of DNA content of synchronized populations. Fig. S2. Immunoblots of synchronized populations. Fig. S3. Cell counts from siRNA controls. Fig. S4. Immunofluorescence staining for RPA foci. Fig. S5. Scheme for siRNA transfection and lentivirus transduction gene overexpression functional experiments. Fig. S6. Cell counts from siRNA screen. Fig. S7. DNA content from siRNA screen. Fig. S8. Plots of fold change of RPA foci numbers against p value. Fig. S9. Transduction rate for lentiviral transductions. Fig. S10. DNA content of transduced cells. Fig. S11. V5 tag immunostaining of transduced cells. Fig. S12. Validation of the human ARPP19 antibody. Fig. S13. Quantitative real‐time RT‐PCR of MASTL pathway components. Fig. S14. Deconvolution of ARPP‐19 siRNAs. Fig. S15. Expression level of UV‐G2 checkpoint components from TCGA melanoma data. Fig. S16. Scatter plot showing the correlation between the samples pathway dysregulation score (PDS) and the number of UV signature mutations (Signature 7). [file MOL2-14-22-s001.pdf]
