# Supplementary material for: Multiple interaction nodes define the postreplication repair response to UV‐induced DNA damage that is defective in melanomas and correlated with UV signature mutation load
Source: Mol Oncol. 2019 Dec 19;14(1):22–41. doi: 10.1002/1878-0261.12601 (PMC6944116; doi:10.1002/1878-0261.12601)
Supplement: Supplementary file 2 — Table S1. Total RNA asynchronous vs UV. Table S2. Total RNA asynchronous vs G2. Table S3. Polysome RNA asynchornus vs UV. Table S4. Polysome RNA asynchornus vs G2. Table S5. RNA‐seq of polysome RNA. Table S6. Final gene list of UV‐G2 regulated polysome transcripts. Table S7. Final gene list of UV‐G2 regulated polysome transcripts, siRNA and overexpression list. Table S8. SiRNA and overexpression scoring scheme. Table S9. Results of overexpression screen. Table S10. Functional gene interactions. Table S11. Validated damage repair and checkpoint gene list. Table S12. Pathway dysregulation score for all genes vs mutational load. Table S13. Pathway dysregulation score for pathway subsets vs mutational load. Table S14. Individual gene dysregulation score vs mutational load. [file MOL2-14-22-s002.pdf]

Supplementary Table S1

Total RNA - Asynch vs UV   B value >0 & Fold Change >2

ID	geneSymbol	logFC	AveExpr	t	P.Value	B
1580504	AACS	0.1960	7.1844	1.5769	0.1466	-2.5819
6480059	ACTA2	-2.2557	10.0889	-17.5026	0.0000	10.1858
1030241	ADK	1.0088	7.9825	8.5027	0.0000	6.1462
6520215	ANXA1	-1.3643	9.5855	-10.4024	0.0000	7.4630
2230156	C15ORF52	-1.1203	8.8224	-9.0185	0.0000	6.5410
1450682	C18ORF56	-1.1196	9.1665	-8.8517	0.0000	6.4166
3610372	C3ORF26	1.1393	8.7845	9.3193	0.0000	6.7576
1230047	CBS	1.0551	8.7694	8.9056	0.0000	6.4572
3170110	CCNG1	-1.0138	9.1664	-8.6489	0.0000	6.2612
4230201	CDKN1A	-2.5985	12.1894	-22.4458	0.0000	11.0654
5700753	CEACAM1	-1.0018	9.9264	-8.5400	0.0000	6.1758
450072	CHCHD6	1.1112	9.6034	8.7129	0.0000	6.3108
6510400	CPN1	1.2889	8.8404	10.1357	0.0000	7.2995
2640292	CTGF	-1.3213	8.5900	-11.1190	0.0000	7.8723
3930605	CYR61	-1.0303	8.1170	-8.6395	0.0000	6.2539
3290037	DNAJC7	-1.0193	8.0013	-7.8230	0.0000	5.5774
4280482	DRAM	-1.2990	8.2767	-11.1479	0.0000	7.8879
2640500	DTD1	1.0374	8.0602	8.7338	0.0000	6.3268
5340373	DYM	1.0193	9.0993	8.4054	0.0000	6.0683
3450600	DYNC1H1	-1.1963	7.8856	-6.6826	0.0001	4.4833
6980279	ERVK6	-1.2260	8.5027	-8.9534	0.0000	6.4928
7040678	FABP7	1.7240	8.9723	10.7535	0.0000	7.6688
6110025	FHL2	-1.1428	10.5053	-9.2724	0.0000	6.7245
4860196	FLJ44124	-1.0368	8.1549	-4.8748	0.0007	2.3308
5220767	FLNB	-1.0385	8.4743	-8.4098	0.0000	6.0718
2060121	FUCA1	-1.0547	8.5855	-8.0436	0.0000	5.7684
5090671	GDF15	-1.5629	13.2870	-12.8351	0.0000	8.6990
6370270	GJC1	-1.0795	11.2439	-8.4903	0.0000	6.1364
6840156	GMDS	2.2093	9.2357	15.4487	0.0000	9.6395
5490768	GPR56	1.2737	10.6207	10.7337	0.0000	7.6575
4070241	GYG2	1.0277	9.9131	7.4166	0.0000	5.2086
6220201	HEY1	1.1361	8.2463	8.6820	0.0000	6.2869
1820592	HIST2H2AA3	-1.0959	8.7276	-9.0309	0.0000	6.5501
2350066	HLA-A	-1.0162	8.8084	-6.1400	0.0001	3.8942
2340731	IARS2	-1.1160	10.5157	-5.7603	0.0002	3.4535
4480288	ISG20L1	-1.1693	9.1544	-9.6386	0.0000	6.9773
6020482	KHDRBS3	-1.0221	7.7697	-7.5614	0.0000	5.3426
6480142	LAMP2	-1.0108	8.7033	-7.6003	0.0000	5.3781
10768	LARGE	1.2851	8.6566	10.9900	0.0000	7.8017
4880537	LIMA1	-1.0092	8.9552	-7.3572	0.0000	5.1528
1770754	LIMCH1	-2.0084	8.5551	-14.9789	0.0000	9.4934
4880477	LOC643031	-2.2973	10.1574	-19.0537	0.0000	10.5167
780435	LSAMP	1.4641	8.2750	10.8754	0.0000	7.7379
940735	MGP	1.5647	8.9881	13.2686	0.0000	8.8786
6580131	MRPL22	-1.2397	11.0411	-8.5540	0.0000	6.1869
4290037	NOV	-1.0972	8.1641	-9.2886	0.0000	6.7360

1780411	PEMT	1.1706	9.6784	8.6963	0.0000	6.2979
1170619	PFDN5	-1.1568	10.5308	-9.7396	0.0000	7.0447
2470678	PI15	-1.4849	10.5718	-8.6210	0.0000	6.2394
10673	PLXNB2	-1.1709	8.7980	-9.5087	0.0000	6.8892
1030026	PRR6	1.1077	10.6044	9.3770	0.0000	6.7981
6580646	PSMC5	-1.5147	8.9710	-12.9986	0.0000	8.7680
5570678	RPS27L	-1.3485	12.7068	-10.1865	0.0000	7.3312
4810615	SLC25A44	-1.1305	9.0554	-9.7765	0.0000	7.0691
3370092	STK39	1.1778	8.3996	9.2653	0.0000	6.7195
5810685	THBS1	-1.0536	7.9414	-7.3479	0.0000	5.1441
3360112	TMEM2	-1.2329	9.3727	-9.9297	0.0000	7.1689
2600463	TNFRSF10B	-1.3051	9.4308	-9.5992	0.0000	6.9508
1260020	TP53I3	-1.1876	8.0339	-6.2501	0.0001	4.0174
610519	TPM1	-1.2389	9.1419	-9.8191	0.0000	7.0970
3390128	TPM2	-1.1753	10.2302	-5.4409	0.0003	3.0640

Supplementary Table S2

Total RNA - Asynch vs G2 **B value >0 & Fold Change >2**

ID	geneSymbol	logFC	AveExpr	t	P.Value	B
1580504	AACS	-0.090	7.327	-0.667	0.516	-3.489
6900241	ABCB9	-1.490	9.114	-10.596	0.000	9.726
6480059	ACTA2	-2.309	10.116	-17.794	0.000	13.506
580561	ADCY1	1.154	8.596	9.173	0.000	8.478
1400446	ADFP	-1.337	9.592	-8.096	0.000	7.366
3170068	APOBEC3B	-1.079	8.141	-7.714	0.000	6.933
4230451	ARMCX1	-1.185	8.775	-8.956	0.000	8.266
1450682	C18ORF56	-1.722	9.468	-13.820	0.000	11.831
3610035	CD58	-1.065	9.235	-7.424	0.000	6.589
2070520	CDCA7	1.629	9.806	12.955	0.000	11.347
4230201	CDKN1A	-2.594	12.187	-20.327	0.000	14.240
2640292	CTGF	-1.663	8.761	-13.117	0.000	11.442
3930605	CYR61	-1.156	8.180	-9.237	0.000	8.539
6370356	EEF1A2	-1.191	8.523	-9.129	0.000	8.435
870338	EGR1	-1.654	7.832	-11.280	0.000	10.247
7610131	EPAS1	-1.211	8.793	-9.438	0.000	8.728
7040678	FABP7	1.002	9.333	7.778	0.000	7.007
4880673	GADD45A	-1.489	8.911	-9.319	0.000	8.617
360047	GAS1	1.028	9.089	7.481	0.000	6.657
5090671	GDF15	-1.328	13.169	-10.341	0.000	9.518
290730	HIST1H2BD	-1.068	8.926	-8.003	0.000	7.262
3800347	HIST1H2BJ	-1.101	8.684	-8.370	0.000	7.664
610451	HIST2H2AA3	-1.045	9.129	-7.909	0.000	7.156
2190674	IGFBP5	1.071	9.453	8.580	0.000	7.886
1340743	IL8	-1.829	11.262	-13.695	0.000	11.764
4480288	ISG20L1	-1.011	9.075	-7.967	0.000	7.223
7560632	ITK	-1.088	8.155	-8.727	0.000	8.037
6480142	LAMP2	-1.079	8.737	-7.735	0.000	6.957
6350161	LCP1	-1.109	8.241	-8.474	0.000	7.774
2810601	LEF1	1.131	9.340	8.037	0.000	7.301
2600136	LOC143666	-1.053	7.837	-7.955	0.000	7.209
4180563	LOC613037	1.152	9.769	7.104	0.000	6.194
4880477	LOC643031	-2.411	10.214	-16.082	0.000	12.879
1690465	LOC650369	1.112	10.332	7.303	0.000	6.441
2600291	MTAP	1.033	9.325	8.042	0.000	7.306
5420095	MYC	1.757	8.733	11.614	0.000	10.486
3120458	NCL	1.029	7.983	5.613	0.000	4.134
4290037	NOV	-1.742	8.486	-13.625	0.000	11.727
2630044	NRIP3	-1.108	8.227	-8.517	0.000	7.820
5860187	PHYH	-1.020	8.102	-7.911	0.000	7.159
3850064	PLCXD1	1.111	8.432	6.866	0.000	5.890
1030026	PRR6	1.062	10.627	8.308	0.000	7.597
520561	RAI1	1.011	8.471	8.040	0.000	7.304
4070215	RGS12	-1.044	10.248	-8.218	0.000	7.500
3400019	RGS2	-1.067	8.552	-7.569	0.000	6.762
2640719	RN7SK	-1.567	8.289	-12.583	0.000	11.123

5570678	RPS27L	-1.094	12.579	-7.972	0.000	7.228
4290072	SERTAD1	-2.245	9.405	-15.875	0.000	12.794
6330504	SERTAD4	-1.119	7.676	-7.649	0.000	6.857
5490546	SLC30A1	-1.188	8.502	-9.063	0.000	8.372
990468	SPATS1	-1.573	7.975	-11.091	0.000	10.107
130519	STAT2	-1.085	9.276	-8.498	0.000	7.799
5810685	THBS1	-1.124	7.977	-8.616	0.000	7.923
2680110	TM4SF1	-1.030	11.775	-6.802	0.000	5.807
3360112	TMEM2	-1.653	9.583	-12.163	0.000	10.856
3360681	TNFAIP3	-1.068	7.847	-7.697	0.000	6.913
3780092	TNFRSF21	1.547	9.733	12.072	0.000	10.796
6660630	TP53INP1	-1.413	7.957	-10.748	0.000	9.845
4760747	TPST1	-1.031	9.387	-7.946	0.000	7.199
2570328	TRIB2	1.311	9.471	10.060	0.000	9.282
2230288	TST	-1.130	9.546	-8.420	0.000	7.717
1230544	UBL3	1.205	10.480	9.173	0.000	8.478
4220437	UCN2	-1.152	8.180	-8.405	0.000	7.702

Supplementary Table S3

Polysome RNA - Asynch vs B value >0 & Fold Change >2

ID	geneSymbol	logFC	AveExpr	t	P.Value	B
290603	AARS	-1.146	10.239	-8.688	0.000	3.308
4560681	AARS2	-1.008	7.622	-6.845	0.001	2.370
6900241	ABCB9	-1.056	9.132	-6.363	0.001	2.068
1170647	ACADVL	-1.143	9.650	-8.962	0.000	3.423
7050082	ACP5	-1.264	8.294	-10.831	0.000	4.078
5220377	ACSS1	-1.015	8.078	-8.175	0.000	3.077
6480059	ACTA2	-2.737	9.739	-14.416	0.000	4.897
5080364	ACTN1	-1.692	10.976	-13.757	0.000	4.778
270437	ACTN4	-1.378	8.727	-7.084	0.001	2.510
2900450	ADAR	-1.238	9.238	-7.209	0.001	2.581
5900685	ADD1	-1.210	10.602	-7.856	0.000	2.923
1030241	ADK	1.237	8.035	7.048	0.001	2.490
1170440	AHCYL1	-1.497	10.627	-6.124	0.002	1.908
1500689	AIFM1	-1.120	9.358	-7.724	0.001	2.856
6590253	ALDOA	-1.029	11.862	-7.187	0.001	2.569
3870619	AMD1	1.437	8.579	7.792	0.001	2.891
730725	KDM1A	-1.230	8.928	-10.152	0.000	3.864
2190241	AP1M1	-1.118	9.011	-5.214	0.003	1.229
940706	AP1S2	1.286	11.747	4.346	0.007	0.463
6100719	AP3D1	-1.101	8.728	-8.281	0.000	3.126
5960709	APITD1	1.468	9.486	7.643	0.001	2.815
730414	APOE	-1.071	11.219	-7.489	0.001	2.734
1660685	ARHGAP15	1.411	8.312	9.923	0.000	3.786
6130725	ARHGEF18	-1.487	9.271	-7.916	0.000	2.952
2630209	ARL5A	1.141	7.641	5.939	0.002	1.779
2140368	ARPP-19	1.537	8.758	4.774	0.005	0.856
6960242	ASNSD1	1.457	9.290	5.591	0.002	1.525
1410348	ATG12	1.377	9.136	7.883	0.000	2.936
4210095	ATP1A1	-1.150	11.701	-8.982	0.000	3.431
6590201	ATP6AP1	-1.199	11.799	-8.563	0.000	3.253
1660215	ATP6V0A1	-1.179	8.723	-8.163	0.000	3.072
6650017	ATP6V1B2	-1.043	11.435	-8.721	0.000	3.322
3390605	AZI1	-1.367	8.704	-7.442	0.001	2.709
2970040	B3GALNT1	1.026	7.630	6.575	0.001	2.204
1340075	BAG3	-1.168	9.867	-7.212	0.001	2.583
2710746	BCAR1	-1.112	7.956	-5.503	0.003	1.458
4830687	BCAS3	1.016	8.658	8.414	0.000	3.187
4220270	BIVM	1.231	8.191	5.214	0.003	1.229
1090292	BMI1	1.152	8.795	4.347	0.007	0.464
7380136	BNIP2	1.023	9.302	4.392	0.007	0.507
1660435	BOP1	-1.306	10.257	-5.437	0.003	1.406
1010487	BTG2	-1.587	8.093	-8.113	0.000	3.048
7400341	C11ORF2	-1.067	9.706	-4.494	0.006	0.602
5700301	GSKIP	1.640	8.814	6.717	0.001	2.292
1410050	C14ORF147	1.226	9.379	4.942	0.004	1.002
3060646	C14ORF173/INI	-1.461	9.222	-6.262	0.001	2.002

360373	C14ORF85	-1.102	8.914	-4.917	0.004	0.981
2230156	CCDC9B	-1.500	8.732	-8.383	0.000	3.173
6520497	C16ORF63	1.029	9.716	4.352	0.007	0.469
3440703	C17ORF58	1.412	10.164	10.681	0.000	4.033
3450300	C18ORF19	1.005	8.530	4.963	0.004	1.020
4220364	C19ORF2	1.142	10.501	4.113	0.009	0.236
3870112	C1ORF19	1.499	9.290	7.035	0.001	2.482
5550204	C20ORF116	-1.121	9.318	-4.180	0.008	0.303
7000403	C20ORF45	1.351	9.563	4.869	0.004	0.939
5810632	RTCB	-1.161	9.202	-9.359	0.000	3.580
2350142	C2ORF32	1.212	9.464	9.123	0.000	3.488
520224	CMSS1	1.591	8.516	7.784	0.001	2.887
2650605	C4ORF18	1.170	9.247	4.352	0.007	0.470
940471	C5ORF13	1.096	9.504	6.583	0.001	2.210
780187	C6ORF130	1.224	9.215	8.447	0.000	3.202
7150017	C6ORF48	1.240	11.996	7.089	0.001	2.513
430309	C6ORF66	1.003	9.939	7.498	0.001	2.739
3140280	C9ORF6	1.207	7.812	7.085	0.001	2.511
1570672	CAPN1	-1.564	8.437	-7.084	0.001	2.510
4180762	CAPN3	1.151	11.674	8.844	0.000	3.374
2600537	CBFB	1.040	8.762	7.305	0.001	2.634
630086	CCDC23	1.066	8.906	7.812	0.001	2.901
5130202	CCDC25	1.104	8.899	6.884	0.001	2.393
1780730	CCDC59	1.088	9.461	4.230	0.008	0.351
6450397	CCNB1IP1	1.182	9.581	6.027	0.002	1.841
2370204	CCNC	1.133	8.885	4.670	0.005	0.763
5820601	CCND1	-1.138	9.317	-6.175	0.002	1.943
4760154	CCNE2	1.023	8.117	4.079	0.009	0.203
5270717	CCT3	-1.109	7.490	-9.355	0.000	3.578
4290358	CCT7	-1.156	11.596	-6.163	0.002	1.935
6960630	CD97	-1.466	8.974	-8.118	0.000	3.050
1500010	CDC20	-1.353	10.847	-7.466	0.001	2.721
4230201	CDKN1A	-1.932	12.765	-14.180	0.000	4.856
5700753	CEACAM1	-1.269	9.034	-8.920	0.000	3.405
4230431	CEBPG	1.236	7.789	5.135	0.003	1.164
6900392	CHCHD5	1.114	9.265	8.913	0.000	3.402
450072	CHCHD6	1.595	9.824	7.444	0.001	2.710
2060088	CLCN7	-1.200	11.814	-6.245	0.001	1.990
4810286	CLDND1	1.060	9.579	5.597	0.002	1.529
3440138	CLK1	1.286	8.143	4.250	0.008	0.371
770168	CLPTM1	-1.123	9.775	-8.470	0.000	3.212
1470184	CLSTN1	-1.407	9.893	-11.544	0.000	4.279
4290403	CMTM7	1.073	8.284	6.489	0.001	2.150
3390017	CNDP2	-1.123	10.752	-6.846	0.001	2.371
5550037	CNN2	-1.364	9.158	-6.173	0.002	1.941
4590241	CNOT7	1.578	8.949	5.788	0.002	1.671
5910431	COL4A1	-1.049	8.841	-5.470	0.003	1.431
3360093	COMMD1	1.075	10.360	8.318	0.000	3.144
6510719	COMMD10	1.297	8.005	10.551	0.000	3.993
1770400	COMMD8	1.257	9.194	4.678	0.005	0.771

4150500	COPA	-1.010	9.303	-7.776	0.001	2.883
6620315	COQ6	-1.030	8.903	-6.980	0.001	2.450
6510400	CPN1	1.451	10.067	10.808	0.000	4.071
3830202	CSAG1	1.034	11.718	5.047	0.004	1.091
4010095	CSAG3A	1.074	8.886	9.245	0.000	3.536
5690687	CTGF	-1.504	8.028	-10.555	0.000	3.994
3890017	CTNNA1	-1.025	9.840	-8.284	0.000	3.128
1110092	CTSD	-1.128	8.575	-6.443	0.001	2.120
6100768	CYFIP2	-1.095	7.553	-7.231	0.001	2.593
3930605	CYR61	-1.383	8.014	-11.985	0.000	4.393
2060091	DAG1	-1.481	9.557	-11.907	0.000	4.374
5290703	DCK	1.015	9.144	4.218	0.008	0.340
2350315	DCTN1	-1.384	8.481	-7.497	0.001	2.738
5820050	DCTN2	-1.138	10.921	-5.218	0.003	1.232
2370100	DDB1	-1.523	10.914	-8.180	0.000	3.079
3710196	DDR1	-1.160	9.715	-4.571	0.006	0.674
3520689	DDX19A	-1.111	8.338	-5.802	0.002	1.681
4390661	DDX51	-1.016	9.135	-5.898	0.002	1.750
60524	DENND1A	1.090	7.953	9.322	0.000	3.566
7330164	DENR	1.086	9.896	5.388	0.003	1.368
1570520	DHX16	-1.424	9.291	-9.644	0.000	3.686
4260142	DHX37	-1.133	8.881	-7.224	0.001	2.589
6290719	DPAGT1	-1.085	9.927	-5.475	0.003	1.435
6510279	DPH3	1.116	9.732	8.677	0.000	3.303
5890300	DPH4	1.131	7.465	6.290	0.001	2.020
2640500	DTD1	1.050	9.062	8.371	0.000	3.168
5340373	DYM	1.064	8.765	7.628	0.001	2.807
7330044	DYNLT3	1.088	8.086	4.734	0.005	0.821
160639	EBAG9	1.330	8.745	5.161	0.003	1.186
5360553	ECH1	-1.032	11.465	-4.726	0.005	0.814
6960037	EDC4	-1.333	8.982	-6.518	0.001	2.168
6370356	EEF1A2	-1.144	7.606	-6.781	0.001	2.331
6980475	EFTUD2	-1.423	9.582	-9.055	0.000	3.460
1780619	EHD1	-1.351	8.785	-5.633	0.002	1.556
5960546	EIF2B4	-1.173	9.552	-6.272	0.001	2.008
6290603	EIF3B	-1.180	11.131	-9.771	0.000	3.732
150577	EIF4G1	-1.299	8.852	-10.342	0.000	3.926
3370291	EWSR1	-1.057	8.988	-7.780	0.001	2.885
7040678	FABP7	2.047	10.105	16.975	0.000	5.267
4230735	FAM129A	-1.032	8.751	-3.919	0.011	0.040
3180053	FAM129B	-1.138	10.578	-6.266	0.001	2.004
60634	FAM18B	1.034	8.077	4.666	0.005	0.760
6550243	FAM62A	-1.541	8.527	-11.371	0.000	4.233
1990079	FBXO32	-1.098	10.460	-6.768	0.001	2.324
1990278	FCGR2A	1.320	8.220	7.144	0.001	2.544
3370703	FEN1	-1.116	11.737	-8.045	0.000	3.015
3310068	FGF12	1.246	8.096	8.766	0.000	3.341
6110025	FHL2	-1.321	10.887	-6.979	0.001	2.449
4880646	FKSG30	-1.768	9.306	-10.288	0.000	3.909
5220767	FLNB	-1.318	9.022	-8.919	0.000	3.405

6860544	FOXK1	-1.002	8.746	-7.038	0.001	2.484
7330026	FRAT2	1.031	9.829	8.996	0.000	3.436
4560328	FSCN1	-1.347	11.548	-7.542	0.001	2.762
2060121	FUCA1	-1.137	8.393	-9.431	0.000	3.607
4880673	GADD45A	-1.025	8.738	-6.049	0.002	1.857
7210767	GAK	-1.363	10.050	-10.112	0.000	3.850
580048	GBA	-1.077	9.400	-5.509	0.003	1.462
5090671	GDF15	-2.405	12.836	-17.391	0.000	5.315
6840156	GMDS	1.276	9.247	7.848	0.000	2.918
5260044	GMFB	1.506	9.877	5.532	0.002	1.480
450348	GNG10	1.172	9.391	7.038	0.001	2.483
5890025	GNG12	1.831	8.805	5.494	0.003	1.450
4050671	GNG4	1.452	8.337	8.088	0.000	3.036
6110392	GNS	-1.082	11.100	-7.901	0.000	2.945
6980048	GOT2	-1.132	10.911	-5.493	0.003	1.450
6840164	GPNMB	-1.070	10.049	-8.240	0.000	3.107
6840184	GRN	-1.481	8.645	-7.111	0.001	2.526
610148	GSDMDC1	-1.031	8.587	-4.461	0.006	0.572
110474	GSS	-1.306	8.597	-7.596	0.001	2.790
4280471	GUK1	1.056	11.169	6.031	0.002	1.844
3940551	HARS	-1.007	9.418	-8.012	0.000	2.999
4850296	HCFC1	-1.178	8.990	-4.782	0.005	0.864
3140056	HDAC1	-1.021	11.039	-6.428	0.001	2.110
5130253	HGS	-1.381	9.965	-7.049	0.001	2.490
5960092	HIATL1	1.151	9.748	8.059	0.000	3.022
7100164	HIGD1A	1.005	10.955	4.839	0.004	0.913
1660196	HK1	-1.092	10.863	-9.048	0.000	3.458
3400438	HLA-A	-1.431	12.036	-6.920	0.001	2.415
5270689	HMGN4	1.030	9.723	5.975	0.002	1.805
6450646	HNRPM	-1.093	10.798	-7.892	0.000	2.940
6420373	HRSP12	1.016	8.810	6.267	0.001	2.005
3130358	HS.25892	1.156	8.805	6.946	0.001	2.430
6960300	HS.279842	1.423	8.293	11.895	0.000	4.370
5550768	HS.294603	1.435	9.564	7.354	0.001	2.661
6860240	HS.546375	1.065	8.252	7.886	0.000	2.938
5560544	HS.551538	-1.280	10.293	-7.418	0.001	2.695
290148	HS.584167	1.015	8.302	5.471	0.003	1.433
6400300	HSCB	1.062	8.810	7.145	0.001	2.545
5220520	HSD11B1L	1.063	8.591	6.059	0.002	1.864
7050332	HSP90AA1	-1.115	10.931	-4.438	0.006	0.551
6380717	HSPA1A	-1.637	8.349	-14.280	0.000	4.873
3850433	HSPA1B	-1.931	9.931	-7.399	0.001	2.685
520189	HYOU1	-1.039	8.615	-7.406	0.001	2.689
650241	IER5	-1.108	8.684	-5.659	0.002	1.575
2810156	IGF2R	-1.150	10.684	-7.880	0.000	2.935
2190674	IGFBP5	-1.214	9.855	-5.090	0.004	1.127
4230132	ILK	-1.044	10.760	-5.323	0.003	1.316
1110538	INCENP	-1.059	7.969	-7.204	0.001	2.578
6100040	INTS1	-1.071	8.294	-5.639	0.002	1.561
6110736	IRS2	-1.176	8.946	-6.681	0.001	2.270

4480288	ISG20L1	-1.254	9.847	-8.522	0.000	3.235
4830239	ITPKB	-1.378	10.692	-7.893	0.000	2.941
1010376	JAG1	-1.048	8.396	-6.942	0.001	2.427
7000064	KCNH6	-1.046	10.349	-4.915	0.004	0.979
4590554	KCTD3	1.164	8.575	5.064	0.004	1.106
7550470	PCLAF	-1.083	12.186	-6.190	0.001	1.953
5890136	ZSWIM8	-1.015	8.277	-5.492	0.003	1.449
5560682	KRT10	1.337	10.736	4.778	0.005	0.860
4120086	LAMC1	-1.169	10.107	-6.999	0.001	2.461
6270100	LAMP1	-1.451	11.999	-10.930	0.000	4.108
3060292	LAMP2	-1.197	10.264	-8.929	0.000	3.409
10768	LARGE	1.073	9.087	8.742	0.000	3.331
4560129	LGMN	-1.191	8.315	-9.739	0.000	3.721
5050113	LIG1	-1.015	8.411	-6.367	0.001	2.071
1740576	LMF2	-1.211	8.836	-7.036	0.001	2.483
2630768	LMNA	-2.229	9.769	-12.095	0.000	4.420
7570148	LMNB2	-1.011	9.699	-6.544	0.001	2.185
7550743	LOC203547	1.095	10.250	4.241	0.008	0.363
1010575	LOC388272	1.061	8.337	5.337	0.003	1.328
5570519	LOC401397	1.332	11.178	4.242	0.008	0.363
670066	LOC641738	1.389	9.686	9.424	0.000	3.604
4880477	LOC643031	-1.898	8.135	-7.227	0.001	2.591
6580544	LOC644774	-1.388	9.203	-4.959	0.004	1.017
3310288	LOC647000	-1.109	10.889	-7.435	0.001	2.705
3060400	LOC649037	1.025	9.162	4.946	0.004	1.005
6250114	LOC650737	1.156	9.729	6.139	0.002	1.918
5390037	LOC653226	1.141	12.105	5.971	0.002	1.802
3930326	LOC728014	-1.075	8.027	-6.075	0.002	1.874
6770746	LOC728715	1.460	11.335	9.110	0.000	3.482
5670397	LOC729148	1.197	9.827	6.982	0.001	2.451
610068	LOC730358	1.075	7.638	4.585	0.006	0.687
70300	LYPLA1	1.299	9.196	8.539	0.000	3.243
6550315	LYRM1	1.044	9.054	5.902	0.002	1.753
3520735	LZTS1	-1.282	9.464	-7.291	0.001	2.626
3800600	MAGED1	-1.258	9.372	-7.873	0.000	2.931
1820360	MAPK9	1.203	8.702	5.567	0.002	1.506
6060484	MARCKS	-1.074	10.162	-6.875	0.001	2.388
1470195	MCM7	-1.227	9.959	-7.122	0.001	2.531
3180446	MED16	-1.245	8.053	-6.976	0.001	2.447
5560075	MFGE8	-1.460	10.115	-8.144	0.000	3.063
940735	MGP	1.438	9.919	8.564	0.000	3.254
3830519	MKKS	1.105	9.267	4.761	0.005	0.845
7330367	MLANA	1.108	12.294	5.729	0.002	1.627
2060082	MRPS10	1.001	9.998	4.553	0.006	0.658
3890500	MRPS21	1.021	10.464	7.175	0.001	2.562
730332	MSN	-1.005	9.932	-7.482	0.001	2.730
990615	MTCP1	1.016	8.501	5.968	0.002	1.800
3190164	MUTED	1.139	7.580	4.790	0.005	0.870
3360646	MVP	-1.097	7.695	-6.150	0.002	1.926
2970730	MYADM	-1.434	9.602	-11.531	0.000	4.276

6620136	MYH9	-1.572	10.176	-12.089	0.000	4.419
3710605	MYO1C	-1.386	9.063	-10.775	0.000	4.062
3360131	NAPRT1	-1.341	9.447	-8.421	0.000	3.190
4180204	NCOR2	-1.401	9.376	-9.080	0.000	3.471
7210719	NCSTN	-1.218	11.383	-7.421	0.001	2.697
540221	NOL1	-1.078	10.052	-5.663	0.002	1.578
7210017	NOMO2	-1.195	9.031	-7.023	0.001	2.475
4560056	NONO	-1.104	10.133	-8.778	0.000	3.346
5080167	NOTCH1	-1.199	8.187	-7.791	0.001	2.890
6520026	NUCB1	-1.339	11.742	-9.312	0.000	3.562
4120750	OBFC1	1.239	8.680	8.644	0.000	3.289
7380470	OSBPL9	1.324	9.327	4.216	0.008	0.338
270524	P15RS	1.043	9.823	3.956	0.010	0.078
4010064	P4HA2	-1.719	9.440	-11.791	0.000	4.344
2850333	P4HB	-1.201	11.830	-10.450	0.000	3.961
5310768	PAF1	-1.010	7.666	-8.638	0.000	3.286
4280692	PARP1	-1.114	9.916	-8.604	0.000	3.272
630364	PCGF6	1.059	8.347	5.538	0.002	1.484
6510546	PCNP	1.551	10.608	5.328	0.003	1.321
5670400	PEPD	-1.023	10.733	-5.248	0.003	1.257
5570242	PFKL	-1.001	7.691	-4.272	0.008	0.393
2360452	PFKP	-1.028	10.616	-8.627	0.000	3.281
3520743	PHLDA3	-1.004	9.396	-7.528	0.001	2.754
1470315	PIAS4	-1.338	9.251	-8.173	0.000	3.076
160170	PKM2	-1.152	9.723	-8.772	0.000	3.343
2510678	PKMYT1	-1.023	8.196	-4.219	0.008	0.341
3710044	PLAT	-1.150	9.174	-9.065	0.000	3.465
4010397	PLEKHG3	-1.262	8.807	-10.067	0.000	3.835
60437	PLOD1	-1.767	11.777	-9.213	0.000	3.523
160630	PLOD3	-1.194	11.266	-7.435	0.001	2.705
10673	PLXNB2	-2.198	9.022	-11.154	0.000	4.172
360280	PNRC2	1.024	8.793	6.656	0.001	2.255
6380338	POLB	1.038	9.288	3.883	0.011	0.003
3870220	POLG	-1.081	7.826	-7.010	0.001	2.467
1090687	POLR1D	1.465	9.089	5.660	0.002	1.576
1990053	POLRMT	-1.302	8.242	-5.976	0.002	1.805
1230754	POR	-1.057	8.157	-6.181	0.002	1.947
830411	PPME1	-1.206	8.509	-8.901	0.000	3.398
5570767	PPP2R1A	-1.070	9.874	-8.339	0.000	3.153
5960343	PRIC285	-1.146	7.910	-7.402	0.001	2.687
630280	PRKCSH	-1.125	8.196	-8.629	0.000	3.282
6060349	PRPF8	-1.145	8.941	-5.874	0.002	1.733
1030026	PRR6	1.115	10.651	8.319	0.000	3.144
6200086	PSAP	-1.341	11.135	-9.579	0.000	3.663
6960315	PSMC3	-1.006	9.654	-4.977	0.004	1.032
450403	PSMD2	-1.543	9.668	-10.454	0.000	3.962
5960739	PSMD4	-1.218	11.621	-8.960	0.000	3.422
1400025	PTOV1	-1.189	10.552	-6.431	0.001	2.113
6180414	PTP4A1	1.662	9.026	6.164	0.002	1.936
2320189	PTP4A2	1.050	11.942	4.294	0.007	0.413

4890707	PVRL2	-1.069	8.788	-8.688	0.000	3.308
1090288	PXMP3	1.262	9.631	8.908	0.000	3.400
4540326	PYGB	-1.551	8.685	-6.742	0.001	2.308
6020719	RAB23	1.191	8.980	5.209	0.003	1.225
2900292	RANGAP1	-1.083	9.814	-6.210	0.001	1.966
780050	RAP2A	1.522	9.341	5.773	0.002	1.660
4880551	RBM42	-1.009	8.979	-8.406	0.000	3.183
1710189	RHBDF1	-1.265	8.888	-5.983	0.002	1.811
380050	RHBDF2	-1.233	8.468	-7.375	0.001	2.672
5050519	RHOQ	1.520	10.719	5.649	0.002	1.568
5560112	RNMTL1	-1.119	8.993	-5.930	0.002	1.773
3290446	RPL36	1.029	8.259	8.909	0.000	3.401
380070	RPS23	1.350	8.944	5.861	0.002	1.723
6020564	RRAGA	-1.162	10.811	-4.849	0.004	0.922
3890408	RRBP1	-1.412	9.210	-11.273	0.000	4.206
1090523	RYK	1.240	9.698	7.730	0.001	2.859
5490431	SAT1	1.140	10.669	6.173	0.002	1.942
4290196	SCRIB	-1.803	8.822	-15.781	0.000	5.110
3190255	SDF4	-1.400	8.175	-4.164	0.008	0.286
3180470	SDHA	-1.844	9.993	-15.590	0.000	5.083
4280722	SEC24C	-1.118	9.465	-6.384	0.001	2.082
1030471	SEC61A1	-1.141	11.680	-8.892	0.000	3.394
6380296	SELT	1.576	8.569	5.865	0.002	1.726
7650017	SERPINH1	-1.153	8.974	-5.178	0.003	1.200
3930070	SF3B2	-1.022	9.647	-5.954	0.002	1.790
1340538	SGSH	-1.072	9.997	-6.417	0.001	2.103
6560301	SH2B3	1.158	10.266	9.679	0.000	3.699
5890242	SH3BGRL	1.110	8.131	6.146	0.002	1.923
1190437	SH3PXD2B	-1.057	8.462	-8.089	0.000	3.036
5560541	SKIV2L	-1.129	8.822	-7.238	0.001	2.597
4730674	SKP2	1.172	8.565	8.262	0.000	3.118
110450	SLC12A9	-1.583	8.891	-8.996	0.000	3.436
6350392	SLC1A4	-1.066	8.243	-8.740	0.000	3.330
4590370	SLC2A1	-1.071	8.503	-6.832	0.001	2.362
6220386	SLC38A10	-1.042	7.652	-6.507	0.001	2.161
5420575	SLC3A2	-1.365	10.200	-8.501	0.000	3.226
4730541	SLC44A1	1.041	10.029	6.381	0.001	2.080
1740692	SLC45A2	-1.028	9.795	-7.159	0.001	2.553
10133	SLC9A1	-1.488	9.605	-9.378	0.000	3.587
6860300	SMARCA4	-1.081	8.661	-7.846	0.000	2.918
1050475	SNHG5	1.224	10.341	7.062	0.001	2.498
3870010	SNRP70	-1.393	10.467	-8.804	0.000	3.357
6770673	SOCS2	1.419	8.852	8.493	0.000	3.223
5690246	SPIRE1	1.124	8.812	6.854	0.001	2.375
2900626	SRI	1.063	8.125	5.592	0.002	1.525
3930358	SRP9	1.256	10.058	7.467	0.001	2.722
4760564	SS18L2	1.012	12.055	8.694	0.000	3.310
5260440	ST3GAL6	1.013	9.373	4.615	0.005	0.714
2070100	ST6GALNAC3	1.390	7.811	11.405	0.000	4.242
5090619	STAT3	-1.061	7.791	-6.383	0.001	2.081

5910619	STX2	1.094	9.163	5.066	0.004	1.107
5900682	SUMO3	1.010	12.194	8.223	0.000	3.100
2680064	SYVN1	-1.119	7.780	-5.148	0.003	1.175
7330392	TAP1	-1.303	10.552	-6.453	0.001	2.127
7650358	TGFBI	-1.028	9.570	-8.120	0.000	3.051
770672	THOP1	-1.253	8.882	-4.268	0.008	0.389
150441	TIGA1	1.515	9.421	7.046	0.001	2.488
4180050	TIMELESS	-1.460	9.370	-8.980	0.000	3.430
70674	TM9SF4	-1.035	7.811	-5.731	0.002	1.629
3360112	TMEM2	-1.023	8.792	-6.127	0.002	1.910
5690358	TMEM5	1.161	11.457	5.397	0.003	1.375
2600463	TNFRSF10B	-1.233	9.592	-7.966	0.000	2.977
4900435	TNFSF13B	1.357	8.578	8.847	0.000	3.375
2360706	TOM1	-1.000	7.885	-4.614	0.005	0.713
3460121	TOMM20	1.124	11.303	6.929	0.001	2.420
1260020	TP53I3	-1.348	8.596	-9.098	0.000	3.478
3390128	TPM2	-1.345	9.910	-8.083	0.000	3.033
670113	TPP1	-1.013	8.526	-8.608	0.000	3.273
3130612	TRIP6	-1.415	10.951	-6.837	0.001	2.365
4390301	TRPV2	-1.284	9.803	-7.874	0.000	2.932
4490577	TUBA1A	-1.324	9.536	-5.668	0.002	1.582
6580474	TUBB	-1.099	11.852	-7.254	0.001	2.606
2630356	TUBGCP2	-1.037	8.680	-4.683	0.005	0.776
6370097	TUFM	-1.066	10.368	-4.946	0.004	1.006
940041	TWSG1	1.112	7.920	6.758	0.001	2.317
1430239	UBC	-1.006	12.770	-6.789	0.001	2.336
5270021	UBE2E2	1.044	8.677	6.083	0.002	1.880
5340050	UBE2V2	1.234	9.199	4.030	0.010	0.154
1230544	UBL3	1.528	10.032	5.416	0.003	1.390
5870600	UBP1	1.112	9.476	5.848	0.002	1.714
4220437	UCN2	1.462	8.093	8.402	0.000	3.182
4280133	UFM1	1.398	7.959	4.186	0.008	0.309
5420450	UGT2B7	1.051	9.794	4.134	0.009	0.257
770440	ULK1	-1.047	9.177	-6.021	0.002	1.837
6380241	UNC45A	-1.122	8.708	-9.150	0.000	3.499
2640411	UNC84B	-1.369	8.833	-8.448	0.000	3.203
2320253	USMG5	1.342	9.061	11.127	0.000	4.165
6560747	USP5	-1.474	9.513	-11.709	0.000	4.323
6980100	VARS	-1.698	8.363	-6.170	0.002	1.939
1500753	VARS2	-1.321	9.152	-7.180	0.001	2.564
4860358	VBP1	1.016	9.434	4.387	0.007	0.502
70592	VCL	-1.378	10.081	-6.876	0.001	2.389
2450093	VCP	-1.173	9.034	-9.518	0.000	3.640
2640369	VIL2	-1.186	8.995	-7.838	0.000	2.914
3890398	WBP2	-1.276	11.642	-4.298	0.007	0.417
7510731	WBP5	1.326	11.178	5.055	0.004	1.097
7150475	WDR1	-1.292	11.489	-9.288	0.000	3.552
2000577	WDR68	-1.193	9.439	-8.410	0.000	3.186
6560441	XPC	-1.222	8.173	-9.945	0.000	3.793
430100	XRCC6	-1.010	8.224	-6.166	0.002	1.937

5290239	XRCC6BP1	1.017	7.864	6.239	0.001	1.986
7050484	ZYX	-1.077	7.768	-6.196	0.001	1.957

Supplementary Table S4

Polysome RNA - Asynch vs (B value >0 & Fold Change >2

ID	geneSymbol	logFC	AveExpr	t	P.Value	B
1580504	AACS	-0.091	7.146	-0.749	0.488	-3.337
5290598	ABCB9	-1.040	8.760	-7.733	0.001	2.492
6480059	ACTA2	-2.062	9.401	-11.094	0.000	3.468
460204	ADFP	-1.182	11.053	-7.574	0.001	2.427
1340537	ALKBH2	1.005	9.356	9.023	0.000	2.946
2630673	ASF1B	-1.088	8.597	-9.727	0.000	3.148
1010487	BTG2	-1.629	8.114	-8.703	0.000	2.845
1450682	C18ORF56	-1.064	9.648	-8.878	0.000	2.901
940471	C5ORF13	1.098	9.502	6.858	0.001	2.105
6960630	CD97	-1.091	8.786	-6.835	0.001	2.093
2070520	CDCA7	1.315	8.900	12.692	0.000	3.751
4230201	CDKN1A	-1.905	12.752	-12.397	0.000	3.705
5700753	CEACAM1	-1.094	8.946	-8.890	0.000	2.905
5910431	COL4A1	-1.251	8.942	-9.363	0.000	3.047
2640292	CTGF	-1.287	8.239	-6.315	0.001	1.824
1110092	CTSD	-1.044	8.532	-5.990	0.002	1.640
4260386	CTSL1	-1.032	12.283	-6.921	0.001	2.135
3930605	CYR61	-1.270	7.958	-8.111	0.000	2.638
4280482	DRAM	-1.185	8.564	-9.644	0.000	3.126
6650196	E2F5	1.028	8.545	8.541	0.000	2.791
3440386	ECM1	-1.207	8.123	-7.536	0.001	2.411
6370356	EEF1A2	-1.228	7.648	-7.663	0.001	2.464
870338	EGR1	-1.355	7.648	-11.633	0.000	3.573
7610131	EPAS1	-1.005	8.721	-7.500	0.001	2.396
7040678	FABP7	1.287	10.485	9.975	0.000	3.212
3780193	FCRLA	-1.208	8.660	-6.102	0.002	1.705
2060121	FUCA1	-1.280	8.464	-10.149	0.000	3.256
4880673	GADD45A	-1.150	8.951	-5.451	0.003	1.302
4780133	GBA	-1.288	10.190	-9.166	0.000	2.990
5090671	GDF15	-1.741	12.504	-13.781	0.000	3.903
6110392	GNS	-1.325	11.222	-11.992	0.000	3.637
1940021	GRN	-1.481	9.197	-7.618	0.001	2.445
3800139	HEXB	-1.083	11.728	-8.650	0.000	2.827
290730	HIST1H2BD	-1.096	9.962	-9.603	0.000	3.114
3800347	HIST1H2BJ	-1.370	9.553	-8.902	0.000	2.909
2350066	HLA-A	-1.350	9.403	-6.490	0.001	1.919
3130358	HS.25892	1.019	8.874	5.421	0.003	1.283
5220520	HSD11B1L	1.043	8.601	5.909	0.002	1.591
1340743	IL8	-1.669	10.458	-10.398	0.000	3.315
7560632	ITK	-1.010	8.460	-7.660	0.001	2.462
6270100	LAMP1	-1.024	11.786	-8.357	0.000	2.727
4290730	LGALS3BP	-1.048	9.142	-5.947	0.002	1.614
4560129	LGMN	-1.159	8.299	-11.102	0.000	3.469

2600136	LOC143666	-1.054	8.328	-8.666	0.000	2.833
2490328	LOC731486	-1.044	9.386	-8.768	0.000	2.866
5560075	MFGE8	-1.265	10.018	-9.381	0.000	3.052
5420095	MYC	1.437	8.122	13.117	0.000	3.814
7210719	NCSTN	-1.169	11.359	-6.960	0.001	2.154
7570358	NPC1	-1.051	8.776	-6.391	0.001	1.866
7610561	OAT	-1.053	10.319	-6.877	0.001	2.114
10673	PLXNB2	-1.119	8.483	-4.492	0.007	0.593
3290338	POLR2A	-1.051	9.110	-7.922	0.001	2.566
5960343	PRIC285	-1.061	7.867	-6.381	0.001	1.860
6200086	PSAP	-1.153	11.041	-6.305	0.001	1.819
450403	PSMD2	-1.096	9.444	-8.394	0.000	2.740
940477	RETSAT	-1.023	8.278	-6.076	0.002	1.690
4070215	RGS12	-1.011	10.383	-8.142	0.000	2.650
360424	SDHA	-1.228	9.545	-11.803	0.000	3.604
4290072	SERTAD1	-1.914	10.432	-16.459	0.000	4.179
6330504	SERTAD4	-1.116	8.242	-10.626	0.000	3.368
5490692	SEZ6L2	-1.329	8.006	-4.209	0.008	0.353
4730674	SKP2	1.242	8.530	9.253	0.000	3.015
5490546	SLC30A1	-1.091	8.147	-10.224	0.000	3.274
990468	SPATS1	-1.385	8.350	-8.604	0.000	2.812
5910619	STX2	1.075	9.172	5.026	0.004	1.007
70672	TCIRG1	-1.148	8.212	-5.935	0.002	1.607
7150433	TCTEX1D2	1.030	8.640	8.496	0.000	2.775
7650358	TGFBI	-1.015	9.563	-9.497	0.000	3.085
150441	TIGA1	1.102	9.628	5.113	0.004	1.069
3360112	TMEM2	-1.482	9.022	-11.134	0.000	3.476
3780092	TNFRSF21	1.388	10.205	9.116	0.000	2.975
6660630	TP53INP1	-1.775	8.288	-10.216	0.000	3.272
5360392	TP53INP2	-1.289	9.561	-7.959	0.001	2.581
670113	TPP1	-1.067	8.553	-9.510	0.000	3.089
2570328	TRIB2	1.045	9.619	8.536	0.000	2.789
4390301	TRPV2	-1.088	9.705	-4.909	0.004	0.920
5260253	TYR	-1.080	10.008	-8.834	0.000	2.887

Supplementary Table S5

RNA seq data of polysome bound mRNA B>1 FC 2

ID	geneSymbol	ogFCUV/AS	FC UV/AS	AveExpr	t	P.Value	adj.P.Val	B
7040678	FABP7	2.05	4.13	10.10	16.98	0.00	0.03	5.27
5890025	GNG12	1.83	3.56	8.81	5.49	0.00	0.04	1.45
6180414	PTP4A1	1.66	3.16	9.03	6.16	0.00	0.03	1.94
5700301	C14ORF129	1.64	3.12	8.81	6.72	0.00	0.03	2.29
450072	CHCHD6	1.60	3.02	9.82	7.44	0.00	0.03	2.71
520224	C3ORF26	1.59	3.01	8.52	7.78	0.00	0.03	2.89
4590241	CNOT7	1.58	2.99	8.95	5.79	0.00	0.04	1.67
6380296	SELT	1.58	2.98	8.57	5.86	0.00	0.04	1.73
6510546	PCNP	1.55	2.93	10.61	5.33	0.00	0.04	1.32
1230544	UBL3	1.53	2.88	10.03	5.42	0.00	0.04	1.39
780050	RAP2A	1.52	2.87	9.34	5.77	0.00	0.04	1.66
5050519	RHOQ	1.52	2.87	10.72	5.65	0.00	0.04	1.57
150441	TIGA1	1.52	2.86	9.42	7.05	0.00	0.03	2.49
5260044	GMFB	1.51	2.84	9.88	5.53	0.00	0.04	1.48
3870112	C1ORF19	1.50	2.83	9.29	7.03	0.00	0.03	2.48
3450176	C17ORF58	1.48	2.80	10.26	10.42	0.00	0.03	3.95
5960709	APITD1	1.47	2.77	9.49	7.64	0.00	0.03	2.81
1090687	POLR1D	1.47	2.76	9.09	5.66	0.00	0.04	1.58
4220437	UCN2	1.46	2.75	8.09	8.40	0.00	0.03	3.18
6770746	LOC728715	1.46	2.75	11.33	9.11	0.00	0.03	3.48
6960242	ASNSD1	1.46	2.75	9.29	5.59	0.00	0.04	1.52
4050671	GNG4	1.45	2.74	8.34	8.09	0.00	0.03	3.04
6510400	CPN1	1.45	2.73	10.07	10.81	0.00	0.03	4.07
940735	MGP	1.44	2.71	9.92	8.56	0.00	0.03	3.25
3870619	AMD1	1.44	2.71	8.58	7.79	0.00	0.03	2.89
6770673	SOCS2	1.42	2.67	8.85	8.49	0.00	0.03	3.22
1660685	ARHGAP15	1.41	2.66	8.31	9.92	0.00	0.03	3.79
2070100	ST6GALNAC3	1.39	2.62	7.81	11.40	0.00	0.03	4.24
1410348	ATG12	1.38	2.60	9.14	7.88	0.00	0.03	2.94
3370687	CGGBP1	1.37	2.59	9.33	6.29	0.00	0.03	2.02
4900435	TNFSF13B	1.36	2.56	8.58	8.85	0.00	0.03	3.37
380070	RPS23	1.35	2.55	8.94	5.86	0.00	0.04	1.72
2320253	USMG5	1.34	2.53	9.06	11.13	0.00	0.03	4.16
160639	EBAG9	1.33	2.51	8.75	5.16	0.00	0.04	1.19
7510731	WBP5	1.33	2.51	11.18	5.05	0.00	0.04	1.10
1990278	FCGR2A	1.32	2.50	8.22	7.14	0.00	0.03	2.54
70300	LYPLA1	1.30	2.46	9.20	8.54	0.00	0.03	3.24
6510719	COMMD10	1.30	2.46	8.00	10.55	0.00	0.03	3.99
6840156	GMDS	1.28	2.42	9.25	7.85	0.00	0.03	2.92
1090288	PXMP3	1.26	2.40	9.63	8.91	0.00	0.03	3.40
3930358	SRP9	1.26	2.39	10.06	7.47	0.00	0.03	2.72
3310068	FGF12	1.25	2.37	8.10	8.77	0.00	0.03	3.34
1090523	RYK	1.24	2.36	9.70	7.73	0.00	0.03	2.86
7150017	C6ORF48	1.24	2.36	12.00	7.09	0.00	0.03	2.51
4120750	OBFC1	1.24	2.36	8.68	8.64	0.00	0.03	3.29
1030241	ADK	1.24	2.36	8.04	7.05	0.00	0.03	2.49
4230431	CEBPG	1.24	2.35	7.79	5.14	0.00	0.04	1.16

4220270	BIVM	1.23	2.35	8.19	5.21	0.00	0.04	1.23
1410050	C14ORF147	1.23	2.34	9.38	4.94	0.00	0.05	1.00
780187	C6ORF130	1.22	2.34	9.21	8.45	0.00	0.03	3.20
1050475	SNHG5	1.22	2.34	10.34	7.06	0.00	0.03	2.50
2350142	C2ORF32	1.21	2.32	9.46	9.12	0.00	0.03	3.49
3140280	C9ORF6	1.21	2.31	7.81	7.09	0.00	0.03	2.51
510114	CCNB1IP1	1.20	2.30	9.67	5.09	0.00	0.04	1.13
1820360	MAPK9	1.20	2.30	8.70	5.57	0.00	0.04	1.51
6020719	RAB23	1.19	2.28	8.98	5.21	0.00	0.04	1.22
450348	GNG10	1.17	2.25	9.39	7.04	0.00	0.03	2.48
4730674	SKP2	1.17	2.25	8.57	8.26	0.00	0.03	3.12
4590554	KCTD3	1.16	2.24	8.58	5.06	0.00	0.04	1.11
5690358	TMEM5	1.16	2.24	11.46	5.40	0.00	0.04	1.38
6560301	SH2B3	1.16	2.23	10.27	9.68	0.00	0.03	3.70
3940050	FLJ46906	1.15	2.22	10.43	5.94	0.00	0.04	1.78
5960092	HIATL1	1.15	2.22	9.75	8.06	0.00	0.03	3.02
4180762	CAPN3	1.15	2.22	11.67	8.84	0.00	0.03	3.37
2630209	ARL5A	1.14	2.21	7.64	5.94	0.00	0.04	1.78
5490431	SAT1	1.14	2.20	10.67	6.17	0.00	0.03	1.94
5890300	DPH4	1.13	2.19	7.46	6.29	0.00	0.03	2.02
5690246	SPIRE1	1.12	2.18	8.81	6.85	0.00	0.03	2.38
3460121	TOMM20	1.12	2.18	11.30	6.93	0.00	0.03	2.42
6510279	DPH3	1.12	2.17	9.73	8.68	0.00	0.03	3.30
1030026	PRR6	1.11	2.17	10.65	8.32	0.00	0.03	3.14
6900392	CHCHD5	1.11	2.16	9.27	8.91	0.00	0.03	3.40
5870600	UBP1	1.11	2.16	9.48	5.85	0.00	0.04	1.71
940041	TWSG1	1.11	2.16	7.92	6.76	0.00	0.03	2.32
5890242	SH3BGRL	1.11	2.16	8.13	6.15	0.00	0.03	1.92
7330367	MLANA	1.11	2.16	12.29	5.73	0.00	0.04	1.63
5130202	CCDC25	1.10	2.15	8.90	6.88	0.00	0.03	2.39
940471	C5ORF13	1.10	2.14	9.50	6.58	0.00	0.03	2.21
5910619	STX2	1.09	2.13	9.16	5.07	0.00	0.04	1.11
60524	DENND1A	1.09	2.13	7.95	9.32	0.00	0.03	3.57
7330164	DENR	1.09	2.12	9.90	5.39	0.00	0.04	1.37
3360093	COMMD1	1.07	2.11	10.36	8.32	0.00	0.03	3.14
4010095	CSAG3A	1.07	2.11	8.89	9.24	0.00	0.03	3.54
4290403	CMTM7	1.07	2.10	8.28	6.49	0.00	0.03	2.15
10768	LARGE	1.07	2.10	9.09	8.74	0.00	0.03	3.33
630086	CCDC23	1.07	2.09	8.91	7.81	0.00	0.03	2.90
5340373	DYM	1.06	2.09	8.77	7.63	0.00	0.03	2.81
2900626	SRI	1.06	2.09	8.12	5.59	0.00	0.04	1.52
5220520	HSD11B1L	1.06	2.09	8.59	6.06	0.00	0.04	1.86
6400300	HSCB	1.06	2.09	8.81	7.14	0.00	0.03	2.54
4810286	CLDND1	1.06	2.09	9.58	5.60	0.00	0.04	1.53
630364	PCGF6	1.06	2.08	8.35	5.54	0.00	0.04	1.48
4280471	GUK1	1.06	2.08	11.17	6.03	0.00	0.04	1.84
2640500	DTD1	1.05	2.07	9.06	8.37	0.00	0.03	3.17
6550315	LYRM1	1.04	2.06	9.05	5.90	0.00	0.04	1.75
4730541	SLC44A1	1.04	2.06	10.03	6.38	0.00	0.03	2.08
2600537	CBFB	1.04	2.06	8.76	7.30	0.00	0.03	2.63
3830202	CSAG1	1.03	2.05	11.72	5.05	0.00	0.04	1.09

7330026	FRAT2	1.03	2.04	9.83	9.00	0.00	0.03	3.44
5270689	HMGN4	1.03	2.04	9.72	5.98	0.00	0.04	1.80
3290446	RPL36	1.03	2.04	8.26	8.91	0.00	0.03	3.40
2970040	B3GALNT1	1.03	2.04	7.63	6.57	0.00	0.03	2.20
360280	PNRC2	1.02	2.03	8.79	6.66	0.00	0.03	2.25
3890500	MRPS21	1.02	2.03	10.46	7.18	0.00	0.03	2.56
6420373	HRSP12	1.02	2.02	8.81	6.27	0.00	0.03	2.01
990615	MTCP1	1.02	2.02	8.50	5.97	0.00	0.04	1.80
4830687	BCAS3	1.02	2.02	8.66	8.41	0.00	0.03	3.19
4760564	SS18L2	1.01	2.02	12.05	8.69	0.00	0.03	3.31
5900682	SUMO3	1.01	2.01	12.19	8.22	0.00	0.03	3.10
3450300	C18ORF19	1.00	2.01	8.53	4.96	0.00	0.05	1.02
430309	C6ORF66	1.00	2.00	9.94	7.50	0.00	0.03	2.74
3930189	EIF4G1	-1.00	0.50	10.09	-7.55	0.00	0.03	2.77
6860544	FOXK1	-1.00	0.50	8.75	-7.04	0.00	0.03	2.48
3520743	PHLDA3	-1.00	0.50	9.40	-7.53	0.00	0.03	2.75
3120133	SNRP70	-1.01	0.50	7.52	-6.91	0.00	0.03	2.41
730332	MSN	-1.01	0.50	9.93	-7.48	0.00	0.03	2.73
3370164	ATP1A1	-1.01	0.50	12.15	-5.94	0.00	0.04	1.78
6960315	PSMC3	-1.01	0.50	9.65	-4.98	0.00	0.05	1.03
3940551	HARS	-1.01	0.50	9.42	-8.01	0.00	0.03	3.00
4560681	AARS2	-1.01	0.50	7.62	-6.85	0.00	0.03	2.37
4880551	RBM42	-1.01	0.50	8.98	-8.41	0.00	0.03	3.18
4150500	COPA	-1.01	0.50	9.30	-7.78	0.00	0.03	2.88
5310768	PAF1	-1.01	0.50	7.67	-8.64	0.00	0.03	3.29
7570148	LMNB2	-1.01	0.50	9.70	-6.54	0.00	0.03	2.18
670113	TPP1	-1.01	0.50	8.53	-8.61	0.00	0.03	3.27
5890136	KIAA0913	-1.01	0.49	8.28	-5.49	0.00	0.04	1.45
5220377	ACSS1	-1.02	0.49	8.08	-8.18	0.00	0.03	3.08
4390661	DDX51	-1.02	0.49	9.14	-5.90	0.00	0.04	1.75
2030093	PKM2	-1.02	0.49	8.03	-8.04	0.00	0.03	3.01
3890563	LAMP2	-1.02	0.49	8.36	-7.78	0.00	0.03	2.88
3290431	UNC45A	-1.02	0.49	8.61	-5.19	0.00	0.04	1.21
3930070	SF3B2	-1.02	0.49	9.65	-5.95	0.00	0.04	1.79
5670400	PEPD	-1.02	0.49	10.73	-5.25	0.00	0.04	1.26
3360112	TMEM2	-1.02	0.49	8.79	-6.13	0.00	0.03	1.91
3890017	CTNNA1	-1.02	0.49	9.84	-8.28	0.00	0.03	3.13
2360452	PFKP	-1.03	0.49	10.62	-8.63	0.00	0.03	3.28
7650358	TGFBI	-1.03	0.49	9.57	-8.12	0.00	0.03	3.05
1740692	SLC45A2	-1.03	0.49	9.80	-7.16	0.00	0.03	2.55
6590253	ALDOA	-1.03	0.49	11.86	-7.19	0.00	0.03	2.57
6620315	COQ6	-1.03	0.49	8.90	-6.98	0.00	0.03	2.45
2630463	PSMD4	-1.03	0.49	11.04	-7.02	0.00	0.03	2.47
70674	TM9SF4	-1.04	0.49	7.81	-5.73	0.00	0.04	1.63
520189	HYOU1	-1.04	0.49	8.62	-7.41	0.00	0.03	2.69
6220386	SLC38A10	-1.04	0.49	7.65	-6.51	0.00	0.03	2.16
6650017	ATP6V1B2	-1.04	0.49	11.43	-8.72	0.00	0.03	3.32
4230132	ILK	-1.04	0.48	10.76	-5.32	0.00	0.04	1.32
770440	ULK1	-1.05	0.48	9.18	-6.02	0.00	0.04	1.84
1010376	JAG1	-1.05	0.48	8.40	-6.94	0.00	0.03	2.43
5910431	COL4A1	-1.05	0.48	8.84	-5.47	0.00	0.04	1.43

6900241	ABCB9	-1.06	0.48	9.13	-6.36	0.00	0.03	2.07
3370291	EWSR1	-1.06	0.48	8.99	-7.78	0.00	0.03	2.88
1190437	SH3PXD2B	-1.06	0.48	8.46	-8.09	0.00	0.03	3.04
1230754	POR	-1.06	0.48	8.16	-6.18	0.00	0.03	1.95
1110538	INCENP	-1.06	0.48	7.97	-7.20	0.00	0.03	2.58
6350392	SLC1A4	-1.07	0.48	8.24	-8.74	0.00	0.03	3.33
6370097	TUFM	-1.07	0.48	10.37	-4.95	0.00	0.05	1.01
4890707	PVRL2	-1.07	0.48	8.79	-8.69	0.00	0.03	3.31
6840164	GPNMB	-1.07	0.48	10.05	-8.24	0.00	0.03	3.11
5570767	PPP2R1A	-1.07	0.48	9.87	-8.34	0.00	0.03	3.15
730414	APOE	-1.07	0.48	11.22	-7.49	0.00	0.03	2.73
4590370	SLC2A1	-1.07	0.48	8.50	-6.83	0.00	0.03	2.36
6100040	INTS1	-1.07	0.48	8.29	-5.64	0.00	0.04	1.56
1340538	SGSH	-1.07	0.48	10.00	-6.42	0.00	0.03	2.10
4210612	AP3D1	-1.07	0.48	8.64	-8.03	0.00	0.03	3.01
6060484	MARCKS	-1.07	0.48	10.16	-6.88	0.00	0.03	2.39
580048	GBA	-1.08	0.47	9.40	-5.51	0.00	0.04	1.46
540221	NOL1	-1.08	0.47	10.05	-5.66	0.00	0.04	1.58
6860300	SMARCA4	-1.08	0.47	8.66	-7.85	0.00	0.03	2.92
3870220	POLG	-1.08	0.47	7.83	-7.01	0.00	0.03	2.47
6110392	GNS	-1.08	0.47	11.10	-7.90	0.00	0.03	2.95
7650484	CCT3	-1.08	0.47	11.55	-8.34	0.00	0.03	3.16
2900292	RANGAP1	-1.08	0.47	9.81	-6.21	0.00	0.03	1.97
6290719	DPAGT1	-1.09	0.47	9.93	-5.47	0.00	0.04	1.44
610451	HIST2H2AA3	-1.09	0.47	9.93	-5.83	0.00	0.04	1.70
1660196	HK1	-1.09	0.47	10.86	-9.05	0.00	0.03	3.46
6450646	HNRPM	-1.09	0.47	10.80	-7.89	0.00	0.03	2.94
6100768	CYFIP2	-1.10	0.47	7.55	-7.23	0.00	0.03	2.59
3360646	MVP	-1.10	0.47	7.69	-6.15	0.00	0.03	1.93
6580474	TUBB	-1.10	0.47	11.85	-7.25	0.00	0.03	2.61
4560056	NONO	-1.10	0.47	10.13	-8.78	0.00	0.03	3.35
2140368	ARPP-19	-1.11	0.47	12.12	6.32	0.00	0.04	1.65
650241	IER5	-1.11	0.46	8.68	-5.66	0.00	0.04	1.58
3520689	DDX19A	-1.11	0.46	8.34	-5.80	0.00	0.04	1.68
2710746	BCAR1	-1.11	0.46	7.96	-5.50	0.00	0.04	1.46
4280692	PARP1	-1.11	0.46	9.92	-8.60	0.00	0.03	3.27
3370703	FEN1	-1.12	0.46	11.74	-8.04	0.00	0.03	3.02
4280722	SEC24C	-1.12	0.46	9.46	-6.38	0.00	0.03	2.08
2190241	AP1M1	-1.12	0.46	9.01	-5.21	0.00	0.04	1.23
2680064	SYVN1	-1.12	0.46	7.78	-5.15	0.00	0.04	1.17
5560112	RNMTL1	-1.12	0.46	8.99	-5.93	0.00	0.04	1.77
1500689	AIFM1	-1.12	0.46	9.36	-7.72	0.00	0.03	2.86
770168	CLPTM1	-1.12	0.46	9.77	-8.47	0.00	0.03	3.21
3390017	CNDP2	-1.12	0.46	10.75	-6.85	0.00	0.03	2.37
630280	PRKCSH	-1.13	0.46	8.20	-8.63	0.00	0.03	3.28
1110092	CTSD	-1.13	0.46	8.57	-6.44	0.00	0.03	2.12
5560541	SKIV2L	-1.13	0.46	8.82	-7.24	0.00	0.03	2.60
6980048	GOT2	-1.13	0.46	10.91	-5.49	0.00	0.04	1.45
4260142	DHX37	-1.13	0.46	8.88	-7.22	0.00	0.03	2.59
2060121	FUCA1	-1.14	0.45	8.39	-9.43	0.00	0.03	3.61
3180053	FAM129B	-1.14	0.45	10.58	-6.27	0.00	0.03	2.00

5820601	CCND1	-1.14	0.45	9.32	-6.18	0.00	0.03	1.94
5820050	DCTN2	-1.14	0.45	10.92	-5.22	0.00	0.04	1.23
1030471	SEC61A1	-1.14	0.45	11.68	-8.89	0.00	0.03	3.39
1170647	ACADVL	-1.14	0.45	9.65	-8.96	0.00	0.03	3.42
6370356	EEF1A2	-1.14	0.45	7.61	-6.78	0.00	0.03	2.33
6060349	PRPF8	-1.14	0.45	8.94	-5.87	0.00	0.04	1.73
5960343	PRIC285	-1.15	0.45	7.91	-7.40	0.00	0.03	2.69
290603	AARS	-1.15	0.45	10.24	-8.69	0.00	0.03	3.31
2810156	IGF2R	-1.15	0.45	10.68	-7.88	0.00	0.03	2.93
3710044	PLAT	-1.15	0.45	9.17	-9.07	0.00	0.03	3.46
7650017	SERPINH1	-1.15	0.45	8.97	-5.18	0.00	0.04	1.20
4290358	CCT7	-1.16	0.45	11.60	-6.16	0.00	0.03	1.93
5810632	C22ORF28	-1.16	0.45	9.20	-9.36	0.00	0.03	3.58
1340075	BAG3	-1.17	0.45	9.87	-7.21	0.00	0.03	2.58
4120086	LAMC1	-1.17	0.44	10.11	-7.00	0.00	0.03	2.46
2450093	VCP	-1.17	0.44	9.03	-9.52	0.00	0.03	3.64
5960546	EIF2B4	-1.17	0.44	9.55	-6.27	0.00	0.03	2.01
6110736	IRS2	-1.18	0.44	8.95	-6.68	0.00	0.03	2.27
1660215	ATP6V0A1	-1.18	0.44	8.72	-8.16	0.00	0.03	3.07
6290603	EIF3B	-1.18	0.44	11.13	-9.77	0.00	0.03	3.73
2640369	VIL2	-1.19	0.44	8.99	-7.84	0.00	0.03	2.91
1400025	PTOV1	-1.19	0.44	10.55	-6.43	0.00	0.03	2.11
4560129	LGMN	-1.19	0.44	8.31	-9.74	0.00	0.03	3.72
2000577	WDR68	-1.19	0.44	9.44	-8.41	0.00	0.03	3.19
160630	PLOD3	-1.19	0.44	11.27	-7.43	0.00	0.03	2.70
7210017	NOMO2	-1.19	0.44	9.03	-7.02	0.00	0.03	2.48
6590201	ATP6AP1	-1.20	0.44	11.80	-8.56	0.00	0.03	3.25
5080167	NOTCH1	-1.20	0.44	8.19	-7.79	0.00	0.03	2.89
2060088	CLCN7	-1.20	0.44	11.81	-6.24	0.00	0.03	1.99
2850333	P4HB	-1.20	0.44	11.83	-10.45	0.00	0.03	3.96
830411	PPME1	-1.21	0.43	8.51	-8.90	0.00	0.03	3.40
5900685	ADD1	-1.21	0.43	10.60	-7.86	0.00	0.03	2.92
1740576	LMF2	-1.21	0.43	8.84	-7.04	0.00	0.03	2.48
2190674	IGFBP5	-1.21	0.43	9.86	-5.09	0.00	0.04	1.13
7210719	NCSTN	-1.22	0.43	11.38	-7.42	0.00	0.03	2.70
5490376	NCOR2	-1.22	0.43	10.89	-8.28	0.00	0.03	3.13
6560441	XPC	-1.22	0.43	8.17	-9.94	0.00	0.03	3.79
1470195	MCM7	-1.23	0.43	9.96	-7.12	0.00	0.03	2.53
730725	AOF2	-1.23	0.43	8.93	-10.15	0.00	0.03	3.86
380050	RHBDF2	-1.23	0.43	8.47	-7.37	0.00	0.03	2.67
2600463	TNFRSF10B	-1.23	0.43	9.59	-7.97	0.00	0.03	2.98
160370	TPM2	-1.24	0.42	11.07	-7.66	0.00	0.03	2.82
2900450	ADAR	-1.24	0.42	9.24	-7.21	0.00	0.03	2.58
3180446	MED16	-1.24	0.42	8.05	-6.98	0.00	0.03	2.45
4480288	ISG20L1	-1.25	0.42	9.85	-8.52	0.00	0.03	3.24
3800600	MAGED1	-1.26	0.42	9.37	-7.87	0.00	0.03	2.93
4010397	PLEKHG3	-1.26	0.42	8.81	-10.07	0.00	0.03	3.83
7050082	ACP5	-1.26	0.42	8.29	-10.83	0.00	0.03	4.08
1710189	RHBDF1	-1.27	0.42	8.89	-5.98	0.00	0.04	1.81
5700753	CEACAM1	-1.27	0.42	9.03	-8.92	0.00	0.03	3.41
990288	FHL2	-1.28	0.41	10.26	-6.79	0.00	0.03	2.34

3520735	LZTS1	-1.28	0.41	9.46	-7.29	0.00	0.03	2.63
4390301	TRPV2	-1.28	0.41	9.80	-7.87	0.00	0.03	2.93
7150475	WDR1	-1.29	0.41	11.49	-9.29	0.00	0.03	3.55
1990053	POLRMT	-1.30	0.41	8.24	-5.98	0.00	0.04	1.81
7330392	TAP1	-1.30	0.41	10.55	-6.45	0.00	0.03	2.13
1660435	BOP1	-1.31	0.40	10.26	-5.44	0.00	0.04	1.41
110474	GSS	-1.31	0.40	8.60	-7.60	0.00	0.03	2.79
5220767	FLNB	-1.32	0.40	9.02	-8.92	0.00	0.03	3.41
1500753	VARS2	-1.32	0.40	9.15	-7.18	0.00	0.03	2.56
4490577	TUBA1A	-1.32	0.40	9.54	-5.67	0.00	0.04	1.58
6960037	EDC4	-1.33	0.40	8.98	-6.52	0.00	0.03	2.17
1470315	PIAS4	-1.34	0.40	9.25	-8.17	0.00	0.03	3.08
2350066	HLA-A	-1.34	0.40	9.40	-6.39	0.00	0.03	2.09
6520026	NUCB1	-1.34	0.40	11.74	-9.31	0.00	0.03	3.56
3360131	NAPRT1	-1.34	0.39	9.45	-8.42	0.00	0.03	3.19
6200086	PSAP	-1.34	0.39	11.13	-9.58	0.00	0.03	3.66
4560328	FSCN1	-1.35	0.39	11.55	-7.54	0.00	0.03	2.76
1260020	TP53I3	-1.35	0.39	8.60	-9.10	0.00	0.03	3.48
1780619	EHD1	-1.35	0.39	8.78	-5.63	0.00	0.04	1.56
1500010	CDC20	-1.35	0.39	10.85	-7.47	0.00	0.03	2.72
7210767	GAK	-1.36	0.39	10.05	-10.11	0.00	0.03	3.85
5550037	CNN2	-1.36	0.39	9.16	-6.17	0.00	0.03	1.94
5420575	SLC3A2	-1.37	0.39	10.20	-8.50	0.00	0.03	3.23
3390605	AZI1	-1.37	0.39	8.70	-7.44	0.00	0.03	2.71
2640411	UNC84B	-1.37	0.39	8.83	-8.45	0.00	0.03	3.20
4830239	ITPKB	-1.38	0.38	10.69	-7.89	0.00	0.03	2.94
270437	ACTN4	-1.38	0.38	8.73	-7.08	0.00	0.03	2.51
70592	VCL	-1.38	0.38	10.08	-6.88	0.00	0.03	2.39
5130253	HGS	-1.38	0.38	9.97	-7.05	0.00	0.03	2.49
3930605	CYR61	-1.38	0.38	8.01	-11.98	0.00	0.03	4.39
2350315	DCTN1	-1.38	0.38	8.48	-7.50	0.00	0.03	2.74
3710605	MYO1C	-1.39	0.38	9.06	-10.78	0.00	0.03	4.06
6580544	LOC644774	-1.39	0.38	9.20	-4.96	0.00	0.05	1.02
2640292	CTGF	-1.39	0.38	8.29	-10.30	0.00	0.03	3.91
1470184	CLSTN1	-1.41	0.38	9.89	-11.54	0.00	0.03	4.28
3890408	RRBP1	-1.41	0.38	9.21	-11.27	0.00	0.03	4.21
1510458	MYH9	-1.41	0.38	9.64	-6.39	0.00	0.03	2.09
3130612	TRIP6	-1.42	0.37	10.95	-6.84	0.00	0.03	2.37
6980475	EFTUD2	-1.42	0.37	9.58	-9.05	0.00	0.03	3.46
1570520	DHX16	-1.42	0.37	9.29	-9.64	0.00	0.03	3.69
270408	P4HA2	-1.42	0.37	8.87	-7.97	0.00	0.03	2.98
2970730	MYADM	-1.43	0.37	9.60	-11.53	0.00	0.03	4.28
6270100	LAMP1	-1.45	0.37	12.00	-10.93	0.00	0.03	4.11
5560075	MFGE8	-1.46	0.36	10.11	-8.14	0.00	0.03	3.06
4180050	TIMELESS	-1.46	0.36	9.37	-8.98	0.00	0.03	3.43
3060646	C14ORF173	-1.46	0.36	9.22	-6.26	0.00	0.03	2.00
6960630	CD97	-1.47	0.36	8.97	-8.12	0.00	0.03	3.05
6560747	USP5	-1.47	0.36	9.51	-11.71	0.00	0.03	4.32
6840184	GRN	-1.48	0.36	8.64	-7.11	0.00	0.03	2.53
2060091	DAG1	-1.48	0.36	9.56	-11.91	0.00	0.03	4.37
6130725	ARHGEF18	-1.49	0.36	9.27	-7.92	0.00	0.03	2.95

10133	SLC9A1	-1.49	0.36	9.60	-9.38	0.00	0.03	3.59
1170440	AHCYL1	-1.50	0.35	10.63	-6.12	0.00	0.03	1.91
2230156	C15ORF52	-1.50	0.35	8.73	-8.38	0.00	0.03	3.17
2370100	DDB1	-1.52	0.35	10.91	-8.18	0.00	0.03	3.08
6550243	FAM62A	-1.54	0.34	8.53	-11.37	0.00	0.03	4.23
450403	PSMD2	-1.54	0.34	9.67	-10.45	0.00	0.03	3.96
4540326	PYGB	-1.55	0.34	8.68	-6.74	0.00	0.03	2.31
1570672	CAPN1	-1.56	0.34	8.44	-7.08	0.00	0.03	2.51
110450	SLC12A9	-1.58	0.33	8.89	-9.00	0.00	0.03	3.44
1010487	BTG2	-1.59	0.33	8.09	-8.11	0.00	0.03	3.05
7320594	ZYX	-1.62	0.32	9.09	-6.01	0.00	0.04	1.83
6380717	HSPA1A	-1.64	0.32	8.35	-14.28	0.00	0.03	4.87
5080364	ACTN1	-1.69	0.31	10.98	-13.76	0.00	0.03	4.78
6980100	VARS	-1.70	0.31	8.36	-6.17	0.00	0.03	1.94
360424	SDHA	-1.71	0.30	9.79	-14.78	0.00	0.03	4.96
60437	PLOD1	-1.77	0.29	11.78	-9.21	0.00	0.03	3.52
4880646	FKSG30	-1.77	0.29	9.31	-10.29	0.00	0.03	3.91
4290196	SCRIB	-1.80	0.29	8.82	-15.78	0.00	0.03	5.11
3850433	HSPA1B	-1.93	0.26	9.93	-7.40	0.00	0.03	2.69
4230201	CDKN1A	-1.93	0.26	12.77	-14.18	0.00	0.03	4.86
6020424	LMNA	-1.93	0.26	10.56	-7.73	0.00	0.03	2.86
10673	PLXNB2	-2.20	0.22	9.02	-11.15	0.00	0.03	4.17
5090671	GDF15	-2.41	0.19	12.84	-17.39	0.00	0.03	5.32
6480059	ACTA2	-2.74	0.15	9.74	-14.42	0.00	0.03	4.90

Supplementary Table S6

Final Gene List of UV G2 phase regulated transcripts associated with polysomes

Gene Symbol	siRNA	OE	Description
AARS			Alanyl-tRNA synthetase
AARS2			Alanyl-tRNA synthetase 2, mitochondrial (putative)
ABCB9			ATP-binding cassette, sub-family B (MDR/TAP), member 9
ACSS1			Acyl-CoA synthetase short-chain family member 1
ACTA2			Actin, alpha 2, smooth muscle, aorta
ACTN1			Actinin, alpha 1
ACTN4			Actinin, alpha 4
ADAR			Adenosine deaminase, RNA-specific
ADK			Adenosine kinase
AHCYL1			S-adenosylhomocysteine hydrolase-like 1
AIFM1			Apoptosis-inducing factor, mitochondrion-associated, 1
ALDOA			Aldolase A, fructose-bisphosphate
AMD1			Adenosylmethionine decarboxylase 1
AP1M1			Adaptor-related protein complex 1, mu 1 subunit
AP1S2			Adaptor related protein complex 1 subunit sigma 2
AP3D1			Adaptor-related protein complex 3, delta 1 subunit
APITD1			Apoptosis-inducing, TAF9-like domain 1
APOE			Apolipoprotein E
ARHGAP15			Rho GTPase activating protein 15
ARHGEF18			Rho/rac guanine nucleotide exchange factor (GEF) 18
ARL5A			ADP-ribosylation factor-like 5A
ARPP-19			Cyclic AMP phosphoprotein, 19 kD
ASNSD1			Asparagine synthetase domain containing 1
ATG12			ATG12 autophagy related 12 homolog (S. cerevisiae)
ATP1A1			ATPase, Na+/K+ transporting, alpha 1 polypeptide
ATP6AP1			ATPase, H+ transporting, lysosomal accessory protein 1
ATP6V0A1			ATPase, H+ transporting, lysosomal V0 subunit a1
ATP6V1B2			ATPase, H+ transporting, lysosomal 56/58kDa, V1 subunit B2
AZI1			5-azacytidine induced 1
B3GALNT1			Beta-1,3-N-acetylgalactosaminyltransferase 1 (globoside blood group)
BAG3			BCL2-associated athanogene 3
BCAR1			Breast cancer anti-estrogen resistance 1
BCAS3			Breast carcinoma amplified sequence 3
BIVM			Basic, immunoglobulin-like variable motif containing
BMI1			BMI1 polycomb ring finger oncogene
BOP1			Block of proliferation 1
BTG2			BTG family, member 2
C10ORF19			Chromosome 10 ORF19
CAPN1			Calpain 1, (mu/I) large subunit
CAPN3			Calpain 3, (p94)
CBFB			Core-binding factor, beta subunit
CCDC23			Coiled-coil domain containing 23
CCDC25			Coiled-coil domain containing 25

CCDC9B/C15ORF52			coiled-coil domain containing 9B
CCNB1IP1			Cyclin B1 interacting protein 1
CCND1			Cyclin D1
CCT3			Chaperonin containing TCP1, subunit 3 (gamma)
CCT7			Chaperonin containing TCP1, subunit 7 (eta)
CD97			CD97 molecule
CDC20			Cell division cycle 20 homolog (S. cerevisiae)
CDKN1A			Cyclin-dependent kinase inhibitor 1A (p21, Cip1)
CDKN2AIPNL			CDKN2A interacting protein N-terminal like
CEACAM1			Carcinoembryonic antigen-related cell adhesion molecule 1 (biliary glycoprotein)
CEBPG			CCAAT/enhancer binding protein (C/EBP), gamma
CHCHD5			Coiled-coil-helix-coiled-coil-helix domain containing 5
CHCHD6			Coiled-coil-helix-coiled-coil-helix domain containing 6
CLCN7			Chloride channel 7
CLDND1			Claudin domain containing 1
CLPTM1			Cleft lip and palate associated transmembrane protein 1
CLSTN1			Calsyntenin 1
CMSS1			cms1 ribosomal small subunit homolog
CMTM7			CKLF-like MARVEL transmembrane domain containing 7
CNDP2			CNDP dipeptidase 2 (metallopeptidase M20 family)
CNN2			Calponin 2
CNOT7			CCR4-NOT transcription complex, subunit 7
CNRIP1			cannabinoid receptor interacting protein 1
COMMD1			Copper metabolism (Murr1) domain containing 1
COMMD10			COMM domain containing 10
COPA			Coatomer protein complex, subunit alpha
CPN1			Carboxypeptidase N, polypeptide 1
CSAG1			Chondrosarcoma associated gene 1
CTNNA1			Catenin (cadherin-associated protein), alpha 1, 102kDa
CTSD			Cathepsin D
CYFIP2			Cytoplasmic FMR1 interacting protein 2
DAG1			Dystroglycan 1 (dystrophin-associated glycoprotein 1)
DCTN1			Dynactin 1 (p150, glued homolog, Drosophila)
DCTN2			Dynactin 2 (p50)
DDB1			Damage-specific DNA binding protein 1, 127kDa
DDX19A			DEAD (Asp-Glu-Ala-As) box polypeptide 19A
DDX51			DEAD (Asp-Glu-Ala-Asp) box polypeptide 51
DENND1A			DENN/MADD domain containing 1A
DENR			Density-regulated protein
DHX16			DEAH (Asp-Glu-Ala-His) box polypeptide 16
DHX37			DEAH (Asp-Glu-Ala-His) box polypeptide 37
DNAJC24/DPH4			DnaJ heat shock protein family (Hsp40) member C24
DPAGT1			Dolichyl-phosphate (UDP-N-acetylglucosamine) N-acetylglucosaminephosphotransferase 1 (GlcNAc-1-P transferase)
DPH3			DPH3, KTI11 homolog (S. cerevisiae)
DTD1			D-tyrosyl-tRNA deacylase 1 homolog (S. cerevisiae)
DYM			Dymeclin

EBAG9			Estrogen receptor binding site associated, antigen, 9
EDC4			Enhancer of mRNA decapping 4
EEF1A2			Eukaryotic translation elongation factor 1 alpha 2
EFTUD2			Elongation factor Tu GTP binding domain containing 2
EHD1			EH-domain containing 1
EIF2B4			Eukaryotic translation initiation factor 2B, subunit 4 delta, 67kDa
EIF3B			Eukaryotic translation initiation factor 3, subunit B
EIF4G1			Eukaryotic translation initiation factor 4 gamma, 1
EWSR1			Ewing sarcoma breakpoint region 1
FABP7			Fatty acid binding protein 7, brain
FAM129A			Family with sequence similarity 129, member A
FAM129B			Family with sequence similarity 129, member B
FAM62A/ESYT1			Extended synaptotagmin 1
FAM210A			family with sequence similarity 210 member A
FBXO32			F-box protein 32
FCGR2A			Fc fragment of IgG, low affinity IIa, receptor (CD32)
FEN1			Flap structure-specific endonuclease 1
FGF12			Fibroblast growth factor 12
FHL2			Four and a half LIM domains 2
FLNB			Filamin B, beta (actin binding protein 278)
FOXK1			Forkhead box K1
FRAT2			Frequently rearranged in advanced T-cell lymphomas 2
FSCN1			Fascin homolog 1, actin-bundling protein (Strongylocentrotus purpuratus)
FUCA1			Fucosidase, alpha-L- 1, tissue
GADD45A			Growth arrest and DNA-damage-inducible, alpha
GAK			Cyclin G associated kinase
GDF15			Growth differentiation factor 15
GMDS			GDP-mannose 4,6-dehydratase
GMFB			Glia maturation factor, beta
GNG10			Guanine nucleotide binding protein (G protein), gamma 10
GNG12			Guanine nucleotide binding protein (G protein), gamma 12
GNG4			Guanine nucleotide binding protein (G protein), gamma 4
GOT2			Glutamic-oxaloacetic transaminase 2, mitochondrial (aspartate aminotransferase 2)
GPNMB			Glycoprotein (transmembrane) nmb
GSKIP			GSK3B interacting protein
GSS			Glutathione synthetase
GUK1			Guanylate kinase 1
HARS			Histidyl-tRNA synthetase
HDAC1			Histone deacetylase 1
HGS			Hepatocyte growth factor-regulated tyrosine kinase substrate
HIATL1			Hippocampus abundant transcript-like 1
HK1			Hexokinase 1
HMGN4			High mobility group nucleosomal binding domain 4
HNRPM			Heterogeneous nuclear ribonucleoprotein M
HRSP12			Heat-responsive protein 12

HSCB			HscB iron-sulfur cluster co-chaperone homolog (E. coli)
HSPA1A			Heat shock 70kDa protein 1A
HSPA1B			Heat shock 70kDa protein 1B
HYOU1			Hypoxia up-regulated 1
IGF2R			Insulin-like growth factor 2 receptor
IGFBP5			Insulin-like growth factor binding protein 5
ILK			Integrin-linked kinase
INCENP			Inner centromere protein antigens 135/155kDa
INF2			inverted formin, FH2 and WH2 domain containing
INTS1			Integrator complex subunit 1
IRS2			Insulin receptor substrate 2
ISG20L1			Interferon stimulated exonuclease gene 20kDa-like 1
ITPKB			Inositol 1,4,5-trisphosphate 3-kinase B
JAG1			Jagged 1 (Alagille syndrome)
JUN			Jun oncogene
KDM1A			Lysine demethylase 1A
LAMC1			Laminin, gamma 1 (formerly LAMB2)
LAMP1			Lysosomal-associated membrane protein 1
LAMP2			Lysosomal-associated membrane protein 2
LARGE			Like-glycosyltransferase
LIG1			Ligase I, DNA, ATP-dependent
LMF2			Lipase maturation factor 2
LMNA			Lamin A/C
LMNB2			Lamin B2
LYPLA1			Lysophospholipase I
LYRM1			LYR motif containing 1
LZTS1			Leucine zipper, putative tumor suppressor 1
MAGED1			Melanoma antigen family D, 1
MAPK9			Mitogen-activated protein kinase 9
MARCKS			Myristoylated alanine-rich protein kinase C substrate
MCM7			Minichromosome maintenance complex component 7
MED16			Mediator complex subunit 16
MFGE8			Milk fat globule-EGF factor 8 protein
MGP			Matrix Gla protein
MLANA			Melan-A
MSN			Moesin
MTCP1			Mature T-cell proliferation 1
MVP			Major vault protein
MYADM			Myeloid-associated differentiation marker
MYH9			Myosin, heavy chain 9, non-muscle
MYO1C			Myosin IC
NAPRT1			Nicotinate phosphoribosyltransferase domain containing 1
NCOR2			Nuclear receptor co-repressor 2
NDUFAF4			NADH:ubiquinone oxidoreductase complex assembly factor 4
NOL1			Nucleolar protein 1, 120kDa
NONO			Non-POU domain containing, octamer-binding
NOTCH1			Notch homolog 1, translocation-associated (Drosophila)

NUCB1			Nucleobindin 1
OARD1			O-acyl-ADP-ribose deacylase 1
OBFC1			Oligonucleotide/oligosaccharide-binding fold containing 1
P4HA2			Procollagen-proline, 2-oxoglutarate 4-dioxygenase (proline 4-hydroxylase), alpha polypeptide II
P4HB			Procollagen-proline, 2-oxoglutarate 4-dioxygenase (proline 4-hydroxylase), beta polypeptide
PAF1			Paf1, RNA polymerase II associated factor, homolog (S. cerevisiae)
PARP1			Poly (ADP-ribose) polymerase family, member 1
PCGF6			Polycomb group ring finger 6
PCLAF			PCNA clamp associated factor
PCNP			PEST proteolytic signal containing nuclear protein
PEPD			Peptidase D
PFKP			Phosphofructokinase, platelet
PHLDA3			Pleckstrin homology-like domain, family A, member 3
PIAS4			Protein inhibitor of activated STAT, 4
PKM2			Pyruvate kinase, muscle
PLAT			Plasminogen activator, tissue
PLEKHG3			Pleckstrin homology domain containing, family G (with RhoGef domain) member 3
PLOD1			Procollagen-lysine 1, 2-oxoglutarate 5-dioxygenase 1
PLOD3			Procollagen-lysine, 2-oxoglutarate 5-dioxygenase 3
PLXNB2			Plexin B2
PNRC2			Proline-rich nuclear receptor coactivator 2
POLG			Polymerase (DNA directed), gamma
POLR1D			RNA polymerase I and III subunit D
POLRMT			Polymerase (RNA) mitochondrial (DNA directed)
POR			P450 (cytochrome) oxidoreductase
PPME1			Protein phosphatase methylesterase 1
PPP2R1A			Protein phosphatase 2 (formerly 2A), regulatory subunit A , alpha isoform
PRKCSH			Protein kinase C substrate 80K-H
PRPF8			PRP8 pre-mRNA processing factor 8 homolog (S. cerevisiae)
PSAP			Prosaposin (variant Gaucher disease and variant metachromatic leukodystrophy)
PSMC3			Proteasome (prosome, macropain) 26S subunit, ATPase, 3
PSMD2			Proteasome (prosome, macropain) 26S subunit, non-ATPase, 2
PSMD4			Proteasome (prosome, macropain) 26S subunit, non-ATPase, 4
PSMD4			Proteasome (prosome, macropain) 26S subunit, non-ATPase, 4
PTOV1			Prostate tumor overexpressed gene 1
PTP4A1			Protein tyrosine phosphatase type IVA, member 1
PVRL2			Poliovirus receptor-related 2 (herpesvirus entry mediator B)
PEX2			peroxisomal biogenesis factor 2
PYGB			Phosphorylase, glycogen; brain
RAB23			RAB23, member RAS oncogene family

RAC1			Ras-related C3 botulinum toxin substrate 1 (rho family, small GTP binding protein Rac1)
RANGAP1			Ran GTPase activating protein 1
RAP2A			RAP2A, member of RAS oncogene family
RB1			Retinoblastoma protein 1
RBM42			RNA binding motif protein 42
RHBDF1			Rhomboid 5 homolog 1 (Drosophila)
RHOQ			Ras homolog gene family, member Q
RPL36			Ribosomal protein L36
RPS23			Ribosomal protein S23
RRBP1			Ribosome binding protein 1 homolog 180kDa (dog)
RTCB			RNA 2',3'-cyclic phosphate and 5'-OH ligase
RYK			RYK receptor-like tyrosine kinase
SAT1			Spermidine/spermine N1-acetyltransferase 1
SCRIB			Scribbled homolog (Drosophila)
SDHA			Succinate dehydrogenase complex, subunit A, flavoprotein (Fp)
SEC24C			SEC24 related gene family, member C (S. cerevisiae)
SEC61A1			Sec61 alpha 1 subunit (S. cerevisiae)
SERPINH1			Serpin peptidase inhibitor, clade H (heat shock protein 47), member 1, (collagen binding protein 1)
SF3B2			Splicing factor 3b, subunit 2, 145kDa
SGSH			N-sulfoglucosamine sulfohydrolase (sulfamidase)
SH2B3			SH2B adaptor protein 3
SH3BGRL			SH3 domain binding glutamic acid-rich protein like
SH3PXD2B			SH3 and PX domains 2B
SKIV2L			Ski2 like RNA helicase
SKP2			S-phase kinase-associated protein 2 (p45)
SLC12A9			Solute carrier family 12 (potassium/chloride transporters), member 9
SLC1A4			Solute carrier family 1 (glutamate/neutral amino acid transporter), member 4
SLC2A1			Solute carrier family 2 (facilitated glucose transporter), member 1
SLC38A10			Solute carrier family 38 member 10
SLC3A2			Solute carrier family 3 (activators of dibasic and neutral amino acid transport), member 2
SLC44A1			Solute carrier family 44, member 1
SLC45A2			Solute carrier family 45, member 2
SLC9A1			Solute carrier family 9 (sodium/hydrogen exchanger), member 1 (antiporter, Na+/H+, amiloride sensitive)
SMARCA4			SWI/SNF related, matrix associated, actin dependent regulator of chromatin, subfamily a, member 4
SNHG5			Small nucleolar RNA host gene (non-protein coding) 5
SNRP70			Small nuclear ribonucleoprotein U1 subunit 70
SOCS2			Suppressor of cytokine signaling 2
SPIRE1			Spire homolog 1 (Drosophila)
SPTSSA			Serine palmitoyltransferase small subunit A
SRI			Sorcin
SRP9			Signal recognition particle 9kDa

SS18L2			Synovial sarcoma translocation gene on chromosome 18-like 2
ST6GALNAC3			ST6 (alpha-N-acetyl-neuraminyl-2,3-beta-galactosyl-1,3)-N-acetylgalactosaminide alpha-2,6-sialyltransferase 3
STAT3			Signal transducer and activator of transcription 3 (acute-phase response factor)
SUMO3			SMT3 suppressor of mif two 3 homolog 3 (S. cerevisiae)
SYVN1			Synovial apoptosis inhibitor 1, synoviolin
TAP1			Transporter 1, ATP-binding cassette, sub-family B (MDR/TAP)
TIMELESS			Timeless homolog (Drosophila)
TM9SF4			Transmembrane 9 superfamily protein member 4
TMEM2			Transmembrane protein 2
TMEM5			Transmembrane protein 5
TNFRSF10B			Tumor necrosis factor receptor superfamily, member 10b
TNFSF13B			Tumor necrosis factor (ligand) superfamily, member 13b
TOMM20			Translocase of outer mitochondrial membrane 20 homolog (yeast)
TP53I3			Tumor protein p53 inducible protein 3
TPM2			Tropomyosin 2 (beta)
TRIP6			Thyroid hormone receptor interactor 6
TRPV2			Transient receptor potential cation channel, subfamily V, member 2
TUBA1A			Tubulin, alpha 1a
TUBB			Tubulin, beta
TUFM			Tu translation elongation factor, mitochondrial
TWSG1			Twisted gastrulation homolog 1 (Drosophila)
UBC			Ubiquitin C
UBE2E2			Ubiquitin-conjugating enzyme E2E 2
UBE2V2			Ubiquitin-conjugating enzyme E2V 2
UBL3			Ubiquitin-like 3
UBP1			Upstream binding protein 1 (LBP-1a)
UCN2			Urocortin 2
ULK1			Unc-51-like kinase 1 (C. elegans)
UNC45A			unc-45 myosin chaperone A
USMG5			Upregulated during skeletal muscle growth 5 homolog (mouse)
USP5			Ubiquitin specific peptidase 5 (isopeptidase T)
VARS			Valyl-tRNA synthetase
VARS2			Valyl-tRNA synthetase 2, mitochondrial (putative)
VCL			Vinculin
VCP			Valosin-containing protein
VIL2			Villin 2
WBP5			WW domain binding protein 5
WDR68			WD40 repeat-containing protein
WDR1			WD repeat domain 1
XPC			Xeroderma pigmentosum, complementation group C
XRCC6			X-ray repair complementing defective repair in Chinese hamster cells 6 (Ku autoantigen, 70kDa)
XRCC6BP1			XRCC6 binding protein 1

ZSWIM8			Zinc finger SWIM-type containing 8
ZYX			Zyxin

Supplementary Table S7

Gene	siRNA	OE	polysome UV/Asyn
ACTA2	X		**6.7**
ARPP19	X	X	**-2.6**
BMI1		X	**-2.2**
BTG2	X	X	3.0
CCND1	X	X	2.2
CDKN2AIPNL		X	2.0
CDKN1A	X	X	3.8
CEBPG		X	**-2.4**
CHCHD6 (PPP1R23)		X	**-3.0**
CMSS1		X	**-3.0**
COMMD1		X	**-2.1**
CTNNA1	X	X	2.0
DDB1	X	X	2.9
ENSA	X		
FABP7		X	**-4.1**
FAM129A	X	X	2.2
FBXO32	X	X	2.0
FEN1	X	X	2.2
FGF12		X	**-2.4**
GAK	X	X	2.6
GADD45A	X	X	2.0
GDF15	X	X	5.3
HSPA1A (HSP70)	X		3.1
HSPA1B (HSP70)	X		3.8
JUN	X	X	2.4
LARGE		X	**-2.1**
LIG1	X	X	2.0
LMNA	X	X	4.7
LZTS1 (FEZ1)	X	X	2.4
MASTL	X	X	
MGP		X	**-2.7**
NCOR2	X		3.1
NONO	X	X	2.2
PARP1	X	X	2.2
PCLAF	X	X	2.0
PHLDA3	X	X	2.0
PIAS4	X	X	2.5
POLG	X	X	2.1
PPP2R1A (PR65α)	X	X	2.1
PPP2R2A (B55α)	X		
RAC1		X	**-2.0**
RTCB	X	X	2.2
SCRIB	X	X	3.5
SH3BGRL		X	**-2.2**
SKP2		X	**-2.3**
SMARCA4 (BRG1)	X	X	2.1
SDHA	X		3.6

SKP2		X	**-2.3**
STAT3	X	X	2.0
SUMO		X	**-2.0**
TIMELESS	X	X	2.8
TP53I3 (PIG3)	X	X	2.6
UBC	X	X	2.0
UBE2E2		X	**-2.0**
UCN2		X	**-2.8**
USP5	X	X	2.8
VCP	X	X	2.3
XPC	X	X	2.3
XRCC6	X	X	2.0
XRCC6BP1		X	**-2.0**

Supplementary Table S8

Scoring scheme

siRNA	Cell Count	Foci Count	Cell Cycle	
	**1** if 1.5X >NT *OR* **2** if>2X >NT **1** if 1.5X <NT *OR* **2** if 2X <NT	**1** if p<0.01; **1** if 1.5X >NT *OR* **2** if 2X >NT **1** if 1.5X <NT *OR* **2** if 2X <NT	**1** for increased progression **2** for delayed progression	
**Con**	2	1+2	2	
**24 h UV**	2+1	1+2+1	2+1	add 1 for 24h
**40 h UV**	2+2	1+2+2	2+2	add 2 for 48h
**Total**	**9**	**12**	**9**	**Max scores**

OE	% V5 stained		Cell Cycle	
	**1** if 1.5X > Control *OR* **2** if>2X >Control **1** if 1.5X		**1** for increased progression **2** for delayed	
**24 h UV**	2+1		2+1	add 1 for 24h
**40 h UV**	2+2		2+2	add 2 for 48h
**Total**	**7**		**7**	**Max scores**

Supplementary Table S9

Results of Over-expression screen

	A2058			MM576			
	% trans	Cell cycle	TOTAL	% trans	Cell cycle	TOTAL	Localisation
ARPP19#*	2	0	2	3	0	3	Nuc.+Cyto; no change with UVR
**RTCB**	3	0	3	4	0	4	Nuc+cyto; no change with UVR
CMSS1#*	1	0	1	3	0	3	Grainy nuclear only; no change with UVR
CCND1*	2	0	2	0	0	0	Nuc.>cyto; no change with UVR
**CDKN1A***	4	3	7	3	5	8	Nuclear only; no change with UVR
**CDKN2AIPNL***	3	0	3	3	0	3	Mainly nuclear; no change with UVR
CEBPG#*	0	0	0	0	0	0	Nuc+cyto; no change with UVR
COMMD10#*	0	0	0	0	0	0	Nuc>cyto; no change with UVR
CTNNA1*	3	0	3	0	0	0	Nuc>cyto; no change with UVR
DDB1	1	0	1	3	0	3	Mainly nuclear; no change with UVR
**FAM129A**	3	0	3	3	0	3	Cytoplasm
**FBXO32***	4	0	4	3	0	3	Cytoplasm; no change with UVR
FEN1*	0	0	0	0	0	0	Nuclear; no change with UVR
GADD45A	2	0	2	4	0	4	Nuc>cyto; no change with UVR
GAK	3	0	3	0	0	0	Nuc, nuc+cyto; no change with UVR
GDF15*	4	0	4	2	0	2	Cytoplasm; no change with UVR
HSPA1A	4	0	4	1	0	1	Nuc>cyto; nuc level reduces with UVR
JUN*	1	7	8	0	4	4	Nuclear only; no change with UVR
PCLAF*	1	7	8	0	4	4	Nuc only; no change with UVR
LARGE#	2	0	2	0	0	0	Cytoplasm, Golgi? No change with UVR
LIG1	1	0	1	4	0	4	Nuclear only; small foci form after UVR, some co-localising with RPA foci.
LZTS1	4	0	4	1	0	1	Cytoplasm; filament (microtubule) associated? No change with UVR
MASTL	4	0	4	0	0	0	Nuclear; no change with UVR
MGP#*	2	0	2	1	0	1	Cytoplasm; no change with UVR
*NONO**	2	0	2	4	0	4	Nuclear; no change with UVR
PARP1	4	0	4	2	0	2	Nuclear; no change with UVR
PHLDA3*	3	4	7	0	7	7	Nuc>> cyto. No change with UVR.
*PIAS4**	4	0	4	2	0	2	Nuclear; no change with UVR
POLG	4	0	4	0	0	0	
RAC1#*	0	0	0	2	0	2	
**SCRIB**	4	0	4	4	0	4	Nuclear; no change with UVR
SH3BGRL#	2	0	2	3	0	3	Nuc>cyto; no change with UVR
SKP2#	2	0	2	1	0	1	Nuclear; no change with UVR
STAT3*	2	0	2	1	0	1	Nuc+cyto; changes in nuc to cyto ratio with UVR
TP5313	2	0	2	4	0	4	
UBE2E2#*	1	0	1	1	0	1	Mostly nuclear, some cells with fibrous cytoplasmic staining; no change with UVR
UCN2#*	1	0	1	0	0	0	Cytoplasm
USP5*	0	0	0	1	0	1	Nuc>cyto; no change with UVR

VCP	2	0	2	4	0	4	Nuclear; no change with UVR
XPC	3	0	3	0	0	0	Nuclear; no change with UVR
XRCC6	1	0	1	2	0	2	Nuclear; no change with UVR
*XRCC6BP1#**	4	0	4	2	0	2	Nuclear

Supplementary Table S10

Functional gene interactions

Gene	Function in UV/DNA damage responses	Interacting partners
**ARPP19**	PPP2CA inhibitor; acts through PPP2R2A and MASTL	PPP2CA
**BTG2**	Regulates MRE11 in DSB repair [2] and CAF1 deadenylation of mRNA [3].	SKP2, MRE11, CNOT7
**CCND1**	Reduced levels in UV treated cells [4]	BRCA1, CDKN1A, STAT3
**CDKN1A**	CDKN1A mRNA stabilisation regulated by ATR [5], and protein regulated by DDB2-DDB1 influencing TLS after UVR [6].	SMARCA4, GADD45A, PARP1, STAT3, XRCC6, FXBO32, HSPA1A, SKP2, VCP
**DDB1**	DDB1-DDB2 regulates CDKN1A/p21 degradation and switch to TLS after UV [7]	DDB2, SKP2, BRCA1, CDKN1A, RPA, XPC
ENSA		PPP2CA
FAM129A (NIBAN)	Phos by AKT after UVR; increases association with nucleophospmin disscoiating it from MDM2 leading to p53 degradation [42].	
FBXO32	Can degrade CDKN1A/p21 following DNA damage [8].	CDKN1A
FEN1	Okazaki fragment maturation and replication stress resolution [9].	LIG1, WRN
**GDF15**	Increased expression after UV [10].	
HSPA1A (HSP72)	Increased expression after UV [11], and required for DNA repair [12]	APEX1, CDKN1A, NONO, PPP2R1A, VCP
**JUN**	Increased expression drives apoptosis in high-dose UV [13].	BRCA1, PRKDC, STAT3, CDKN1A, PARP1
**LIG1**	Involved in Okazaki fragment maturation with FEN1, binds 9-1-1 complex in NER [14].	FEN1, MRE11A
LMNA	Lamin A mutants inhibit replication stress DNA repair after UV [15]	BMI1, BRCA1, MASTL, MRE11, NONO, PARP1, PRKDC, RPA1, VCP, XRCC6
**LZTS1 (FEZ1)**		
**MASTL**	Regulates exit from UV-G2 checkpoint	LMNA
**NONO**	Required for TOPBP1 loading onto ssDNA and ATR activation after UV [16].	RPA, SMARCA4, PCNA, HSPA1A, LMNA

PARP1	Recruits and PARylates XPC-HR23B to UV lesions [17, 18].	CDKN1A, PCNA, PRKDC, WRN, XRCC6, BRCA1, CHEK1, FEN1, LIG1, HSPA1A, JUN, LMNA, PIAS4, RPA, TIMELESS, XPC
***PCLAF* (PAF15)**	Is ubiquitinated and degraded after UV to allow TLS polymerase switching on PCNA sliding clamp [19].	PCNA
**PHLDA3**	PH-only domain protein, blocks AKT membrane binding and activity [20].	
PIAS4	SUMO E3 ligase required for RAD51 and MDC1 localisation to DSBs [21, 22].	BRCA1, MDC1, PARP1
PPP2R1A (PR65α)	Regulates DSB repair and G2 checkpoint response [23].	PPP2CA, PPP2R2A, HSPA1A, VCP
**PPP2R2A** (B55α)	Regulates PP2A binding and dephosphorylation of PLK1 [24] and MASTL dependent checkpoint recovery	PPP2CA, PPP2R1A, PLK1
**SCRIB**	Caspase substrate and cleavage product involved in apoptosis [25].	PPP1CA, PRKCA,
**SDHA**	Mitochondrial respiratory chain component	
**SMARCA4** (BRG1)	Associates with DDB2 and required for XPC loading onto DNA lesions. Suppress apoptosis, and restore checkpoint deficiency, and regulates CDKN1A and GADD45A expression in response to UV [26, 27].	CDKN1A, BRCA1, CDKN2A, NONO, STAT3
STAT3	STAT3 activation after UV [31], and regulates CDKN1A expression in concert with SMARCA4 [32].	BRCA1, CCND1, CDKN1A, RPA, APEX1, GADD45A, PRKCD, SMARCA4
**TIMELESS**	TIMELESS -TIPIN regulates ATR-CHK1 in replication stress and interacts with PARP1 independently at DNA lesions [33, 34].	TIPIN, ATRIP, CLSPN, CHK1, XRCC1
TP53I3	Required for CHK1/2 activation after UV; binds to DNA lesions and recruit repair proteins [35].	p53, PLK1, 53BP1
USP5	Recruited to DNA lesions by RAD18 [36].	RAD18, RAD23A/B,
**VCP**	Regulates ubiquitin-mediated degradation of XPC and DDB2 after UV [37], helicase unloading from stalled replication forks and loading of BRCA1, 53BP1, RAD51 onto DSBs [38, 39].	BRCA1, WRN, CDKN1A, CHEK1, CLSPN, CTNNA1, HSPA1A, PLK1, PPP2CA, PPP2R1A, PRKCD

**XPC**	GG-NER, interacts with DDB2 and SMARCA4	DDB2, RAD23A/B, ATR, DDB1, RPA,
XRCC6 (KU70)	Inhibits DDB2 autoubiquitination and blocks dissociation from lesion and blocking XPA association [40]. Also involved in ATR-CHK1 replication stress [41].	WRN, APEX1, CDKN1A, BRCA1, CHEK1, CTNNA1, DDB2, MRE11A, PARP1, RPA, WRN

Bolded - high confidence
Italics - lower confidence

References

1 Sanchez, A., et al., Proc Natl Acad Sci U S A., 2016. **113**(40): p. 11243-11248.

2 Choi, K.S., et al., DNA Repair (Amst). 2012. **11**(12): p. 965-75.

3 Stupfler, B., et al., Nat Commun., 2016. **7:** p. 10811.

4 Petrocelli, T. and J. Slingerland, Oncogene., 2000. **19**(39): p. 4480-90.

5 Al-Khalaf, H.H. and A. Aboussekhra, Mol Carcinog., 2014. **53**(12): p. 979-87. d

6 Bertolin, A.P., S.F. Mansilla, and V. Gottifredi, DNA Repair (Amst). 2015. **32:** p. 158-64.

7 Soria, G. and V. Gottifredi, DNA Repair (Amst). 2010. **9**(4): p. 358-64.

8 Wu, Z., et al., Cell Death Differ., 2011. **18**(11): p. 1771-9.

9 Dehe, P.M. and P.H. Gaillard, Nat Rev Mol Cell Biol., 2017. **18**(5): p. 315-330.

10 Yang, G., et al., J Invest Dermatol, 2006. **126**(11): p. 2490-506.

11 Zhou, X., et al., J Invest Dermatol., 1998. **111**(2): p. 194-8.

12 Gabai, V.L., M.Y. Sherman, and J.A. Yaglom, Oncogene., 2010. **29**(13): p. 1952-62.

13 Waster, P., I. Rosdahl, and K. Ollinger, Br J Dermatol., 2014. **171**(6): p. 1336-46.

14 Wang, W., et al., J Biol Chem., 2006. **281**(30): p. 20865-72.

15 Manju, K., B. Muralikrishna, and V.K. Parnaik, J Cell Sci., 2006. **119**(Pt 13): p. 2704-14.
16 Alfano, L., et al., Oncogene., 2016. **35**(5): p. 567-76.
17 Maltseva, E.A., et al., J Biol Chem., 2015. **290**(36): p. 21811-20.
18 Robu, M., et al., Proc Natl Acad Sci U S A., 2017. **114**(33): p. E6847-E6856.
19 Povlsen, L.K., et al., Nat Cell Biol., 2012. **14**(10): p. 1089-98.
20 Kawase, T., et al., Cell., 2009. **136**(3): p. 535-50.
21 Galanty, Y., et al., Genes Dev., 2012. **26**(11): p. 1179-95.
22 Shima, H., et al., J Cell Sci., 2013. **126**(Pt 22): p. 5284-92.
23 Wei, D., et al., Clin Cancer Res., 2013. **19**(16): p. 4422-32.
24 Wang, L., et al., Cell Cycle, 2015. **14**(1): p. 157-66.
25 Sone, K., et al., Genes Cells., 2008. **13**(7): p. 771-85.
26 Zhang, L., et al., PLoS One., 2014. **9**(8): p. e105764.
27 Zhao, Q., et al., J Biol Chem, 2009. **284**(44): p. 30424-32.
28 Al-Khalaf, H.H., S.F. Hendrayani, and A. Aboussekhra, Mol Cancer Res., 2011. **9**(3): p. 311-9.
29 Al-Khalaf, H.H., S.F. Hendrayani, and A. Aboussekhra, Mol Carcinog., 2012. **51**(12): p. 930-8.
30 Park, T.J., et al., Exp Cell Res., 2009. **315**(18): p. 3152-62.
31 Bito, T., et al., Exp Dermatol., 2010. **19**(7): p. 654-60.
32 Giraud, S., et al., Oncogene., 2004. **23**(44): p. 7391-8.
33 Smith, K.D., M.A. Fu, and E.J. Brown, J Cell Biol., 2009. **187**(1): p. 15-23.
34 Young, L.M., et al., Cell Rep., 2015. **13**(3): p. 451-9.

35	Lee, J.H., et al., Oncogene., 2010. **29**(10): p. 1431-50.
36	Nakajima, S., et al., PLoS One., 2014. **9**(1): p. e84899.
37	Puumalainen, M.R., et al., Nat Commun., 2014. **5:** p. 3695.
38	Fullbright, G., et al., Mol Cell Biol., 2016. **36**(23): p. 2983-2994.
39	Meerang, M., et al., Nat Cell Biol., 2011. **13**(11): p. 1376-82.
40	Takedachi, A., M. Saijo, and K. Tanaka, Molecular and Cellular Biology, 2010. **30**(11): p. 2708-2723.
41	Wang, H., et al., Cancer Res., 2002. **62**(9): p. 2483-7.
42	Ji et al., EMBO Rep., 2012. 13(6):554-60

Supplementary Table S11

DNA Damage Repair (DDR) Genes	Cell Cycle Checkpoint (CCC) Genes
DDB1	CCND1
DDB2	GDF15
RAD18	HSPA1A
FEN1	MASTL
PCLAF	ENSA
LIG1	ARPP-19
RPA2	LZTS1
WRN	BTG2
PHLDA3	STAT3
SMARCA4	FAM129A
XPC	CDKN2A
CDKN1A	PPP2R1A
TIMELESS	PPP2R2A
FBXO32	SCRIB
NONO	CHEK1
TP53I3	LMNA
JUN	PIAS4
VCP	BRCA1
XRCC6	ATR
USP5	PARP1
SDHA	
POLG	
RAD51	

Supplementary Table S12

**Mutational load**	***p value***
Low- High	0.00036
Mid - High	0.0076
Mid - Low	0.2
Low - Zero	0.26
Mid - Zero	0.018
High - Zero	9.9E-05
All group comparison	1.8 e-5

Supplementary Table S13

	**DDR genes**	**CCC genes**	**MASTL genes**
**Mutational load**	***p value***	***p value***	**p value**
Low- High	0.0000062	0.16	0.0014
Mid - High	0.0052	0.22	0.000033
Mid - Low	0.0076	0.91	0.99
Low - Zero	0.85	0.49	0.48
Mid - Zero	0.07	0.26	0.52
High - Zero	0.00047	0.039	0.0012
All group comparison	1.7 e-06	0.043	3.3 e-05

Supplementary Table S14

	p value			
Gene	Control -High	Low-High	Low-Mid	Control-Mid
BTG2	**0.0002**	0.6016	0.9641	**0.0003**
**PPP2R1A**	0.9976	**0.0132**	**0.0000**	0.9970
**MASTL**	**0.0032**	**0.0000**	**0.0061**	0.2868
RAD51	**0.0421**	0.1554	0.4661	0.1295
ATR	0.5885	**0.0358**	**0.0451**	0.8891
BRCA1	**0.0010**	**0.0075**	0.9731	0.1348
RPA2	0.3699	**0.0009**	0.2476	0.9998
**ARPP-19**	0.5761	**0.0080**	**0.0425**	0.9812
XRCC6	**0.0222**	0.9644	0.1837	0.2116
CDKN1A	0.6438	**0.0307**	0.9867	0.9474
RAD18	**0.0099**	**0.0066**	0.1968	0.1416
SDHA	0.7736	0.3355	**0.0106**	0.8490
JUN	0.2605	0.9995	0.7123	**0.0359**
PIAS4	0.4950	0.6743	**0.0068**	0.8328

Significance of the expression changes between USM groups

MASTL pathway genes bolded
